# Transcriptomic Profiling of JEG-3 cells using human leiomyoma derived matrix

**DOI:** 10.1016/j.bbiosy.2022.100056

**Published:** 2022-06-17

**Authors:** Samineh Barmaki, Ahmed Al-Samadi, Katarzyna Leskinen, Wafa Wahbi, Ville Jokinen, Sanna Vuoristo, Tuula Salo, Juha Kere, Satu Wedenoja, Päivi Saavalainen

**Affiliations:** a Department of Pharmacology, Faculty of Medicine, University of Helsinki, Helsinki 00290, Finland; b Department of Oral and Maxillofacial Disease, University of Helsinki, Helsinki 00290, Finland; c Translational Immunology Research Program, and Department of Clinical and Medical Genetics, University of Helsinki, Helsinki 00290, Finland; d Department of Chemistry and Materials Science, School of Chemical Engineering, Aalto University, Espoo 00076, Finland; e Obstetrics and Gynecology, University of Helsinki and Helsinki University Hospital, Helsinki 00290, Finland; f Department of Biosciences and Nutrition, Karolinska Institutet, Huddinge 14183, Sweden; g Folkhälsan Research Center, Helsinki 00290, Finland; h Stem Cells and Metabolism Research Program, University of Helsinki, Helsinki 00014, Finland; i Stem Cells and Metabolism Research Program, University of Helsinki, and Folkhälsan Research Center, Helsinki 00290, Finland

**Keywords:** Pre-eclampsia, Myogel, Hypoxia, Microfluidic, PDMS, Oxygen scavenger, JEG-3, Placenta, Microenvironment, Biomaterials

## Abstract

Oxygen tension varies during placental and fetal development. Although hypoxia drives early trophoblast invasion, low placental oxygen levels during pregnancy show association with pregnancy complications including fetal growth restriction and preeclampsia. JEG-3 cells are often used as a trophoblast model. We studied transcriptional changes of JEG-3 cells on a uterine leiomyoma derived matrix Myogel. This might be the closest condition to the real uterine environment that we can get for an in vitro model. We observed that culturing JEG-3 cells on the leiomyoma matrix leads to strong stimulation of ribosomal pathways, energy metabolism, and ATP production. Furthermore, Myogel improved JEG-3 cell adherence in comparison to tissue culture treated plastic. We also included PDMS microchip hypoxia creation, and observed changes in oxidative phosphorylation, oxygen related genes and several hypoxia genes. Our study highlights the effects of Myogel matrix on growing JEG-3 cells, especially on mitochondria, energy metabolism, and protein synthesis.

## Highlights

• JEG-3 cells - from choriocarcinoma cell line, on uterine leiomyoma derived matrix, i.e., Myogel, presents a real environment that we can get for an invitro model for early trophoblasts.
• Myogel coating on Poly Di-Methyl Siloxane (PDMS) reverts hydrophobic surface of PDMS to hydrophilic. Therefore, cells grow in monolayer structure on PDMS.
• Preeclampsia, known as the placenta disorder, is influenced by oxygen stress and hypoxia. To model microenvironmental preeclampsia in placenta, JEG-3 cells with Myogel matrix are cultured on our previously reported PDMS microchip which generates hypoxia microenvironment.

## Graphical abstract

**Figure:**
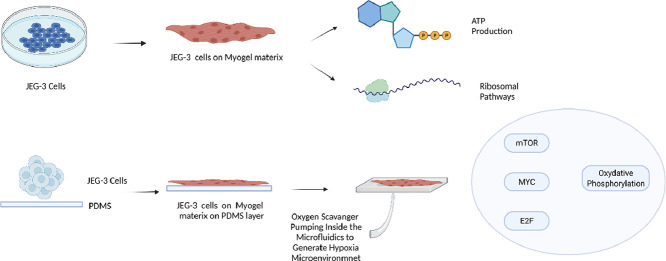

## 1 Introduction

Trophoblasts are temporary cells that form the epithelial layer of the placenta. Deep invasion of fetal trophoblasts into the uterine wall of the mother is a crucial step of the placentation to deliver adequate blood and oxygen supply to the fetus. Defects in the trophoblast invasion during early pregnancy limit the placental development and capacity and might underlie a spectrum of human pregnancy complications, from miscarriages to fetal growth restriction and preeclampsia [1].

Many problems in human pregnancy show association with chronic placental hypoxia. Early stages of placentation are also accompanied with a decline in the oxygen levels at the maternal-fetal interface. This physiological hypoxia results in deeper invasion of extravillous trophoblasts into the wall of the uterus in search for oxygen, and formation and remodeling of the spiral arteries. In later stages of human pregnancy, however, trophoblasts are unable to invade and placental hypoxia leads to inflammation, oxidative stress, and apoptosis [2], [3], [4], [5], [6], [7], [8], [9].

Studying the earliest stages of placentation remains challenging. There are several reason for it. First, primary trophoblasts isolated from human first-trimester placentas do not proliferate in vitro [10]. Second, different hypoxia systems such as low-oxygen chambers and chemicals provide partly inconsistent findings [11]; and finally, the medium used in hypoxia experiments may further modify cellular responses [12]. To date, many studies have utilized JEG-3 chorioncarcinoma cell line as a model for early trophoblasts [10], [13], [14], [15].

Hypoxia-induced changes in JEG-3 cells involve reduction of the major placental hormone human chorionic gonadotropin (hCG), upregulation of the vascular endothelial growth factor (VEGF) [8], [10], and transcriptional regulation of a number of other genes by Hypoxia Inducible Factors (HIFs) [16]. However, results vary depending on the applied hypoxia system [17], [18], [19], [20], [21].

We have previously generated a microfluidic chip setup, where hypoxia is generated by pumping oxygen scavenger solution into a microfluidic channel that is separated from the cell culture by a gas-permeable membrane [22], [23].

We hypothesized that this system could be used to model hypoxia-induced gene expression changes of human trophoblast.

We studied RNA expression of JEG-3 cells using the hypoxia microchip we developed, and human leiomyoma- derived matrix Myogel, which mimics tumor micro-environment and the human uterine wall [24], [25]. Also, we present cellular responses and the genes that are differentially expressed during hypoxia, and the utility of Myogel as an extracellular matrix for the JEG-3 culture.

## 2 Materials and methods

### 2.1 Cell culture

All experiments were performed using JEG-3 cells (RRID: CVCL-0363). These cells were cultured using Eagles minimum essential medium^3^ with non-essential amino acids, 90% sodium pyruvate, and 10% antibiotic-free Fetal bovine serum (FBS, Gibco). Cells from passages 5–12 were used for experiments. All cell culture experiments were performed using a regular cell incubator ($37^{\circ }$ with $5\%\;CO_{2}$).

### 2.2 Testing different coating on JEG-3 cells

24-well plates^4^ were coated with poly-dimethyl-siloxane (PDMS). PDMS consists of two parts of polymer (Base Elastomer and Curing Agent). We used the standard mixing ration for PDMS, 10 parts base elastomer and 1 part curing agent (10:1) to coat the cell culture well plates. Then 150μl of PDMS added to the 24 well plates (surface area: $1.9cm^{2}$). We used the same PDMS ratio (10:1) to fabricate our previous reported microchips and the PDMS membrane).

Then, 0.5 mg/ml Myogel (50ôl/well) and/or fibrin matrix were added on PDMS, while control wells remained uncoated. For stabilizing the coating, the plates were incubated in $37^{\circ }$ with $5\%\;CO_{2}$ for 24 hours. JEG-3 cells were seeded 24 hours after coating of the plates. The cells were lysed after the first and third day for RNA analysis. All experiments were performed in three technical replicates.

We collected the cells on day 1 and day 3 to evaluate RNA concentrations. Because of the observed increase in RNA yields from day 1 to 3, and for the normal morphology of cells on day 1, we then performed RNA analyses on day 1 cells. Moreover, for the hypoxia experiment, we used an incubation time of 24 hours (day 1), which has been used in several earlier hypoxia studies on JEG-3 cells [9], [26].

### 2.3 PDMS microchip fabrication

The PDMS chips were fabricated by replication molding from SU-8 masters. The fabrication method and design of the microfluidic chips have been described previously [22]. Briefly: The SU-8^5^ was spin coated 4000 rpm 30s for a 40μm thick layer. The chips were made from PDMS^6^ with the ratio of 10:1 with the cross-linking agent. The chips were closed by bonding a 30μm thick PDMS membrane on top of the channels. The PDMS membrane was fabricated by spin coating PDMS 2000rpm 30s on top of an anti-adhesive fluoropolymer coats silicon wafer.

### 2.4 Microchip assay

The surface adherence plays essential role in PDMS attachment on PDMS reservoir [27]. The microfluidic chips and cell reservoir were washed gently with soap and rinsed with deionized water and 70% ethanol. Then, they were dried with pressurized air; a piece of scotch tape was used to remove any residual dust or particles from the surface. For testing the functionality of microfluidics before cell culture experiments, water was pumped with a microfluidic syringe pump^7^ with flow rate of 0.1ml/h (1.6μl/min) into the microfluidic channels. If any leaks was detected during the test the chip is discarded.

A 2D cell culture matrix with Myogel was generated on PDMS membrane of microfluidic chip. The oxygen scavenger solution was pumped for 24hours in the two meanders of the microchip with the flow rate of 0.1ml/h. Hypoxia was induced on the cells that were separated from an underlying oxygen scavenging channel network by a thin PDMS membrane, as described previously [22], [23].

**Fig. 1:**
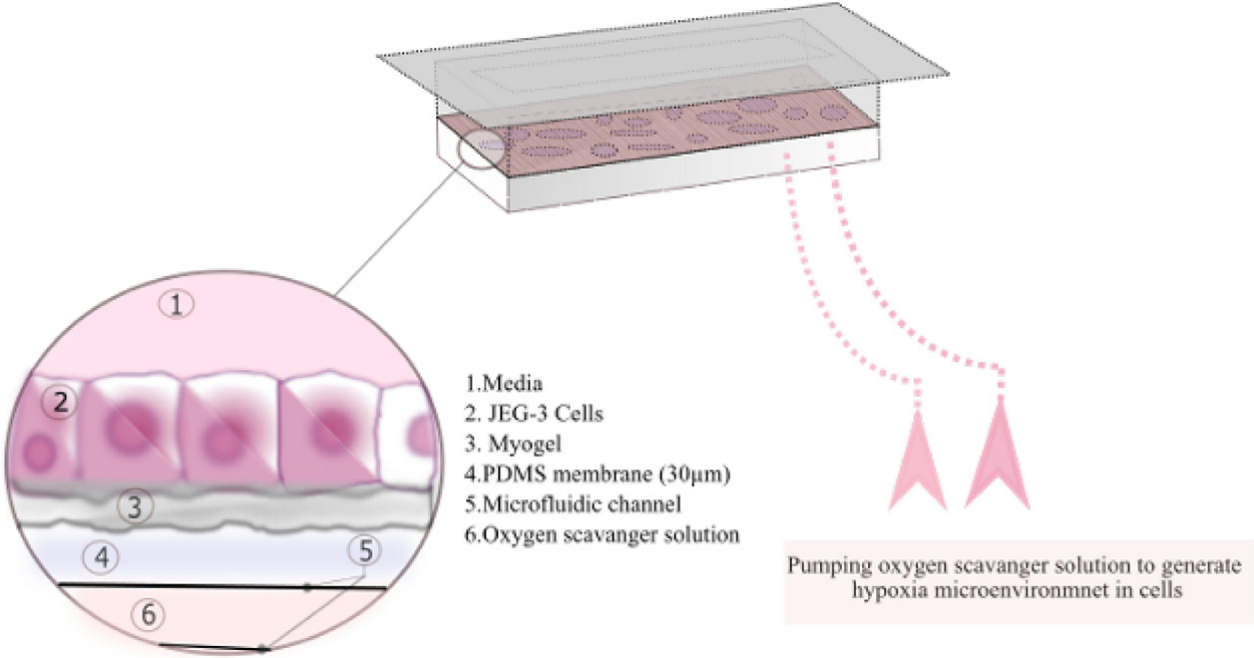
Structure of the hypoxia microchip.

For making oxygen scavenger solution a water solution of sodium sulfite (835mM oxygen-depleted $H_{2}O$) was used to generate the oxygen sink. The reaction of sodium sulphite ($Na_{2}SO_{3}$) with dissolved oxygen in $H_{2}O$ is utilized for oxygen depletion and generation of an active in-liquid oxygen sink. Cobalt nitrate ($Co\;{\left(NO_{3}\right)}_{2}$ hexahydrate salt, 9mM) in nitric acid was used as the catalyst. All reagents were obtained from Sigma Aldrich^8^. Fig. 1 illustrates the structure of the hypoxia microchip, where hypoxia was generated by pumping of the oxygen scavenger solution in the microfluidic channels under the JEG-3 cells.

### 2.5 RNA-sequencing and data analysis

RNA was extracted from the JEG-3 cells using the RNA easy Mini Kit^9^ according to the manufacturers instructions. Cells were homogenized with QIAshredder columns (Qiagen). After extraction, RNA was quantified using Qubit 2 Fluorometer^10^ with the Qubit RNA HS Assay Kit^11^. RNA samples were stored at $-70^{\circ }C$. The RNA sequencing method was designed based on the Drop-seq. protocol, as described [20]. Briefly speaking, 10ng of RNA was mixed with Indexing Oligonucleotides^12^. After 5 minutes of incubation at ambient temperature, RNA was combined with RT mix containing 1× Maxima RT buffer, 1mM dNTPs, 10U/μl Maxima HRTase, 1U/μl RiboLock^13^, and 2.5μM Template Switch Oligo^14^. Samples were incubated in a T100 thermal cycler^15^ for 30 minutes at $22^{\circ }C$ and 90 minutes at $52^{\circ }C$. The constructed cDNA was amplified by PCR in a volume of 15μl using 5μl of RT mix as template, 1× HiFi HotStart Ready-mix^16^, and 0.8μM SMART PCR primer. The samples were thermo-cycled in a T100 thermocycler as follows: $95^{\circ }C$ 3 min; subsequently four cycles of $98^{\circ }C$ for 20 sec, $65^{\circ }C$ for 45 sec, $72^{\circ }C$ for 3 min; following 16 cycles of $98^{\circ }C$ for 20 sec, $67^{\circ }C$ for 20 sec, $72^{\circ }C$ for 3 min; and with the final extension step of 5 min at $72^{\circ }C$. The PCR products were pooled together in sets of 27 samples containing different Indexing Oligos and purified with 0.6X High-Prep PCR Clean-up System^17^ according to the manufacturers instructions. They were eluted in 10μl of molecular grade water. The 3-end cDNA fragments for sequencing were prepared using the Nextera XT^18^ tagmentation reaction with 1ng of each PCR product serving as an input. The reaction was performed according to the manufacturers instructions, with the exception that the P5 SMART primer was used instead of the i5 Nextera primer. After the PCR reaction, each set of samples was pooled and tagmented with a different Nextera i7 index. Subsequently, the samples were PCR amplified as follows: $95^{\circ }C$ for 30 sec; 11 cycles of $95^{\circ }C$ for 10 sec, $55^{\circ }C$ for 30 sec, $72^{\circ }C$ for 30 sec; with the final extension step of 5 min at $72^{\circ }C$. Samples were purified twice using 0.6× and 1.0× High-Prep PCR Clean-up System (MagBio) and eluted in 10μl of molecular grade water.

The concentration of the library was measured using a Qubit 2 Fluorometer^19^ and the Qubit DNA HS Assay Kit^20^, while the quality was assessed using the LabChip GXII Touch HT electrophoresis system^21^, with the DNA High Sensitivity Assay (PerkinElmer) and the DNA 5K/RNA/Charge Variant Assay LabChip (PerkinElmer). Samples were stored at $-20^{\circ }C$. The libraries were sequenced on an Illumina NextSeq 500, with an addition of the custom primer producing a read 1 of 20bp and a read 2 (paired end) of 55bp. Sequencing was performed at the Functional Genomics Unit of the University of Helsinki. The RNA sequencing data has been deposited to NCBI Gene Expression Omnibus (Accession ID: GSE182988).

Raw sequence data was filtered to remove reads shorter than 20bp. Subsequently, the original pipeline for processing of Drop-seq data was used [28]. Briefly, reads were additionally filtered to remove poly A tails of length 6 or greater, and aligned to the human (GRCh38) genome using STAR aligner with default settings [29]. Uniquely mapped reads were grouped according to the barcodes 1 to 9, and gene transcripts were counted by their Unique Molecular Identifiers (UMIs) to reduce bias emerging from the PCR amplification. Digital expression matrices (DGE) were used for reporting the number of transcripts per gene in each sample (according to the distinct UMI sequences counted). Differentially expressed genes were identified using DESeq2 [30] with the cut-off for the adjusted p-value set to 0.05. We compared our hypoxia microchip data on JEG-3 RNA expression with an independent publicly available dataset. RNA-seq data on expression of JEG-3 cells cultured in a hypoxia chamber for 24 hours [31] were downloaded from the GEO under the accession number GSM1862652.

Normalization and differential expression analysis between these two data sets were performed using edgeR [32] package integrated into Chipster platform [33]. Heatmaps were drawn with heatmapper tool [34] and pathway analyses were performed with Enrichr (MSigDB Hallmark 2020 pathway) [35], [36].

## 3 Results

### 3.1 Influence of Myogel coating on gene expression and cell morphology

**Fig. 2:**
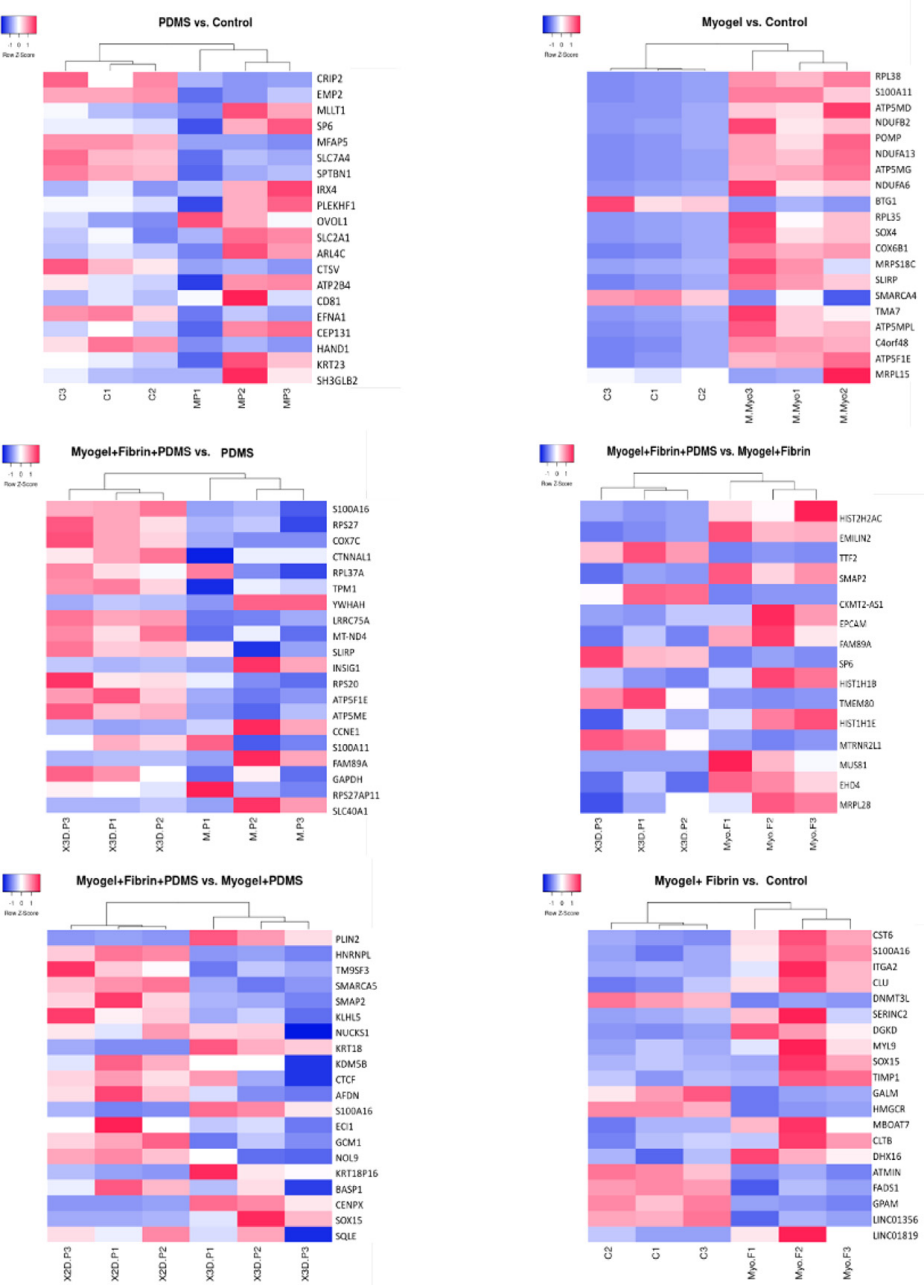
Comparison of differentially expressed genes between the well plates coated with PDMS, Myogel, fibrin, and their combinations. Heat-maps show the top 20 differentially expressed genes (with 2-fold change or greater) in JEG-3 cells for each conditions after hierarchical clustering analysis. Each column represents three replicates for each of the two conditions (M.P: PDMS, C: Control, Myo.F: Myogel + Fibrin (Myo: Myogel, F: Fibrin), X3D.P: Myogel + Fibrin + PDMS,(X3D: Myogel+Fibrin, P: PDMS) X2D.P: Myogel + PDMS (X2D:Myogel,P: PDMS), M.Myo : Myogel), and the rows indicate the genes. The gene expression levels were shown by color and intensity: red indicates down-regulation and blue upregulation, and higher color intensity reflects higher fold change. Statistical analysis done by heatmapper, Three replicates from each condition, (n=3). (For interpretation of the references to colour in this figure legend, the reader is referred to the web version of this article.)

**Fig. 3:**
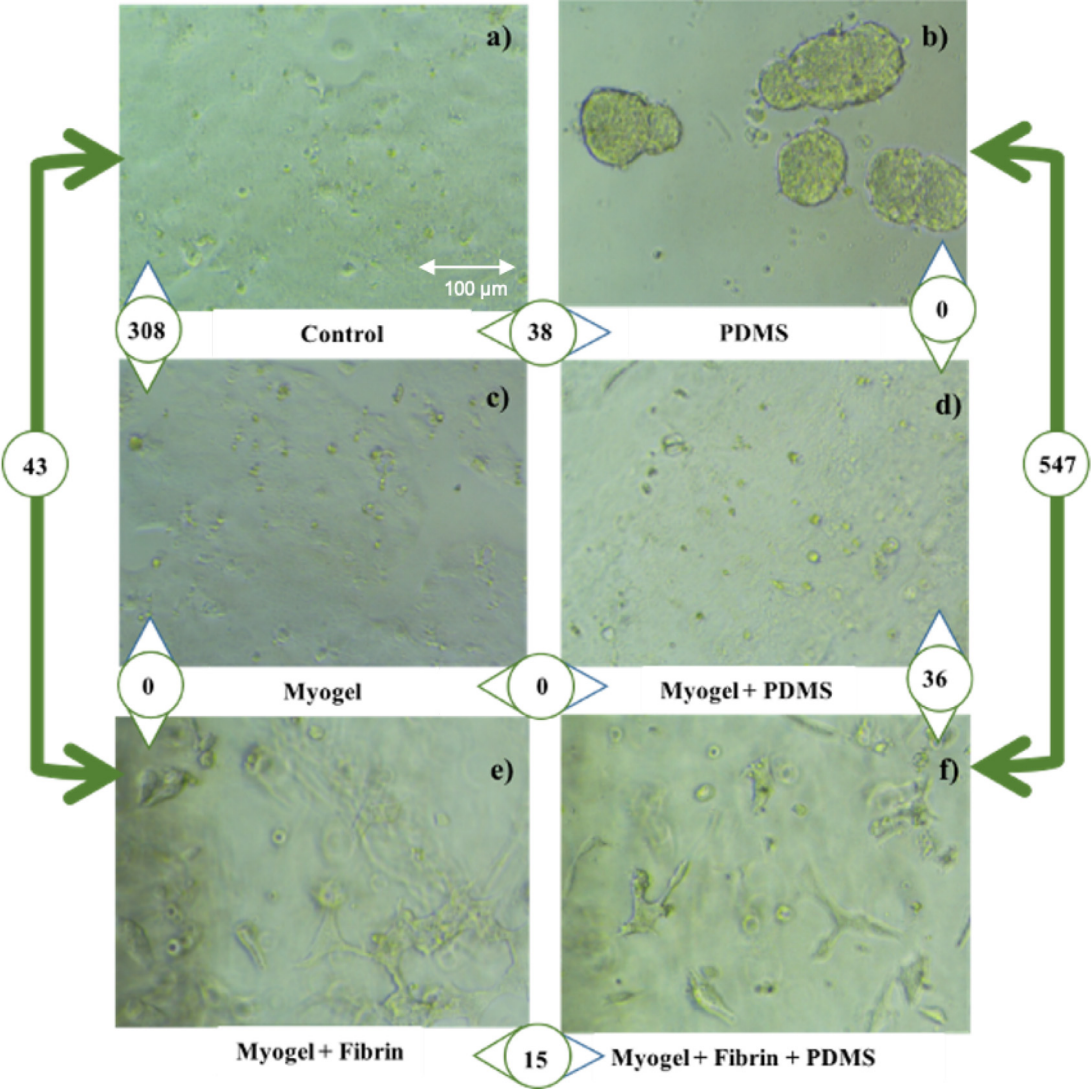
Morphology and the number of significantly differentially expressed genes in JEG-3 cells in the cultures a) Control (only medium),308 significant gene expressed in Myogel over control, 38 genes expressed in PDMS over control, 43 genes expressed in Myogel + Fibrin over control. b) PDMS,not significant genes expressed between PDMS and Myogel + PDMS, 547 genes expressed in Myogel+Fibrin+PDMS over PDMS. c) Myogel,not significant genes expressed in Myogel + Fibrin over Myogel and Myogel + PDMS over Myogel. d) Myogel + PDMS,36 genes expressed in Myogel+Fibrin+PDMS over Myogel + PDMS. e) Myogel + fibrin,15 genes expressed in Myogel + Fibrin + PDMS over Myogel + Fibrin. f) Myogel + fibrin + PDMS. Three replicates from each condition (n=3).

We assessed the morphology and RNA expression of JEG-3 cells at the first day of the cell culture. We compared cell morphology and analyzed gene expression of the JEG-3 cells cultured in the wells coated with Myogel, PDMS, Myogel + PDMS, Myogel + fibrin, Myogel + fibrin + PDMS, and uncoated control, (Fig. 3). Total RNA yields increased in extractions from day one to day 3 (Supplementary Table S10), indicating that the cells were proliferating. The genes and corresponding pathways modulated by different conditions were studied. Some of the most significant findings were in association with Myogel coating, (Table 1 ; Fig. 2). When Myogel was added to the JEG-3 culture, 308 genes showed differential expression (Supplementary Table S2), and Myogel provided better cell adhesion (Fig. 3c).

**Table 1:** Up-regulated Pathways of JEG cells on well plate experiment.

**PDMS*****v.s.*****Control**			**Myogel*****v.s.*****Control**		
Term	P-value	Genes	Term	P-value	Genes
Coagulation	0.008	CRIP2; CTSV	Oxidative Phosphorylation	≈0	COX7B; SLC25A3; ECHS1; NDUFB7; NDUFB3; NDUFB; NDUFV1; ATP5G; ATP5ME; COX8A; TIMM8B; ATP5P
Mitotic Spindle	0.016	CEP131; SPTBN1	Myc Targets V1	≈03	DDX18; SLC25A3; CNBP; RPLP0; ILF2; SRPK1; LSM2; PSMD8; CDC20; AIMP2; SYNCRIP; FBL; LSM7; C1QBP; NDUFAB1; POLD2; RACK1; SRSF2; SNRPG; SRSF7; EIF4G2
Hypoxia	0.016	EFNA1; SLC2A1	Adipogenesis	≈0	COX8A; SLC25A1; COX7B; MTCH2; ECHS1; NDUFB7; ITSN1; ECH1; TALDO1; CHCHD10; UQCR11; ATP1B3; MRPL15; UQCR10; UQCRQ; NDUFAB1; UQCRC1; SUCLG1
Estrogen Response Late	0.016	ATP2B4; EMP2	E2F Targets	0.001	CDC20; SYNCRIP; ILF3; POP7; POLD2; SRSF2; PSIP1; MKI67; DCTPP1; RAN
TGF-beta Signaling	0.052	SPTBN1	Peroxisome	0.001	FDPS; VPS4B; CNBP; ECH1; SLC25A4; FADS1; YWHAH
PI3K / AKT / mTOR Signaling	0.099	SLC2A1	Myc Targets V2	0.001	DDX18; AIMP2; PPAN; FARSA; DCTPP1
UV Response Dn	0.134	ATP2B4	Fatty Acid Metabolism	0.003	ECHS1; NTHL1; UROS; ECH1; SUCLG1; PRDX6; HSD17B10; YWHAH
UV Response Up	0.146	CTSV	G2-M Checkpoint	0.003	CDC20; TPX2; SYNCRIP; PRMT5; GINS2; ILF3; UBE2C; SRSF2; MKI67
IL-2 / STAT5 Signaling	0.181	CD81	Cholesterol Homeostasis	0.005	FDPS;EBP;ECH1; HMGCR; S100A11
TNF-alpha Signaling via NF-kB	0.182	EFNA1	Unfolded Protein Response	0.008	RPS14; NOP14; EXOSC4; IMP3; POP4; HSP90B1
p53 Pathway	0.182	CD81	DNA Repair	0.028	NT5C; MPG; ELOA; SAC3D1; ITPA; POLR2K
heme Metabolism	0.182	SLC2A1	Androgen Response	0.068	KRT19; UBE2I; HMGCR; FADS1
Estrogen Response Early	0.182	SLC2A1	Estrogen Response Late	0.09	CDC20; GINS2; KRT19; PERP; CAV1; DCXR
Complement	0.182	CTSV	mTORC1 Signaling	0.09	CCT6A; EBP; HMGCR; FADS1; HSP90B1; ATP5MC1
mTORC1 Signaling	0.1821	SLC2A1	p53 Pathway	0.09	LDHB; BTG1; PERP; DCXR; RGS16; RACK1
Epithelial Mesenchymal Transition	0.182	MFAP5	UV Response Up	0.09	NFKBIA; EIF5; BTG1; EPCAM; SLC25A4
			Reactive Oxygen Species Pathway	0.17	NDUFA6; PRDX6
			Protein Secretion	0.18	SGMS1; VPS4B;CLTA
			Mitotic Spindle	0.193	TPX2; PPP4R2; ITSN1; CKAP5; SAC3D1
			Hypoxia	0.196	EFNA1; SLC25A1; BTG1; ANXA2; CAV1

The gene expression changes that were observed were related to several neurological diseases (Huntington disease, Parkinson disease, and Alzheimer disease). This might reflect the crucial role of oxygen supply in different tissues, including both the placenta and brain. This finding indicates that the changes we observed were not trophoblast specific but more related to general changes in energy metabolism. These pathways were tightly related to several NDUF genes (NDUFB7; NDUFB3; NDUFB; NDUFV1) mitochondrial respiratory genes, and ribosomal protein metabolism. We also assessed how PDMS and fibrin - or their combinations - modulate gene expression when they were used with Myogel. When the cells were coated with the combination of Myogel, fibrin, and PDMS, we observed significant modulation of ribosomal genes. This observation was similar to what has been seen with pure Myogel; accordingly, fibrin itself has very small effects on gene expression, (Table 1, Fig. 2). This was particularly demonstrated between the conditions PDMS + Myogel vs. PDMS + fibrin + Myogel, showing almost no changes in gene expression. However, the comparison between Myogel + fibrin + PDMS vs. PDMS only produced significant changes in gene expression (Table 1, Fig. 2 and 3).

Although the Myogel culture-associated pathways indicated significant changes, single genes were not changed to the same extent. Analysis of differentially expressed genes revealed upregulation of hypoxia pathway with two genes associated with PDMS coating vs. control: EFNA1 and SLC2A1 (P-value: 0.016), (see Fig. 2). This may be caused by hydrophobic surface property of PDMS that induces organoid type of structures (Fig. 3b), suggesting that oxygen might not diffuse freely in the cells. However, hypoxic pathway was also up-regulated by Myogel vs. pure medium, as seen about the genes EFNA1, SLC25A1, BTG1, ANXA2, and CAV1 (P-value: 0.196). Interestingly, adding Myogel on the PDMS coated culture prevented the development of organoid structures (see Fig. 3d), but showed no clear changes in gene expression. In contrast, adding fibrin to Myogel-coated culture resulted in no changes in gene expression, but three dimensional organization of the cells was observed (Fig. 3e).

Detailed information of well-plate experiment, such as DE-gene lists (Supplementary Tables S3-S9) and significant genes in one heat-map (see Supplementary Figure S3) with Venn diagram of important pathways of well-plate experiment (Supplementary Figure S4) showed in the supplementary section.

### 3.2 Genes and pathways in Myogel coated hypoxia microchip

The data suggested that Myogel enhances cell adherence on PDMS, without inducing changes in gene expression. The cell adhesion was visually suspected because they were tightly adherent, when collected for RNA extraction. These results prompted us to use Myogel coating on PDMS microchips in hypoxia experiments. We assessed the genes and pathways that were modulated in Myogel coated PDMS microchips and exposed to oxygen scavenger for 24 hours. The chip generates oxygen levels of <5%, as shown in our previous study, through the activation of image-iT® red fluorescent marker. Because of the formation of organoid structures, we could not use pure PDMS as a control in these experiments, (see Fig. 3a and b). We used Myogel on PDMS microchip, as we observed that PDMS had no effect on gene expression when it was used in addition to Myogel, (Fig. 3c and d). Moreover, liquid form of pure Myogel, in contrast with respect to the thick gel formed with fibrin, allowed the cells to adhere on the PDMS membrane as a single layer, exposing the cells evenly to hypoxia.

In normoxia, the cells were cultured on a microchip pumped with water, whereas in hypoxia, the microchip was pumped with oxygen scavenger solution for 24 hours. Principal component analysis (PCA) of samples cultured under hypoxia or normoxia samples microchips revealed a clear separation between the samples, as expected (Supplementary Figure S1).

## 4 Discussion

Ribosome biosynthesis, the top pathway modulated by JEG-3 cells cultured on Myogel, plays a role in protein synthesis, cell growth, and tumorigenesis [35]. Our findings indicate significant metabolic changes when Myogel was used as a matrix of a JEG-3 cell culture. These data suggest that Myogel promotes JEG-3 cells growth and differentiation, and regulates pathways that are associated with human diseases.

Adding PDMS on Myogel + fibrin coating showed no obvious changes in cell morphology and no organoid structures, but 36 genes were up-regulated. Collectively, these morphological findings are in line with the previous observations, showing that Myogel provides better cell migration, and Myogel + fibrin coating generates two and three dimensional matrices [24], [25], [36]. Considered together, these data indicate that the coating has a marked impact on gene expression and morphology, including key cellular processes such as ribosome biosynthesis (mainly Myogel related effect), endocytosis, and adhesion-associated genes such as several of the matrix metalloproteases [37], [38]. The main pathways that were repressed by hypoxia microchip included mTOR (Mammalian target of rapamycin), MYC (Myelocytomatosis oncogene), E2F (Early 2 factor), and oxidative phosphorylation. Because MYC is a major cell proliferation marker, upregulation of MYC together with G2M checkpoint support that the cells start to proliferate faster in response to hypoxia, see Supplementary Figure S2. These findings point toward reprogramming of cancer cell metabolism, tumor cell growth and progression, and mitochondrial metabolism [38], [39], [40], [41], [42], [43].

We observed altogether 306 differentially expressed genes in JEG-3 cells in hypoxia vs. normoxia (Supplementary Table S1), including upregulation of the mitochondrially encoded genes such as MT-RNR2, MT-ND4, MT-CO2, and MT-CYB, and the lncRNA gene MALAT1. These genes play key roles in mitochondria metabolism, which might reflect the effects of the limited oxygen availability [44]. The most significantly down-regulated genes during hypoxia included UQCR11, IP6K1, STK17A, NFE2L2, and C1QBP. These genes activated oxidative stress, MYC targets (v1,v2), and TNF alpha/NF-kB, all of which modulate cell proliferation, differentiation, apoptosis and coagulation [45], [46], [47], [48], [49], [50], [51].

Although the pathways associated with cell proliferation were activated in response to oxygen scavenger, we found no significant enrichment of the hypoxia pathway genes in the Enrichr pathway analysis. However, five individual hypoxia related genes showed significant upregulation: AKAP12, TPI1, ZNF292, ENO1, and GAPDH. We further compared our genes and pathways modulated in the hypoxia microchip experiment by reanalyzing the results of Zhu et al. [52]. They have studied the effect of hypoxia using JEG-3 cells in a hypoxia chamber for 16 hours. In their experiment, 131 genes were differentially expressed. Of these genes, 14 were differentially expressed also on our microchip, and 12 of these showed effects to the same direction, although the expression fold changes were smaller with our microchip (see Table 2). The most important pathways that were linked to differentially express genes in both our study and by Zhu et al. were oxidative phosphorylation, E2F targets, P53 pathways, apoptosis, hypoxia, and PI3K/AKT/mTOR signaling. Altogether, our study found genes from 33 shared pathways that were observed in the study of Zhu et al.,see Supplementary Table S2. Notably, MYC pathway was similarly enriched in both studies. Although only few genes from hypoxia pathway were observed in our microchip experiment, we observed changes in the expression of mitochondrial genes (MT-RNR2, MT-ND4, MT-CO2, and MT-CYB). This could be related to oxidative stress and reflect the cellular environment in our experiment.

**Table 2:** 14 genes that were differentially expressed both in our microchip experiment and in the dataset of Zhu et al. Log2 fold changes and adjusted P-values are compared, and known pathways involved are shown.

	Our study		Zhu et al.		MSigDB pathways involved (Enrichr)							
DE genes in both sets	log2 FC	p(adj)	log2 FC	p(adj)	Hypoxia	mTORC1 Signaling	Myc Targets V1	p53 Pathway	Apoptosis	Glycolysis	E2F Targets	G2-M Checkpoint
RPS27	1.24	5.15E-08	1.01	3.33E-02								
FTH1	1.10	6.86E-05	1.43	2.85E-04								
RPS24	0.93	2.65E-04	1.03	2.54E-02								
CANX	1.00	4.43E-04	1.56	1.07E-02		x	x					
RPS27A	0.76	2.04E-03	1.33	1.70E-02								
GAPDH	0.84	2.22E-03	1.85	7.22E-07	x	x					x	
HMGB2	0.76	5.35E-03	3.63	3.82E-04					x		x	
JUND	0.71	1.14E-02	1.82	1.89E-03								
SFPQ	0.65	1.22E-02	2.32	2.26E-03								x
CCSAP	0.63	1.87E-02	1.90	1.46E-02								
GM2A	0.56	2.88E-02	1.52	2.07E-03				x				
ENO1	0.52	3.06E-02	4.22	3.98E-14	x	x				x		
RACK1	0.59	3.85E-02	1.01	3.66E-02			x					
TPI1	0.57	4.59E-02	1.29	6.59E-03	x	x				x		

The non-significant effects of our microchip on hypoxia pathway might be related to Myogel, microchip setup, or the duration and the level of hypoxia. In contrast with hypoxic chambers, our microchip involves oxygen deprivation from the apical surface of the cells, which is in contact with the PDMS membrane. It is therefore possible that the luminal side of the cells gets more oxygen from the culture media, and results in only a modest change of hypoxia induced gene expression. The other possibility for the insufficient hypoxia response on our microchip setup could be the chemical reactions of the oxygen scavenger solution. Hypoxic condition are generated by the conversion of sodium sulphite to sodium sulphate (Eq. 1) [53]. Furthermore, sodium sulphite generates sulphur dioxide ($SO_{2}$) by reacting with water (Eq. 2) [54]. Since PDMS is gas permeable, $SO_{2}$ could cross PDMS membrane and influence the significance of generation of hypoxia on the microchip system.

(1) $$2Na_{2}SO_{3}+O_{2}⟶2Na_{2}SO_{4}$$

(2) $$Na_{2}SO_{3}+H_{2}O⟶SO_{2}+2NaOH$$

Although we were unable to measure the exact level of hypoxia on the microchips along with RNA analyses, our previous studies support low oxygen levels ($O_{2}\leq 5\%$) produced by the microchip [22], [23].

It also remains possible that Myogel prevented or modulated the expected effects of the oxygen scavenger. Remarkably, uterine myomas might be hypoxic as such [55], and this could further interfere with the effects of the hypoxia on Myogel coated microchips in comparison with the previous studies.

## 5 Conclusion

This study shows that JEG-3 cells cultured on the Myogel matrix associate with strong stimulation of ribosomal pathways, energy metabolism, and ATP production. Myogel matrix provides better JEG-3 cell adherence on PDMS and shows significant effects on cell morphology and RNA expression. Although our results did not show significant upregulation of the hypoxia pathway genes using the oxygen scavenger and the microchip setup, significant upregulation of several individual hypoxia-related genes and the oxidative phosphorylation pathway arose. These results provide important insights into the possibility to modulate cellular processes and gene expression by different coating. Moreover, our results support the utility of cellular microenvironments in JEG-3 studies. Further studies are needed to assess the effects of temporary and chronic hypoxia in the cell morphology, invasive capacity, and gene expression.

## Funding

This research was funded by University of Helsinki - Doctoral program of Health science.

## Declaration of Competing Interest

The authors declare the following financial interests/personal relationships which may be considered as potential competing interests:

Samineh Barmaki reports financial support was provided by University of Helsinki. Samineh Barmaki reports a relationship with University of Helsinki that includes: employment and funding grants.

## Appendix A Supplementary materials

Supplementary Data S1 Supplementary Raw Research Data. This is open data under the CC BY license http://creativecommons.org/licenses/by/4.0/ Supplementary Data S1

Supplementary Data S2 Supplementary Raw Research Data. This is open data under the CC BY license http://creativecommons.org/licenses/by/4.0/ Supplementary Data S2

## Footnotes

3 MEM, Gibco, Thermo Fisher Scientific Inc, Waltham, MA

4 Thermo Fisher Scientific Inc, Waltham, MA

5 SU-8 50, Kayaku Advanced Materials, Westborough, MA

6 Sylgard 184, Merck, KGaA, Darmstadt, Germany

7 NE-4000, New Era Pump Systems Inc., Farmingdale, NY

8 St. Louis, MA

9 Qiagen, Hilden, Germany

10 ThermoFisher Scientific

11 ThermoFisher Scientific

12 Integrated DNA Technologies

13 ThermoFisher Scientific

14 TSO, Integrated DNA Technologies

15 BioRad, Hercules, California, United States

16 Kapa Biosystems, Massachusetts, United States

17 MagBio, Gaithersburg, Maryland, United States

18 Illumina, San Diego, United States

19 ThermoFisher Scientific

20 ThermoFisher Scientific

21 PerkinElmer, Waltham, Massachusetts, United States
