# Supplementary material for: Transcriptomic Profiling of JEG-3 cells using human leiomyoma derived matrix
Source: Biomater Biosyst. 2022 Jun 17;7:100056. doi: 10.1016/j.bbiosy.2022.100056 (PMC9934486; doi:10.1016/j.bbiosy.2022.100056)
Supplement: Supplementary Data S1 — Supplementary Raw Research Data. This is open data under the CC BY license http://creativecommons.org/licenses/by/4.0/ [file mmc1.pdf]

Transcriptomic Profiling of JEG-3 Cells Using Human Leiomyoma Derived Matrix

Samineh Barmaki*

Department of Pharmacology, Faculty of Medicine, University of Helsinki, 00290 Helsinki, Finland. E-Mail: samineh.barmaki@helsinki.fi

Ahmed Al-Samadi[1]

Department of Oral and Maxillofacial Disease, University of Helsinki, 00290 Helsinki, Finland

Katarzyna Leskinen[1]

Translational Immunology Research Program, and Department of Clinical and Medical Genetics, University of Helsinki, 00290 Helsinki, Finland

Wafa Wahbi

Department of Oral and Maxillofacial Disease, University of Helsinki, 00290 Helsinki, Finland

Ville Jokinen

Department of Chemistry and Materials Science, School of Chemical Engineering, Aalto University, 00076 Espoo, Finland

Sanna Vuoristo

Obstetrics and Gynecology, University of Helsinki and Helsinki University Hospital, 00290 Helsinki, Finland

Tuula Salo

Department of Oral and Maxillofacial Disease, University of Helsinki, 00290 Helsinki, Finland

Juha Kere

Department of Biosciences and Nutrition, Karolinska Institutet, 14183 Huddinge, Sweden Folkhälsan Research Center, 00290 Helsinki, Finland. Stem Cells and Metabolism Research Program, University of Helsinki, 00014 Helsinki,Finland

Satu Wedenoja[2]

Obstetrics and Gynecology, University of Helsinki and Helsinki University Hospital, 00290

*Corresponding author
[1]These authors contributed equally to this work.
[2]These authors contributed equally to this work.

Helsinki, Finland. Stem Cells and Metabolism Research Program, University of Helsinki, and Folkhälsan Research Center,00290 Helsinki,Finland

Päivi Saavalainen[2,*]

Translational Immunology Research Program, and Department of Clinical and Medical Genetics, University of Helsinki, 00290 Helsinki, Finland. Folkhälsan Research Center, 00290 Helsinki, Finland. E-Mail : paivi.saavalainen@helsinki.fi

---

Abstract

Oxygen tension varies during placental and fetal development. Although hypoxia drives early trophoblast invasion, low placental oxygen levels during pregnancy show association with pregnancy complications including fetal growth restriction and preeclampsia. JEG-3 cells are often used as a trophoblast model. We studied transcriptional changes of JEG-3 cells on a uterine leiomyoma derived matrix Myogel. This might be the closest condition to the real uterine environment that we can get for an in vitro model. We observed that culturing JEG-3 cells on the leiomyoma matrix leads to strong stimulation of ribosomal pathways, energy metabolism, and ATP production. Furthermore, Myogel improved JEG-3 cell adherence in comparison to tissue culture treated plastic. We also included PDMS microchip hypoxia creation, and observed changes in oxidative phosphorylation, oxygen related genes and several hypoxia genes. Our study highlights the effects of Myogel matrix on growing JEG-3 cells, especially on mitochondria, energy metabolism, and protein synthesis.

Keywords: Pre-eclampsia, Myogel, Hypoxia, Microfluidic, PDMS, Oxygen scavenger, JEG-3, Placenta, Microenvironment, Biomaterials

---

1. Introduction

Trophoblasts are temporary cells that form the epithelial layer of the placenta. Deep invasion of fetal trophoblasts into the uterine wall of the mother is a crucial step of the placentation to deliver adequate blood and oxygen supply

to the fetus. Defects in the trophoblast invasion during early pregnancy limit the placental development and capacity and might underlie a spectrum of human pregnancy complications, from miscarriages to fetal growth restriction and preeclampsia [1].

Many problems in human pregnancy show association with chronic placental hypoxia. Early stages of placentation are also accompanied with a decline in the oxygen levels at the maternal-fetal interface. This physiological hypoxia results in deeper invasion of extravillous trophoblasts into the wall of the uterus in search for oxygen, and formation and remodeling of the spiral arteries. In later stages of human pregnancy, however, trophoblasts are unable to invade and placental hypoxia leads to inflammation, oxidative stress, and apoptosis [2, 3, 4, 5, 6, 7, 8, 9].

Studying the earliest stages of placentation remains challenging. There are several reason for it. First, primary trophoblasts isolated from human first-trimester placentas do not proliferate in vitro [10]. Second, different hypoxia systems such as low-oxygen chambers and chemicals provide partly inconsistent findings [11]; and finally, the medium used in hypoxia experiments may further modify cellular responses [12]. To date, many studies have utilized JEG-3 chorioncarcinoma cell line as a model for early trophoblasts[10, 13, 14, 15].

Hypoxia-induced changes in JEG-3 cells involve reduction of the major placental hormone human chorionic gonadotropin (hCG), upregulation of the vascular endothelial growth factor (VEGF) [8, 10], and transcriptional regulation of a number of other genes by Hypoxia Inducible Factors (HIFs) [16]. However, results vary depending on the applied hypoxia system [17, 18, 19, 20, 21].

We have previously generated a microfluidic chip setup, where hypoxia is generated by pumping oxygen scavenger solution into a microfluidic channel that is separated from the cell culture by a gas-permeable membrane [22, 23].

We hypothesized that this system could be used to model hypoxia-induced gene expression changes of human trophoblast.

We studied RNA expression of JEG-3 cells using the hypoxia microchip we developed, and human leiomyoma- derived matrix Myogel, which mimics tumor

micro-environment and the human uterine wall [24, 25]. Also, we present cellular responses and the genes that are differentially expressed during hypoxia, and the utility of Myogel as an extracellular matrix for the JEG-3 culture.

2. Materials and Methods

2.1. Cell Culture

All experiments were performed using JEG-3 cells (RRID: CVCL-0363). These cells were cultured using Eagle's minimum essential medium [3] with non-essential amino acids, 90% sodium pyruvate, and 10% antibiotic-free Fetal bovine serum (FBS, Gibco). Cells from passages 5-12 were used for experiments. All cell culture experiments were performed using a regular cell incubator (37° with 5% CO_2).

2.2. Testing different coating on JEG-3 cells

24-well plates [4] were coated with poly-dimethyl-siloxane (PDMS). PDMS consists of two parts of polymer (Base Elastomer and Curing Agent). We used the standard mixing ration for PDMS, 10 parts base elastomer and 1 part curing agent (10 : 1) to coat the cell culture well plates. Then $150\mu l$ of PDMS added to the 24 well plates (surface area: $1.9cm^2$). We used the same PDMS ratio (10 : 1) to fabricate our previous reported microchips and the PDMS membrane).

Then, 0.5 mg/ml Myogel (50µl/well) and/or fibrin matrix were added on PDMS, while control wells remained uncoated. For stabilizing the coating, the plates were incubated in 37° with 5% CO_2 for 24 hours. JEG-3 cells were seeded 24 hours after coating of the plates. The cells were lysed after the first and third day for RNA analysis. All experiments were performed in three technical replicates.

We collected the cells on day 1 and day 3 to evaluate RNA concentrations. Because of the observed increase in RNA yields from day 1 to 3, and for the

[3] MEM, Gibco, Thermo Fisher Scientific Inc, Waltham, MA

[4] Thermo Fisher Scientific Inc, Waltham, MA

normal morphology of cells on day 1, we then performed RNA analyses on day 1 cells. Moreover, for the hypoxia experiment, we used an incubation time of 24 hours (day 1), which has been used in several earlier hypoxia studies on JEG-3 cells [9, 26].

2.3. PDMS microchip fabrication

The PDMS chips were fabricated by replication molding from SU-8 masters. The fabrication method and design of the microfluidic chips have been described previously [22]. Briefly: The SU-8[5] was spin coated 4000 rpm 30s for a $40\mu m$ thick layer. The chips were made from PDMS[6] with the ratio of 10:1 with the cross-linking agent. The chips were closed by bonding a $30\mu m$ thick PDMS membrane on top of the channels. The PDMS membrane was fabricated by spin coating PDMS $2000rpm$ $30s$ on top of an anti-adhesive fluoropolymer coats silicon wafer.

2.4. Microchip assay

The surface adherence plays essential role in PDMS attachment on PDMS reservoir [27]. The microfluidic chips and cell reservoir were washed gently with soap and rinsed with deionized water and 70% ethanol. Then, they were dried with pressurized air; a piece of scotch tape was used to remove any residual dust or particles from the surface. For testing the functionality of microfluidics before cell culture experiments, water was pumped with a microfluidic syringe pump [7] with flow rate of $0.1ml/h$ ($1.6\mu l/min$) into the microfluidic channels. If any leaks was detected during the test the chip is discarded.

A 2D cell culture matrix with Myogel was generated on PDMS membrane of microfluidic chip. The oxygen scavenger solution was pumped for $24hours$ in the two meanders of the microchip with the flow rate of $0.1ml/h$. Hypoxia was

[5]SU-8 50, Kayaku Advanced Materials, Westborough, MA
[6]Sylgard 184, Merck, KGaA, Darmstadt, Germany
[7]NE-4000, New Era Pump Systems Inc., Farmingdale, NY

induced on the cells that were separated from an underlying oxygen scavenging channel network by a thin PDMS membrane, as described previously [22, 23].

For making oxygen scavenger solution a water solution of sodium sulfite ($835 mM$ oxygen-depleted H_2O) was used to generate the oxygen sink. The reaction of sodium sulphite (Na_2SO_3) with dissolved oxygen in H_2O is utilized for oxygen depletion and generation of an active in-liquid oxygen sink. Cobalt nitrate ($Co\,(NO_3)_2$ hexahydrate salt, $9 mM$) in nitric acid was used as the catalyst. All reagents were obtained from Sigma Aldrich [8]. Figure 1 illustrates the structure of the hypoxia microchip, where hypoxia was generated by pumping of the oxygen scavenger solution in the microfluidic channels under the JEG-3 cells.

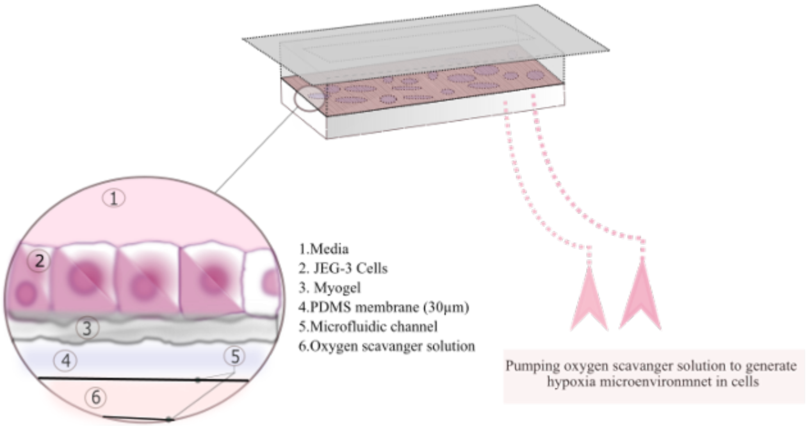

Figure 1: Structure of the hypoxia microchip.

2.5. RNA-sequencing and data analysis

RNA was extracted from the JEG-3 cells using the RNA easy Mini Kit [9] according to the manufacturer's instructions. Cells were homogenized with

[8]St. Louis, MA

[9]Qiagen, Hilden, Germany

QIAshredder columns (Qiagen). After extraction, RNA was quantified using Qubit 2 Fluorometer [10] with the Qubit RNA HS Assay Kit [11]. RNA samples were stored at $-70°C$. The RNA sequencing method was designed based on the Drop-seq. protocol, as described [20]. Briefly speaking, $10ng$ of RNA was mixed with Indexing Oligonucleotides [12]. After 5 minutes of incubation at ambient temperature, RNA was combined with RT mix containing $1\times$ Maxima RT buffer, $1mM$ dNTPs, $10U/\mu l$ Maxima HRTase, $1U/\mu l$ RiboLock [13], and $2.5\mu M$ Template Switch Oligo [14]. Samples were incubated in a T100 thermal cycler [15] for 30 minutes at $22°C$ and 90 minutes at $52°C$. The constructed cDNA was amplified by PCR in a volume of $15\mu l$ using $5\mu l$ of RT mix as template, $1\times$ HiFi HotStart Ready-mix [16], and $0.8\mu M$ SMART PCR primer. The samples were thermo-cycled in a T100 thermocycler as follows: $95°C$ 3 min; subsequently four cycles of $98°C$ for 20 sec, $65°C$ for 45 sec, $72°C$ for 3 min; following 16 cycles of $98°C$ for 20 sec, $67°C$ for 20 sec, $72°C$ for 3 min; and with the final extension step of 5 min at $72°C$. The PCR products were pooled together in sets of 27 samples containing different Indexing Oligos and purified with 0.6X High-Prep PCR Clean-up System [17] according to the manufacturer's instructions. They were eluted in $10\mu l$ of molecular grade water. The 3'-end cDNA fragments for sequencing were prepared using the Nextera XT [18] tagmentation reaction with $1ng$ of each PCR product serving as an input. The reaction was performed according to the manufacturer's instructions, with the exception that the P5 SMART primer was used instead of the i5 Nextera primer. After the PCR reaction, each set of samples was pooled and tagmented with a different Nextera i7 index. Subsequently, the samples were PCR amplified

[10]ThermoFisher Scientific
[11]ThermoFisher Scientific
[12]Integrated DNA Technologies
[13]ThermoFisher Scientific
[14]TSO, Integrated DNA Technologies
[15]BioRad, Hercules, California, United States
[16]Kapa Biosystems, Massachusetts, United States
[17]MagBio, Gaithersburg, Maryland, United States
[18]Illumina, San Diego, United States

as follows: 95°C for 30 sec; 11 cycles of 95°C for 10 sec, 55°C for 30 sec, 72°C for 30 sec; with the final extension step of 5 min at 72°C. Samples were purified twice using 0.6× and 1.0× High-Prep PCR Clean-up System (MagBio) and eluted in 10μl of molecular grade water.

The concentration of the library was measured using a Qubit 2 Fluorometer [19] and the Qubit DNA HS Assay Kit [20], while the quality was assessed using the LabChip GXII Touch HT electrophoresis system [21], with the DNA High Sensitivity Assay (PerkinElmer) and the DNA 5K/RNA/Charge Variant Assay LabChip (PerkinElmer). Samples were stored at $-20°C$. The libraries were sequenced on an Illumina NextSeq 500, with an addition of the custom primer producing a read 1 of 20bp and a read 2 (paired end) of 55bp. Sequencing was performed at the Functional Genomics Unit of the University of Helsinki. The RNA sequencing data has been deposited to NCBI Gene Expression Omnibus (Accession ID: GSE182988).

Raw sequence data was filtered to remove reads shorter than 20bp. Subsequently, the original pipeline for processing of Drop-seq data was used [28]. Briefly, reads were additionally filtered to remove poly A tails of length 6 or greater, and aligned to the human (GRCh38) genome using STAR aligner with default settings [29]. Uniquely mapped reads were grouped according to the barcodes 1 to 9, and gene transcripts were counted by their Unique Molecular Identifiers (UMIs) to reduce bias emerging from the PCR amplification. Digital expression matrices (DGE) were used for reporting the number of transcripts per gene in each sample (according to the distinct UMI sequences counted). Differentially expressed genes were identified using DESeq2 [30] with the cut-off for the adjusted p-value set to 0.05. We compared our hypoxia microchip data on JEG-3 RNA expression with an independent publicly available dataset. RNA-seq data on expression of JEG-3 cells cultured in a hypoxia chamber for 24 hours

[19]ThermoFisher Scientific
[20]ThermoFisher Scientific
[21]PerkinElmer, Waltham, Massachusetts, United States

[31] were downloaded from the GEO under the accession number GSM1862652.

Normalization and differential expression analysis between these two data sets were performed using edgeR [32] package integrated into Chipster platform [33]. Heatmaps were drawn with heatmapper tool [34] and pathway analyses were performed with Enrichr (MSigDB Hallmark 2020 pathway) [35, 36].

3. Results

3.1. Influence of Myogel coating on gene expression and cell morphology

We assessed the morphology and RNA expression of JEG-3 cells at the first day of the cell culture. We compared cell morphology and analyzed gene expression of the JEG-3 cells cultured in the wells coated with Myogel, PDMS, Myogel + PDMS, Myogel + fibrin, Myogel + fibrin + PDMS, and uncoated control, (Figure 3). Total RNA yields increased in extractions from day one to day 3 (Supplementary Table S10), indicating that the cells were proliferating. The genes and corresponding pathways modulated by different conditions were studied. Some of the most significant findings were in association with Myogel coating, (Table 1 ; Figure 2). When Myogel was added to the JEG-3 culture, 308 genes showed differential expression (Supplementary Table S2), and Myogel provided better cell adhesion (Figure 3c).

The gene expression changes that were observed were related to several neurological diseases (Huntington disease, Parkinson disease, and Alzheimer disease). This might reflect the crucial role of oxygen supply in different tissues, including both the placenta and brain . This finding indicates that the changes we observed were not trophoblast specific but more related to general changes in energy metabolism. These pathways were tightly related to several NDUF genes (NDUFB7; NDUFB3; NDUFB; NDUFV1) mitochondrial respiratory genes, and ribosomal protein metabolism. We also assessed how PDMS and fibrin - or their combinations - modulate gene expression when they were used with Myogel. When the cells were coated with the combination of Myogel, fibrin, and PDMS, we observed significant modulation of ribosomal genes. This observation was

similar to what has been seen with pure Myogel; accordingly, fibrin itself has very small effects on gene expression, (Table 1 , Figure 2). This was particularly demonstrated between the conditions PDMS + Myogel vs. PDMS + fibrin + Myogel, showing almost no changes in gene expression. However, the comparison between Myogel + fibrin + PDMS vs. PDMS only produced significant changes in gene expression (Table 1, Figure 2 and Figure 3).

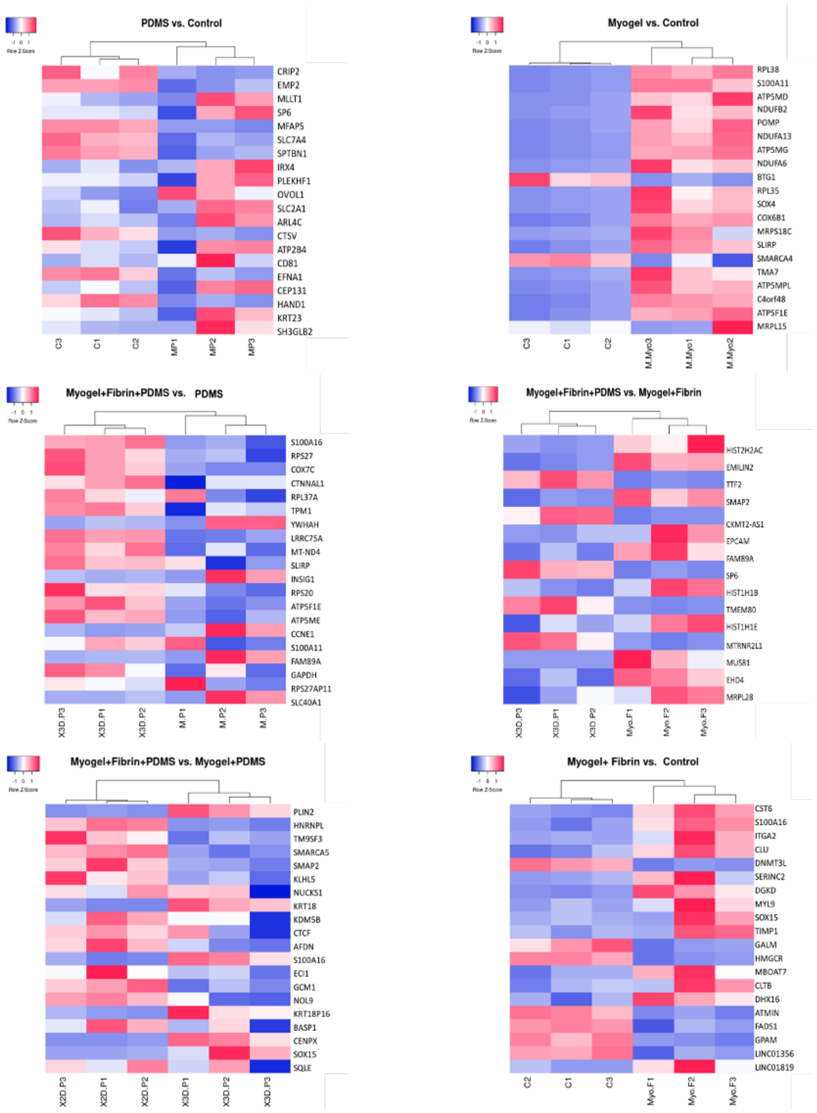

Figure 2: Comparison of differentially expressed genes between the well plates coated with PDMS, Myogel, fibrin, and their combinations. Heat-maps show the top 20 differentially expressed genes (with 2-fold change or greater) in JEG-3 cells for each conditions after hierarchical clustering analysis. Each column represents three replicates for each of the two conditions (M.P: PDMS, C: Control, Myo.F: Myogel + Fibrin (Myo: Myogel, F: Fibrin), X3D.P: Myogel + Fibrin + PDMS,(X3D: Myogel+Fibrin, P: PDMS) X2D.P: Myogel + PDMS (X2D:Myogel,P: PDMS), M.Myo : Myogel), and the rows indicate the genes. The gene expression levels were shown by color and intensity: red indicates down-regulation and blue upregulation, and higher color intensity reflects higher fold change. Statistical analysis done by heatmapper, Three replicates from each condition, (n=3).

Although the Myogel culture-associated pathways indicated significant changes, single genes were not changed to the same extent. Analysis of differentially expressed genes revealed upregulation of hypoxia pathway with two genes associated with PDMS coating vs. control: EFNA1 and SLC2A1 (P-value: 0.016), (see Figure 2). This may be caused by hydrophobic surface property of PDMS that induces organoid type of structures (Figure 3b), suggesting that oxygen might not diffuse freely in the cells. However, hypoxic pathway was also up-regulated by Myogel vs. pure medium, as seen about the genes EFNA1, SLC25A1, BTG1, ANXA2, and CAV1 (P-value: 0.196). Interestingly, adding Myogel on the PDMS coated culture prevented the development of organoid structures (see Figure 3d), but showed no clear changes in gene expression. In contrast, adding fibrin to Myogel-coated culture resulted in no changes in gene expression, but three dimensional organization of the cells was observed (Figure3e).

Detailed information of well-plate experiment, such as DE-gene lists (Supplementary Tables S3-S9) and significant genes in one heat-map (see Supplementary Figure S3) with Venn diagram of important pathways of well-plate experiment (Supplementary Figure S4) showed in the supplementary section.

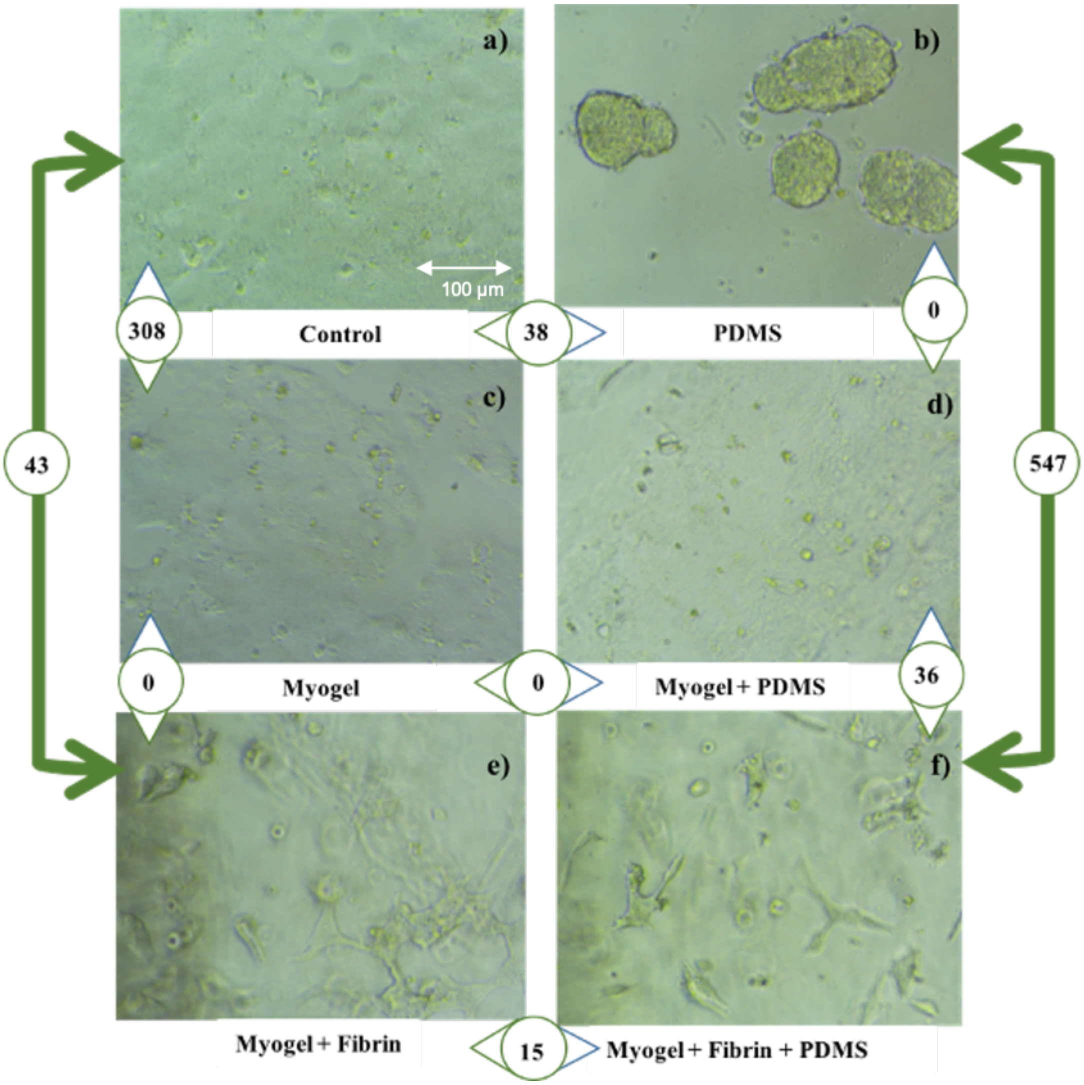

Figure 3: Morphology and the number of significantly differentially expressed genes in JEG-3 cells in the cultures a) Control (only medium),308 significant gene expressed in Myogel over control, 38 genes expressed in PDMS over control, 43 genes expressed in Myogel + Fibrin over control. b) PDMS,not significant genes expressed between PDMS and Myogel + PDMS, 547 genes expressed in Myogel+Fibrin+PDMS over PDMS. c) Myogel,not significant genes expressed in Myogel + Fibrin over Myogel and Myogel + PDMS over Myogel. d) Myogel + PDMS,36 genes expressed in in Myogel+Fibrin+PDMS over Myogel + PDMS. e) Myogel + fibrin,15 genes expressed in Myogel + Fibrin + PDMS over Myogel + Fibrin. f) Myogel + fibrin + PDMS. Three replicates from each condition (n=3).

**PDMS *v.s.* Control**			**Myogel *v.s.* Control**		
**Term**	**P-value**	**Genes**	**Term**	**P-value**	**Genes**
Coagulation	0.008	CRIP2; CTSV	Oxidative Phosphorylation	≈ 0	COX7B; SLC25A3; ECHS1; NDUFB7; NDUFB3; NDUFB; NDUFV1; ATP5G; ATP5ME; COX8A; TIMM8B; ATP5P
Mitotic Spindle	0.016	CEP131; SPTBN1	Myc Targets V1	≈ 03	DDX18; SLC25A3; CNBP; RPLP0; ILF2; SRPK1; LSM2; PSMD8; CDC20; AIMP2; SYNCRIP; FBL; LSM7; C1QBP; NDUFAB1; POLD2; RACK1; SRSF2; SNRPG; SRSF7; EIF4G2
Hypoxia	0.016	EFNA1; SLC2A1	Adipogenesis	≈ 0	COX8A; SLC25A1; COX7B; MTCH2; ECHS1; NDUFB7; ITSN1; ECH1; TALDO1; CHCHD10; UQCR11; ATP1B3; MRPL15; UQCR10; UQCRQ; NDUFAB1; UQCRC1; SUCLG1
Estrogen Response Late	0.016	ATP2B4; EMP2	E2F Targets	0.001	CDC20; SYNCRIP; ILF3; POP7; POLD2; SRSF2; PSIP1; MKI67; DCTPP1; RAN
TGF-beta Signaling	0.052	SPTBN1	Peroxisome	0.001	FDPS; VPS4B; CNBP; ECH1; SLC25A4; FADS1; YWHAH
PI3K / AKT / mTOR Signaling	0.099	SLC2A1	Myc Targets V2	0.001	DDX18; AIMP2; PPAN; FARSA; DCTPP1
UV Response Dn	0.134	ATP2B4	Fatty Acid Metabolism	0.003	ECHS1; NTHL1; UROS; ECH1; SUCLG1; PRDX6; HSD17B10; YWHAH
UV Response Up	0.146	CTSV	G2-M Checkpoint	0.003	CDC20; TPX2; SYNCRIP; PRMT5; GINS2; ILF3; UBE2C; SRSF2; MKI67
IL-2 / STAT5 Signaling	0.181	CD81	Cholesterol Homeostasis	0.005	FDPS;EBP;ECH1; HMGCR; S100A11
TNF-alpha Signaling via NF-kB	0.182	EFNA1	Unfolded Protein Response	0.008	RPS14; NOP14; EXOSC4; IMP3; POP4; HSP90B1
p53 Pathway	0.182	CD81	DNA Repair	0.028	NT5C; MPG; ELOA; SAC3D1; ITPA; POLR2K
heme Metabolism	0.182	SLC2A1	Androgen Response	0.068	KRT19; UBE2I; HMGCR; FADS1
Estrogen Response Early	0.182	SLC2A1	Estrogen Response Late	0.09	CDC20; GINS2; KRT19; PERP; CAV1; DCXR
Complement	0.182	CTSV	mTORC1 Signaling	0.09	CCT6A; EBP; HMGCR; FADS1; HSP90B1; ATP5MC1
mTORC1 Signaling	0.1821	SLC2A1	p53 Pathway	0.09	LDHB; BTG1; PERP; DCXR; RGS16; RACK1
Epithelial Mesenchymal Transition	0.182	MFAP5	UV Response Up	0.09	NFKBIA; EIF5; BTG1; EPCAM; SLC25A4
			Reactive Oxygen Species Pathway	0.17	NDUFA6; PRDX6
			Protein Secretion	0.18	SGMS1; VPS4B;CLTA
			Mitotic Spindle	0.193	TPX2; PPP4R2; ITSN1; CKAP5; SAC3D1
			Hypoxia	0.196	EFNA1; SLC25A1; BTG1; ANXA2; CAV1

Table 1: Up-regulated Pathways of JEG cells on well plate experiment

3.2. Genes and pathways in Myogel coated hypoxia microchip

The data suggested that Myogel enhances cell adherence on PDMS, without inducing changes in gene expression. The cell adhesion was visually suspected because they were tightly adherent, when collected for RNA extraction. These results prompted us to use Myogel coating on PDMS microchips in hypoxia experiments. We assessed the genes and pathways that were modulated in Myogel coated PDMS microchips and exposed to oxygen scavenger for 24 hours. The chip generates oxygen levels of $< 5\%$, as shown in our previous study , through the activation of image-iT® red fluorescent marker. Because of the formation of organoid structures , we could not use pure PDMS as a control in these experiments, (see Figure 3 *a* and *b*). We used Myogel on PDMS microchip, as we observed that PDMS had no effect on gene expression when it was used in addition to Myogel, (Figure 3 *c* and *d*). Moreover, liquid form of pure Myogel, in contrast with respect to the thick gel formed with fibrin, allowed the cells to adhere on the PDMS membrane as a single layer, exposing the cells evenly to hypoxia.

In normoxia, the cells were cultured on a microchip pumped with water , whereas in hypoxia, the microchip was pumped with oxygen scavenger solution for 24 hours. Principal component analysis (PCA) of samples cultured under hypoxia or normoxia samples microchips revealed a clear separation between the samples, as expected (Supplementary Figure S1).

4. Discussion

Ribosome biosynthesis, the top pathway modulated by JEG-3 cells cultured on Myogel, plays a role in protein synthesis, cell growth, and tumorigenesis [35]. Our findings indicate significant metabolic changes when Myogel was used as a matrix of a JEG-3 cell culture. These data suggest that Myogel promotes JEG-3 cells growth and differentiation, and regulates pathways that are associated with human diseases.

	Our study		Zhu et al.		MSigDB pathways involved (Enrichr)							
DE genes in both sets	log2 FC	p(adj)	log2 FC	p(adj)	Hypoxia	mTORC1 Signaling	Myc Targets V1	p53 Pathway	Apoptosis	Glycolysis	E2F Targets	G2-M Checkpoint
RPS27	1.24	5.15E-08	1.01	3.33E-02								
FTH1	1.10	6.86E-05	1.43	2.85E-04								
RPS24	0.93	2.65E-04	1.03	2.54E-02								
CANX	1.00	4.43E-04	1.56	1.07E-02		x	x					
RPS27A	0.76	2.04E-03	1.33	1.70E-02								
GAPDH	0.84	2.22E-03	1.85	7.22E-07	x	x					x	
HMGB2	0.76	5.35E-03	3.63	3.82E-04					x		x	
JUND	0.71	1.14E-02	1.82	1.89E-03								
SFPQ	0.65	1.22E-02	2.32	2.26E-03								x
CCSAP	0.63	1.87E-02	1.90	1.46E-02								
GM2A	0.56	2.88E-02	1.52	2.07E-03				x				
ENO1	0.52	3.06E-02	4.22	3.98E-14	x	x				x		
RACK1	0.59	3.85E-02	1.01	3.66E-02			x					
TPI1	0.57	4.59E-02	1.29	6.59E-03	x	x				x		

Table 2: 14 genes that were differentially expressed both in our microchip experiment and in the dataset of Zhu et al. Log2 fold changes and adjusted P-values are compared, and known pathways involved are shown.

Adding PDMS on Myogel + fibrin coating showed no obvious changes in cell morphology and no organoid structures, but 36 genes were up-regulated. Collectively, these morphological findings are in line with the previous observations, showing that Myogel provides better cell migration, and Myogel + fibrin coating generates two and three dimensional matrices [24, 25, 36]. Considered together, these data indicate that the coating has a marked impact on gene expression and morphology, including key cellular processes such as ribosome biosynthesis (mainly Myogel related effect), endocytosis, and adhesion-associated genes such as several of the matrix metalloproteases [37, 38]. The main pathways that were repressed by hypoxia microchip included mTOR (Mammalian target of rapamycin), MYC (Myelocytomatosis oncogene), E2F (Early 2 factor), and oxidative phosphorylation. Because MYC is a major cell proliferation marker,

upregulation of MYC together with G2M checkpoint support that the cells start to proliferate faster in response to hypoxia, see Supplementary Figure S2. These findings point toward reprogramming of cancer cell metabolism, tumor cell growth and progression, and mitochondrial metabolism [39, 40, 41, 42, 43, 38].

We observed altogether 306 differentially expressed genes in JEG-3 cells in hypoxia vs. normoxia (Supplementary Table S1), including upregulation of the mitochondrially encoded genes such as MT-RNR2, MT-ND4, MT-CO2, and MT-CYB, and the lncRNA gene MALAT1. These genes play key roles in mitochondria metabolism, which might reflect the effects of the limited oxygen availability [44]. The most significantly down-regulated genes during hypoxia included UQCR11, IP6K1, STK17A, NFE2L2, and C1QBP. These genes activated oxidative stress, MYC targets (v1,v2), and TNF alpha/NF-kB, all of which modulate cell proliferation, differentiation, apoptosis and coagulation [45, 46, 47, 48, 49, 50, 51].

Although the pathways associated with cell proliferation were activated in response to oxygen scavenger, we found no significant enrichment of the hypoxia pathway genes in the Enrichr pathway analysis. However, five individual hypoxia related genes showed significant upregulation: AKAP12, TPI1, ZNF292, ENO1, and GAPDH. We further compared our genes and pathways modulated in the hypoxia microchip experiment by reanalyzing the results of Zhu et al. [52]. They have studied the effect of hypoxia using JEG-3 cells in a hypoxia chamber for 16 hours. In their experiment, 131 genes were differentially expressed. Of these genes, 14 were differentially expressed also on our microchip, and 12 of these showed effects to the same direction, although the expression fold changes were smaller with our microchip (see Table 2). The most important pathways that were linked to differentially express genes in both our study and by Zhu et al. were oxidative phosphorylation, E2F targets, P53 pathways, apoptosis, hypoxia, and PI3K/AKT/mTOR signaling. Altogether, our study found genes from 33 shared pathways that were observed in the study of Zhu et al.,see Supplementary Table S2. Notably, MYC pathway was similarly enriched in both studies. Although only few genes from hypoxia pathway were observed in our

microchip experiment, we observed changes in the expression of mitochondrial genes (MT-RNR2, MT-ND4, MT-CO2, and MT-CYB). This could be related to oxidative stress and reflect the cellular environment in our experiment.

The non-significant effects of our microchip on hypoxia pathway might be related to Myogel, microchip setup, or the duration and the level of hypoxia. In contrast with hypoxic chambers, our microchip involves oxygen deprivation from the apical surface of the cells, which is in contact with the PDMS membrane. It is therefore possible that the luminal side of the cells gets more oxygen from the culture media, and results in only a modest change of hypoxia induced gene expression. The other possibility for the insufficient hypoxia response on our microchip setup could be the chemical reactions of the oxygen scavenger solution. Hypoxic condition are generated by the conversion of sodium sulphite to sodium sulphate (equation 1).[53]. Furthermore, sodium sulphite generates sulphur dioxide (SO_2) by reacting with water (equation 2).[54]. Since PDMS is gas permeable , SO_2 could cross PDMS membrane and influence the significance of generation of hypoxia on the microchip system.

Equation 1. $2Na_2SO_3 + O_2 \longrightarrow 2Na_2SO_4$

Equation 2. $Na_2SO_3 + H_2O \longrightarrow SO_2 + 2NaOH$

Although we were unable to measure the exact level of hypoxia on the microchips along with RNA analyses, our previous studies support low oxygen levels ($O_2 \leq 5\%$) produced by the microchip [22, 23].

It also remains possible that Myogel prevented or modulated the expected effects of the oxygen scavenger. Remarkably, uterine myomas might be hypoxic as such [55], and this could further interfere with the effects of the hypoxia on Myogel coated microchips in comparison with the previous studies.

5. Conclusion

This study shows that JEG-3 cells cultured on the Myogel matrix associate with strong stimulation of ribosomal pathways, energy metabolism, and ATP production. Myogel matrix provides better JEG-3 cell adherence on PDMS and

shows significant effects on cell morphology and RNA expression. Although our results did not show significant upregulation of the hypoxia pathway genes using the oxygen scavenger and the microchip setup, significant upregulation of several individual hypoxia-related genes and the oxidative phosphorylation pathway arose. These results provide important insights into the possibility to modulate cellular processes and gene expression by different coating. Moreover, our results support the utility of cellular microenvironments in JEG-3 studies. Further studies are needed to assess the effects of temporary and chronic hypoxia in the cell morphology, invasive capacity, and gene expression.

Acknowledgements

The author would like to thank Dr.Esko Kankuri, Lahja Eurojoki, and Hanne Ahola for their laboratory assistance during the project.

Funding

This research was funded by University of Helsinki - Doctoral program of Health science .

References

[1] M. J. Soares, K. M. Varberg, K. Iqbal, Hemochorial placentation: development, function, and adaptations†, Biology of Reproduction 99 (1) (2018) 196–211. arXiv:https://academic.oup.com/biolreprod/article-pdf/99/1/196/25155045/ioy049.pdf, doi:10.1093/biolre/ioy049. URL https://doi.org/10.1093/biolre/ioy049

[2] G. L. Semenza, Oxygen homeostasis, WIREs Systems Biology and Medicine 2 (3) (2010) 336–361. arXiv:https://wires.onlinelibrary.wiley.com/doi/pdf/10.1002/wsbm.69, doi:https://doi.org/10.1002/wsbm.69. URL https://wires.onlinelibrary.wiley.com/doi/abs/10.1002/wsbm.69

[3] X.-Q. Hu, L. Zhang, Hypoxia and mitochondrial dysfunction in pregnancy complications, Antioxidants 10 (3). doi:10.3390/antiox10030405. URL https://www.mdpi.com/2076-3921/10/3/405

[4] G. J. Burton, Oxygen, the Janus gas; its effects on human placental development and function, J Anat 215 (1) (2009) 27–35.

[5] R. Tal, A. Shaish, I. Barshack, S. Polak-Charcon, A. Afek, A. Volkov, B. Feldman, C. Avivi, D. Harats, Effects of hypoxia-inducible factor-1alpha overexpression in pregnant mice: possible implications for preeclampsia and intrauterine growth restriction, Am J Pathol 177 (6) (2010) 2950–2962.

[6] M. Soares, K. Iqbal, K. Kozai, Hypoxia and placental development, Birth Defects Research 109 (2017) 1309–1329. doi:10.1002/bdr2.1135.

[7] N. Soleymanlou, I. Jurisica, O. Nevo, F. Ietta, X. Zhang, S. Zamudio, M. Post, I. Caniggia, Molecular evidence of placental hypoxia in preeclampsia, J Clin Endocrinol Metab 90 (7) (2005) 4299–4308.

[8] S. J. Fisher, Why is placentation abnormal in preeclampsia?, Am J Obstet Gynecol 213 (4 Suppl) (2015) S115–122.

[9] M. Garziera, L. Scarabel, G. Toffoli, Hypoxic Modulation of HLA-G Expression through the Metabolic Sensor HIF-1 in Human Cancer Cells, J Immunol Res 2017 (2017) 4587520.

[10] C. Q. Lee, L. Gardner, M. Turco, N. Zhao, M. J. Murray, N. Coleman, J. Rossant, M. Hemberger, A. Moffett, What Is Trophoblast? A Combination of Criteria Define Human First-Trimester Trophoblast, Stem Cell Reports 6 (2) (2016) 257–272.

[11] M. Byrne, M. Leslie, R. Gaskins, P. Kenis, Methods to study the tumor microenvironment under controlled oxygen conditions, Trends in biotechnology 32. doi:10.1016/j.tibtech.2014.09.006.

[12] M. Bilban, S. Tauber, P. Haslinger, J. Pollheimer, L. Saleh, H. Pehamberger, O. Wagner, M. Knöfler, Trophoblast invasion: assessment of cellular models using gene expression signatures, Placenta 31 (11) (2010) 989–996.

[13] X. C. . H. W. Qian Zhang, Zhenzhen Wang, lncrna dancr promotes the migration an invasion and of trophoblast cells through microrna-214-5p in preeclampsia., Bioengineered 12 (2021) 9424–9434. doi:10.1080/21655979.2021.1988373.

[14] V. Mikhailova, E. Khokhlova, P. Grebenkina, Z. Salloum, I. Nikolaenkov, K. Markova, A. Davidova, S. Selkov, D. Sokolov, Nk-92 cells change their phenotype and function when cocultured with il-15, il-18 and trophoblast cells., Immunobiology 226 (2021) 152125. doi:10.1016/j.imbio.2021.152125.

[15] J. M. Murrieta-Coxca, R. N. Gutiérrez-Samudio, H. M. El-Shorafa, T. Groten, S. Rodríguez-Martínez, M. E. Cancino-Diaz, J. C. Cancino-Diaz, R. R. Favaro, U. R. Markert, D. M. Morales-Prieto, Role of il-36 cytokines in the regulation of angiogenesis potential of trophoblast cells., International journal of molecular sciences 22 (2020) 285. doi:10.3390/ijms22010285.

[16] J. Patel, K. Landers, R. H. Mortimer, K. Richard, Regulation of hypoxia inducible factors (HIF) in hypoxia and normoxia during placental development, Placenta 31 (11) (2010) 951–957.

[17] H. Strohmer, H. Kiss, B. Mösl, C. Egarter, P. Husslein, M. Knöfler, Hypoxia downregulates continuous and interleukin-1-induced expression of human chorionic gonadotropin in choriocarcinoma cells, Placenta 18 (7) (1997) 597–604.

[18] C. M. Taylor, H. Stevens, F. W. Anthony, T. Wheeler, Influence of hypoxia on vascular endothelial growth factor and chorionic gonadotrophin production in the trophoblast-derived cell lines: JEG, JAr and BeWo, Placenta 18 (5-6).

[19] B. S. Shin, H. G. Kim, O. H. Choi, Mitochondrial Channel Opener Diazoxide Attenuates Hypoxia-Induced sFlt-1 Release in Human Choriocarcinoma Cells, J Menopausal Med 20 (1) (2014) 21–31.

[20] H. M. Qu, L. P. Qu, X. Y. Li, X. Z. Pan, Overexpressed HO-1 is associated with reduced STAT3 activation in preeclampsia placenta and inhibits STAT3 phosphorylation in placental JEG-3 cells under hypoxia, Arch Med Sci 14 (3) (2018) 597–607.

[21] J. Zhao, R. P. Chow, R. H. McLeese, M. B. Hookham, T. J. Lyons, J. Y. Yu, Modelling preeclampsia: a comparative analysis of the common human trophoblast cell lines, FASEB Bioadv 3 (1) (2021) 23–35.

[22] S. Barmaki, V. Jokinen, D. Obermaier, D. Blokhina, M. Korhonen, R. H. A. Ras, J. Vuola, S. Franssila, E. Kankuri, A microfluidic oxygen sink to create a targeted cellular hypoxic microenvironment under ambient atmospheric conditions, Acta Biomater 73 (2018) 167–179.

[23] S. Barmaki, D. Obermaier, E. Kankuri, J. Vuola, S. Franssila, V. Jokinen, A microfluidic chip architecture enabling a hypoxic microenvironment and nitric oxide delivery in cell culture, Micromachines 11 (11). `doi:10.3390/mi11110979`.
URL `https://www.mdpi.com/2072-666X/11/11/979`

[24] T. Salo, M. Sutinen, E. Hoque Apu, E. Sundquist, N. K. Cervigne, C. E. de Oliveira, S. U. Akram, S. Ohlmeier, F. Suomi, L. Eklund, P. Juusela, P. Åström, C. C. Bitu, M. Santala, K. Savolainen, J. Korvala, A. F. Paes Leme, R. D. Coletta, A novel human leiomyoma tissue derived matrix for cell culture studies, BMC Cancer 15 (2015) 981.

[25] T. Salo, M. R. Dourado, E. Sundquist, E. H. Apu, I. Alahuhta, K. Tuomainen, J. Vasara, A. Al-Samadi, Organotypic three-dimensional assays based on human leiomyoma-derived matrices, Philos Trans R Soc Lond B Biol Sci 373 (1737).

[26] H. Nishi, T. Nakada, M. Hokamura, Y. Osakabe, O. Itokazu, L. E. Huang, K. Isaka, Hypoxia-inducible factor-1 transactivates transforming growth factor-beta3 in trophoblast., Endocrinology (2004) 4113–4118.

[27] H. Sun, A. Zhao, M. Li, H. Dong, Y. Sun, X. Zhang, Q. Zhu, A. A. Bukhari, C. Cao, D. Su, Y. Liu, X. Liang, Interaction of calcium binding protein S100A16 with myosin-9 promotes cytoskeleton reorganization in renal tubulointerstitial fibrosis, Cell Death Dis 11 (2) (2020) 146.

[28] E. C. Hatchell, S. M. Colley, D. J. Beveridge, M. R. Epis, L. M. Stuart, K. M. Giles, A. D. Redfern, L. E. Miles, A. Barker, L. M. MacDonald, P. G. Arthur, J. C. Lui, J. L. Golding, R. K. McCulloch, C. B. Metcalf, J. A. Wilce, M. C. Wilce, R. B. Lanz, B. W. O'Malley, P. J. Leedman, SLIRP, a small SRA binding protein, is a nuclear receptor corepressor, Mol Cell 22 (5) (2006) 657–668.

[29] M. I. Love, W. Huber, S. Anders, Moderated estimation of fold change and dispersion for RNA-seq data with DESeq2, Genome Biol 15 (12) (2014) 550.

[30] T. Sasagawa, T. Nagamatsu, K. Morita, N. Mimura, T. Iriyama, T. Fujii, M. Shibuya, HIF-2α, but not HIF-1α, mediates hypoxia-induced upregulation of Flt-1 gene expression in placental trophoblasts, Sci Rep 8 (1) (2018) 17375.

[31] J. S. Kim, Y. S. Choi, J. H. Park, J. Yun, S. Kim, J. H. Lee, B. H. Yun, J. H. Park, S. K. Seo, S. Cho, H. S. Kim, B. S. Lee, Role of B-Cell Translocation Gene 1 in the Pathogenesis of Endometriosis, Int J Mol Sci 20 (13).

[32] M. D. Robinson, D. J. McCarthy, G. K. Smyth, edger:a bioconductor package for differential expression analysis of digital gene expression data., Bioinformatics 26 (2010) 139–140. doi:10.1093/bioinformatics/btp616.

[33] M. A. Kallio, J. T. Tuimala, T. Hupponen, P. Klemelä, M. Gentile, I. Scheinin, M. Koski, J. Käki, E. I. Korpelainen, Chipster: user-friendly

analysis software for microarray and other high-throughput data, BMC Genomics 12 (2011) 507.

[34] S. Babicki, D. Arndt, A. Marcu, Y. Liang, J. R. Grant, A. Maciejewski, D. S. Wishart, Heatmapper: web-enabled heat mapping for all., Nucleic Acids Res.doi:10.1093/nar/gkw419.

[35] E. Y. Chen, C. M. Tan, Y. Kou, Q. Duan, Z. Wang, G. V. Meirelles, N. R. Clark, A. Ma'ayan, Enrichr: interactive and collaborative HTML5 gene list enrichment analysis tool, BMC Bioinformatics 14 (2013) 128.

[36] W. Wahbi, E. Naakka, K. Tuomainen, I. Suleymanova, A. Arpalahti, I. Miinalainen, J. Vaananen, R. Grenman, O. Monni, A. Al-Samadi, T. Salo, The critical effects of matrices on cultured carcinoma cells: Human tumor-derived matrix promotes cell invasive properties, Exp Cell Res 389 (1) (2020) 111885.

[37] M. V. Kuleshov, M. R. Jones, A. D. Rouillard, N. F. Fernandez, Q. Duan, Z. Wang, S. Koplev, S. L. Jenkins, K. M. Jagodnik, A. Lachmann, M. G. McDermott, C. D. Monteiro, G. W. Gundersen, A. Ma'ayan, Enrichr: a comprehensive gene set enrichment analysis web server 2016 update, Nucleic Acids Res 44 (W1) (2016) W90–97.

[38] Y. Cui, L. Li, Z. Li, J. Yin, J. Lane, J. Ji, W. G. Jiang, Dual effects of targeting S100A11 on suppressing cellular metastatic properties and sensitizing drug response in gastric cancer, Cancer Cell Int 21 (1) (2021) 243.

[39] J. Berg, J. Tymoczko, L. Stryer, Biochemistry, Fifth Edition, W.H. Freeman, 2002.
URL https://books.google.se/books?id=uDFqAAAAMAAJ

[40] M. Blagosklonny, Hypoxia, mtor and autophagy, Autophagy 9. doi:10.4161/auto.22783.

[41] R. J. Duronio, Y. Xiong, Signaling pathways that control cell proliferation., Cold Spring Harbor perspectives in biology 5 3 (2013) a008904.

[42] W. Ding, X. L. Fan, X. Xu, J. Z. Huang, S. H. Xu, Q. Geng, R. Li, D. Chen, G. R. Yan, Epigenetic silencing of ITGA2 by MiR-373 promotes cell migration in breast cancer, PLoS One 10 (8) (2015) e0135128.

[43] J. Bassler, E. Hurt, Eukaryotic ribosome assembly, Annual Review of Biochemistry 88. `doi:10.1146/annurev-biochem-013118-110817`.

[44] I. Papandreou, R. A. Cairns, L. Fontana, A. L. Lim, N. C. Denko, HIF-1 mediates adaptation to hypoxia by actively downregulating mitochondrial oxygen consumption, Cell Metab 3 (3) (2006) 187–197.

[45] J. W. Kim, P. Gao, Y. C. Liu, G. L. Semenza, C. V. Dang, Hypoxia-inducible factor 1 and dysregulated c-Myc cooperatively induce vascular endothelial growth factor and metabolic switches hexokinase 2 and pyruvate dehydrogenase kinase 1, Mol Cell Biol 27 (21) (2007) 7381–7393.

[46] H. X. Sun, Y. Xu, X. R. Yang, W. M. Wang, H. Bai, R. Y. Shi, S. K. Nayar, R. P. Devbhandari, Y. Z. He, Q. F. Zhu, Y. F. Sun, B. Hu, M. Khan, R. A. Anders, J. Fan, Hypoxia inducible factor 2 alpha inhibits hepatocellular carcinoma growth through the transcription factor dimerization partner 3/ E2F transcription factor 1-dependent apoptotic pathway, Hepatology 57 (3) (2013) 1088–1097.

[47] M. Hubbi, G. Semenza, Regulation of cell proliferation by hypoxia-inducible factors, American journal of physiology. Cell physiology 309 (2015) ajpcell.00279.2015. `doi:10.1152/ajpcell.00279.2015`.

[48] I. Godet, Y. J. Shin, J. A. Ju, I. C. Ye, G. Wang, D. M. Gilkes, Fate-mapping post-hypoxic tumor cells reveals a ROS-resistant phenotype that promotes metastasis, Nat Commun 10 (1) (2019) 4862.

[49] A. Baluapuri, E. Wolf, M. Eilers, Target gene-independent functions of MYC oncoproteins, Nat Rev Mol Cell Biol 21 (5) (2020) 255–267.

[50] X. Q. Hu, L. Zhang, Hypoxia and Mitochondrial Dysfunction in Pregnancy Complications, Antioxidants (Basel) 10 (3).

[51] O. Holland, M. Dekker Nitert, L. A. Gallo, M. Vejzovic, J. J. Fisher, A. V. Perkins, Review: Placental mitochondrial function and structure in gestational disorders, Placenta 54 (2017) 2–9.

[52] R. Zhu, L; Lv, Effect of hypoxia in severe preeclampsia through epigenetic regulation, `https://www.ncbi.nlm.nih.gov/geo/query/acc.cgi?acc=GSE72437` (May 2017).

[53] K. Marino, E. Silva, J. Windelborn, A comparison between chemical and gas hypoxia as models of global ischemia in zebrafish (danio rerio), Animal Models and Experimental Medicine 3. `doi:10.1002/ame2.12132`.

[54] J. Bin, C. Ren, Y. Li, Y. Lu, W. Li, Y. Wu, Y. Gao, P. Ratcliffe, H. Liu, C. Zhang, Sodium sulfite is a potential hypoxia inducer that mimics hypoxic stress in caenorhabditis elegans, Journal of biological inorganic chemistry : JBIC : a publication of the Society of Biological Inorganic Chemistry 16 (2010) 267–74. `doi:10.1007/s00775-010-0723-1`.

[55] S. Teppo, E. Sundquist, M. Vered, H. Holappa, J. Parkkisenniemi, T. A. Rinaldi, P. Lehenkari, R. A. Grenman, D. Dayan, J. P. Risteli, T. Salo, P. Nyberg, The hypoxic tumor microenvironment regulates invasion of aggressive oral carcinoma cells., Experimental cell research 319 4 (2013) 376–89.

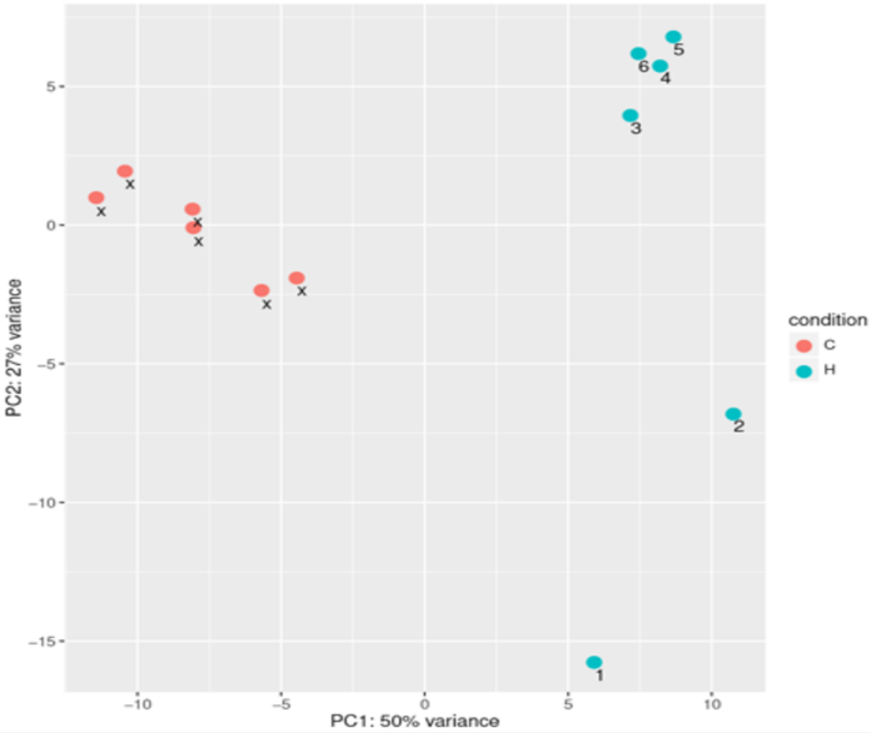

Supplementary Figure S1. Each column represents individual microchip (C1, C2: Samples from the control microchip, H1, H2: Samples from the hypoxia microchip). PCA Principle component PC1 (50%) separated all microchip samples and PC2 (27%) separated the hypoxia from control, The statistical analysis done by chipster [33].n=2

Pathway	P-value	Genes
**Myc Targets V1**	6.29E-15	NOP56; DDX18; SMARCC1; NPM1; SET; HSP90AB1; SSB; CBX3; PABPC4; RPS6; DDX21; NAP1L1; RPL6; NME1; HSPD1; RSL1D1; PSMD7; C1QBP; CANX; RACK1; PABPC1; TXNL4A; EIF3D; RAN
**E2F Targets**	2.72E-10	TOP2A; NOP56; DNMT1; TUBB; HMGB2; PSIP1; NAP1L1; PDS5B; SSRP1; MKI67; SMC3; SMC4; NME1; PNN; NASP; RAD21; LUC7L3; LYAR; RAN
**G2-M Checkpoint**	8.21E-08	TOP2A; SMARCC1; ATRX; PDS5B; MKI67; SMC4; SMC2; CENPF; SFPQ; NASP; DKC1; RAD21; KIF5B; NCL; TOP1; KIF20B
**Unfolded Protein Response**	6.06E-05	HSPA9; NOP56; RPS14; NOP14; NPM1; DKC1; KIF5B; HSP90B1; EIF4G1
**Myc Targets V2**	2.53E-04	NOP56; DDX18; NPM1; TCOF1; CBX3; HSPD1
**Mitotic Spindle**	2.58E-04	TOP2A; TIAM1; CENPF; CLIP1; STAU1; KIF5B; ITSN1; SMC3; KIF20B; SMC4; CDK5RAP2
**Oxidative Phosphorylation**	0.001	HSPA9; COX7B; ATP5F1A; NDUFA6; GPX4; NDUFS7; NDUFA4; NDUFA1; UQCR11; ATP5ME
**mTORC1 Signaling**	0.012	HSPA9; TPI1; CANX; PHGDH; ENO1; GAPDH; HSP90B1; HSPD1
**Apoptosis**	0.04	TOP2A; BTG3; KRT18; GPX4; LMNA; HMGB2
**Reactive Oxygen Species Pathway**	0.172	NDUFA6; GPX4
**Protein Secretion**	0.182	GOLGA4; KRT18; GNAS
**Hypoxia**	0.192	AKAP12; TPI1; ZNF292; ENO1; GAPDH
**Adipogenesis**	0.192	COX7B; GPX4; ITSN1; BAZ2A; UQCR11
**p53 Pathway**	0.192	PIDD1; GM2A; RACK1; UPP1; RPS12
**Allograft Rejection**	0.192	NPM1; RPS19; EIF3D; EIF3A; NME1
**DNA Repair**	0.197	GPX4; UPF3B; SSRP1; NME1
**Peroxisome**	0.213	TOP2A; SMARCC1; PABPC1
**PI3K/AKT/mTOR Signaling**	0.217	TIAM1; YWHAB; HSP90B1
**Estrogen Response Early**	0.366	TIAM1; KRT18; KRT8; PTGES
**Estrogen Response Late**	0.366	TOP2A; TIAM1; BTG3; PTGES
**Epithelial Mesenchymal Transition**	0.366	BASP1; CALD1; TPM1; CALU
**Glycolysis**	0.366	TPI1; NASP; ZNF292; ENO1
**Notch Signaling**	0.390	FBXW11
**Androgen Response**	0.453	AKAP12; KRT8
**Xenobiotic Metabolism**	0.593	ASL; UPP1; PTGES
**Spermatogenesis**	0.614	PEBP1; IP6K1
**UV Response Up**	0.7	BTG3; DDX21
**IL-2/STAT5 Signaling**	0.811	TIAM1; GPX4
**TNF-alpha Signaling via NF-kB**	0.813	BTG3; NFE2L2
**KRAS Signaling Up**	0.813	AKAP12; GADD45G
**Coagulation**	0.881	GNG12
**UV Response Dn**	0.9	ATRX
**Fatty Acid Metabolism**	0.913	HSP90AA1
**Interferon Gamma Response**	0.954	UPP1
**Apical Junction**	0.954	ARPC2
**Complement**	0.954	USP8
**heme Metabolism**	0.954	TOP1

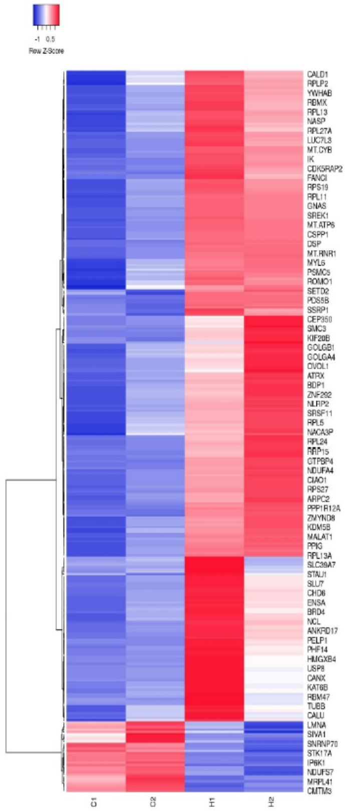

Supplementary Figure S2. Left - Table of the main pathways expressed in hypoxia microchip. Right - Heat-map of control microchip experiment: (C1, C2) and hypoxia microchip: (H1, H2). n=2

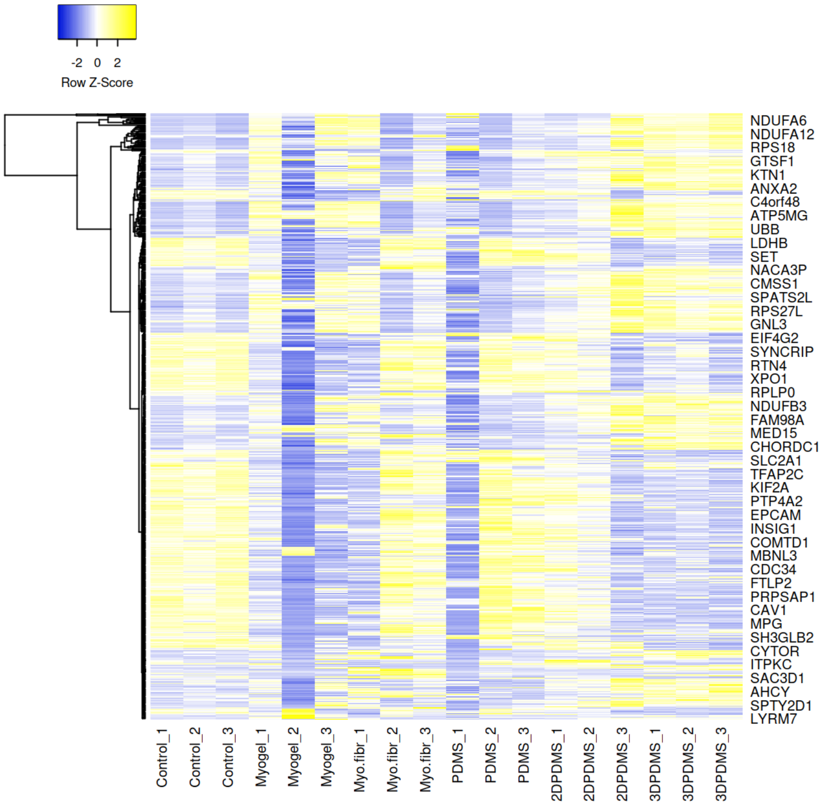

Supplementary Figure S3. Heatmap of the well plate experiment with significant genes. Myo.fibr=Myogel+Fibrin,2DPDMS=Myogel+PDMS,3DPDMS=Myogel+Fibrin+PDMS.(n=3)

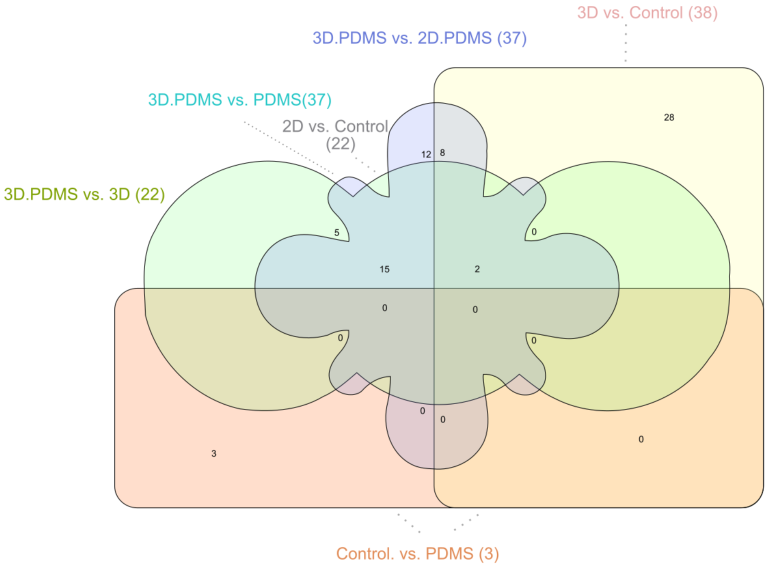

Supplementary Figure S4. Venn diagram for the significant pathways identified by MSigDB Hallmark 2020 database. The pathways are listed with p-values less than 0.05. 3D. vs. Control = Myogel + Fibrin vs. Control, 3D.PDMS vs. 3D = Myogel + Fibrin + PDMS vs, Myogel+Fibrin. 2D.vs. Control = Myogel vs. control, 3D.PDMD vs. 2D.PDMS = Myogel + Fibrin + PDMS vs. Myogel + PDMS, 3D.PDMS vs. PDMS = Myogel + Fibrin + PDMS vs. PDMS.

Genes	*baseMean*	*log2FoldChange*	*lfcSE*	*stat*	*pvalue*	*padj*
MT-RNR2	34000.55	2.17	0.26	8.38	5.49E-17	6.60E-14
MT-ND4	15772.68	1.81	0.23	7.83	5.02E-15	3.02E-12
MALAT1	25251.11	1.89	0.25	7.51	6.01E-14	2.41E-11
MT-CO2	14327.16	1.66	0.23	7.1	1.28E-12	3.21E-10
MT-CYB	7989.83	1.56	0.22	7.08	1.44E-12	3.21E-10
UQCR11	810.29	-1.56	0.22	-7.07	1.60E-12	3.21E-10
RBM25	10256.17	1.56	0.22	6.96	3.40E-12	5.84E-10
MT-ATP6	8446.72	1.5	0.22	6.89	5.64E-12	8.37E-10
PRRC2C	12672	1.64	0.24	6.87	6.26E-12	8.37E-10
SREK1	9137.96	1.49	0.22	6.66	2.79E-11	3.36E-09
PTMA	8767.35	1.44	0.22	6.6	4.20E-11	4.59E-09
MT-ND5	2750.76	1.26	0.19	6.52	7.04E-11	7.06E-09
RPS27	4634.76	1.24	0.2	6.2	5.56E-10	5.15E-08
MT-RNR1	4849.6	1.22	0.2	6.19	6.12E-10	5.26E-08
MT-CO3	7050.11	1.31	0.22	6.07	1.25E-09	1.00E-07
HSP90AA1	8108.71	1.35	0.22	6	1.96E-09	1.47E-07
IP6K1	554	-1.34	0.23	-5.96	2.56E-09	1.81E-07
MT-CO1	3718.81	1.15	0.19	5.89	3.89E-09	2.60E-07
RPL13A	9527.49	1.31	0.22	5.88	4.14E-09	2.62E-07
STK17A	672.74	-1.26	0.21	-5.86	4.57E-09	2.75E-07
MT-ND3	2912.98	1.19	0.2	5.84	5.16E-09	2.95E-07
RPS19	12614.53	1.35	0.23	5.78	7.30E-09	3.99E-07
MT-ATP8	3691.47	1.27	0.23	5.48	4.34E-08	2.27E-06
MT-ND1	2728.21	1.06	0.2	5.39	6.92E-08	3.22E-06
RPL37A	5098.09	1.06	0.2	5.39	6.96E-08	3.22E-06
SRRM1	3222.87	1.08	0.2	5.4	6.81E-08	3.22E-06
MTATP6P1	1271.15	1.06	0.2	5.35	8.74E-08	3.89E-06
ZC3H13	5606.84	1.17	0.22	5.32	1.02E-07	4.36E-06
ANKRD11	7247.7	1.16	0.22	5.3	1.15E-07	4.65E-06
CIAO1	2413.81	1.07	0.2	5.29	1.20E-07	4.65E-06
NCL	4303.97	1.11	0.21	5.3	1.18E-07	4.65E-06
TCOF1	7149.5	1.24	0.24	5.21	1.85E-07	6.75E-06
TUBB4B	4670.01	1.12	0.21	5.22	1.80E-07	6.75E-06
EIF5B	6150.01	1.11	0.22	5.11	3.16E-07	1.12E-05

Supplementary Table S1. DE genes in Hypoxia vs. Control microchip.

Genes	*baseMean*	*log2FoldChange*	*lfcSE*	*stat*	*pvalue*	*padj*
RPL6	4854.04	1	0.2	4.97	6.71E-07	2.24E-05
NDUFA1	2365.01	0.93	0.2	4.77	1.85E-06	6.02E-05
HSP90B1	2401.51	1.04	0.22	4.76	1.92E-06	6.08E-05
FTH1	8533.3	1.1	0.23	4.73	2.22E-06	6.86E-05
CHD6	2530.97	1.06	0.22	4.72	2.34E-06	7.05E-05
NUCKS1	3487.14	0.93	0.2	4.68	2.85E-06	8.35E-05
DDX21	3505.45	0.93	0.2	4.68	2.93E-06	8.39E-05
NFE2L2	607.65	-1.02	0.22	-4.66	3.18E-06	8.89E-05
C1QBP	469.75	-1.09	0.24	-4.62	3.78E-06	1.03E-04
RPS14	5870.54	0.98	0.21	4.62	3.92E-06	1.05E-04
ANP32B	1739.69	1	0.22	4.59	4.43E-06	1.16E-04
AP3D1	2727.52	0.95	0.21	4.53	5.92E-06	1.51E-04
USP8	1532.1	1.11	0.25	4.52	6.12E-06	1.53E-04
KRT18	5050.92	0.95	0.21	4.5	6.94E-06	1.70E-04
RPS12	6804.9	1.03	0.23	4.45	8.59E-06	2.07E-04
RPL11	5105.53	0.92	0.21	4.44	9.09E-06	2.15E-04
RPF2	2113.6	0.92	0.21	4.42	9.95E-06	2.30E-04
IARS2	545.82	-1.07	0.24	-4.38	1.21E-05	2.65E-04
PPP1R12A	1259.17	0.85	0.19	4.38	1.21E-05	2.65E-04
RPS24	4148.9	0.93	0.21	4.38	1.20E-05	2.65E-04
MT-ND2	1297.73	0.93	0.21	4.37	1.24E-05	2.67E-04
BTG3	564.52	-0.99	0.23	-4.35	1.35E-05	2.72E-04
EIF3A	2620.12	0.89	0.2	4.35	1.33E-05	2.72E-04
NAP1L1	1486.6	1.02	0.23	4.36	1.29E-05	2.72E-04
PRPF38B	4398.19	0.96	0.22	4.35	1.36E-05	2.72E-04
PHF14	795.16	1.02	0.24	4.33	1.50E-05	2.96E-04
RPL13	8562.69	0.97	0.23	4.29	1.81E-05	3.52E-04
RPL21	3815.95	0.84	0.2	4.28	1.86E-05	3.55E-04
RPSA	5200.96	0.95	0.22	4.25	2.12E-05	3.98E-04
MZT2B	636.4	-0.9	0.21	-4.23	2.35E-05	4.34E-04
RPL37	4159.45	0.86	0.2	4.22	2.41E-05	4.40E-04
LUC7L3	1857.45	0.83	0.2	4.22	2.46E-05	4.42E-04
CANX	1666.75	1	0.24	4.21	2.52E-05	4.43E-04
TAF3	2146.29	0.85	0.2	4.21	2.58E-05	4.43E-04

Supplementary Table S1 (continued). DE genes in Hypoxia vs. Control microchip.

Genes	*baseMean*	*log2FoldChange*	*lfcSE*	*stat*	*pvalue*	*padj*
NDUFA11	437.92	-1.1	0.26	-4.17	2.99E-05	5.00E-04
SET	3403.46	0.87	0.21	4.18	2.95E-05	5.00E-04
SLU7	624.15	0.98	0.24	4.17	3.05E-05	5.02E-04
BRD2	1409.44	0.83	0.2	4.15	3.30E-05	5.37E-04
HNRNPAB	863.79	-0.85	0.21	-4.12	3.85E-05	6.17E-04
ITSN2	1878.51	0.79	0.19	4.11	3.92E-05	6.21E-04
GNAS	2730.25	0.83	0.2	4.1	4.09E-05	6.39E-04
PPIG	2047.4	0.82	0.2	4.1	4.20E-05	6.47E-04
NDUFS7	662.05	-0.9	0.22	-4.04	5.40E-05	8.22E-04
IRX2	434.82	-0.91	0.23	-4.03	5.68E-05	8.54E-04
HSP90AB1	2733.49	0.82	0.2	4	6.32E-05	9.34E-04
RPS8	6240.2	0.86	0.22	4	6.37E-05	9.34E-04
CENPF	2874.62	0.8	0.2	3.96	7.51E-05	1.09E-03
MED12L	546	0.84	0.21	3.95	7.69E-05	1.10E-03
RPS6	3804.26	0.76	0.2	3.87	1.08E-04	1.53E-03
PEBP1	3507.14	0.8	0.21	3.86	1.12E-04	1.57E-03
CSPP1	907.87	0.79	0.21	3.82	1.31E-04	1.81E-03
KMT2C	945.13	0.78	0.2	3.82	1.34E-04	1.83E-03
DDX18	1144.04	0.77	0.2	3.82	1.36E-04	1.83E-03
MKI67	2936.44	0.77	0.2	3.8	1.43E-04	1.92E-03
ENSA	2229.87	0.82	0.22	3.79	1.51E-04	1.99E-03
ABCF1	3638.93	0.8	0.21	3.78	1.55E-04	2.03E-03
RPS27A	4329.49	0.76	0.2	3.78	1.57E-04	2.04E-03
USP42	1444.21	0.82	0.22	3.77	1.66E-04	2.13E-03
GAPDH	3917.7	0.84	0.22	3.75	1.77E-04	2.22E-03
MAP4K4	1580.39	0.77	0.21	3.75	1.77E-04	2.22E-03
SIVA1	1492.41	-0.87	0.23	-3.74	1.84E-04	2.28E-03
SELENOH	601.3	-0.79	0.21	-3.71	2.11E-04	2.58E-03
PSMC1	925.95	0.88	0.24	3.68	2.29E-04	2.78E-03
RPL7A	2919.29	0.7	0.19	3.67	2.41E-04	2.90E-03
BAZ1B	1766.78	0.72	0.2	3.66	2.52E-04	3.00E-03
KMT2B	719.16	1.01	0.28	3.65	2.60E-04	3.07E-03
BRD4	2240.64	0.81	0.22	3.64	2.72E-04	3.16E-03
ITSN1	1280.76	0.72	0.2	3.64	2.73E-04	3.16E-03

Supplementary Table S1 (continued). DE genes in Hypoxia vs. Control microchip.

Genes	*baseMean*	*log2FoldChange*	*lfcSE*	*stat*	*pvalue*	*padj*
NACA	2478.62	0.77	0.21	3.61	3.05E-04	3.45E-03
PBDC1	435.33	0.94	0.26	3.61	3.07E-04	3.45E-03
KRT8	8247.37	0.85	0.24	3.6	3.23E-04	3.60E-03
RPL5	3716.89	0.76	0.21	3.58	3.47E-04	3.83E-03
SEPTIN11	1826.65	0.7	0.2	3.57	3.62E-04	3.96E-03
RPL4	1561.22	0.71	0.2	3.55	3.85E-04	4.17E-03
DNMT1	2319.64	0.75	0.21	3.54	3.99E-04	4.25E-03
PABPC4	1660.82	0.72	0.2	3.54	3.97E-04	4.25E-03
IK	916.26	0.71	0.2	3.53	4.15E-04	4.37E-03
PNISR	833	0.76	0.22	3.5	4.64E-04	4.85E-03
SMARCC1	1636.35	0.74	0.21	3.5	4.68E-04	4.85E-03
TXNL4A	589.66	-0.79	0.23	-3.49	4.91E-04	5.05E-03
CHD2	2168.17	0.74	0.21	3.48	5.03E-04	5.09E-03
MRPL41	1340.13	-0.71	0.2	-3.48	5.02E-04	5.09E-03
NOP14	818.15	0.74	0.21	3.47	5.30E-04	5.31E-03
HMGB2	1223.59	0.76	0.22	3.46	5.39E-04	5.35E-03
ZMAT2	1194.57	0.78	0.23	3.46	5.43E-04	5.35E-03
UPF3B	900.7	0.71	0.2	3.45	5.63E-04	5.50E-03
PSMD7	1302.28	0.68	0.2	3.44	5.81E-04	5.64E-03
RPLP1	6406.85	0.79	0.23	3.43	6.01E-04	5.79E-03
NDUFA6	1495.97	0.69	0.2	3.43	6.13E-04	5.82E-03
NPM1	1503.06	0.67	0.2	3.43	6.14E-04	5.82E-03
GADD45G	657.76	-0.74	0.22	-3.41	6.52E-04	6.13E-03
KDM5B	1743.49	0.67	0.2	3.4	6.68E-04	6.23E-03
RPL35	8213.51	0.79	0.23	3.4	6.81E-04	6.30E-03
RPS25	1854.26	0.66	0.2	3.39	7.00E-04	6.43E-03
AKR1B1	1365.74	0.64	0.19	3.38	7.32E-04	6.62E-03
HSPD1	1955.33	0.63	0.19	3.38	7.27E-04	6.62E-03
BASP1	1256.68	0.78	0.23	3.36	7.72E-04	6.93E-03
SNRNP70	431.68	-0.76	0.22	-3.36	7.84E-04	6.93E-03
YWHAB	2652.96	0.65	0.19	3.36	7.80E-04	6.93E-03
ATP5MPL	1939.54	0.65	0.19	3.35	8.11E-04	7.12E-03
KAT6B	563.83	0.84	0.25	3.34	8.25E-04	7.19E-03
TOP1	1679.16	0.65	0.19	3.34	8.30E-04	7.19E-03

Supplementary Table S1 (continued). DE genes in Hypoxia vs. Control microchip.

Genes	*baseMean*	*log2FoldChange*	*lfcSE*	*stat*	*pvalue*	*padj*
NOP58	1295.84	0.71	0.21	3.33	8.67E-04	7.44E-03
RPL27A	5325.35	0.74	0.22	3.33	8.78E-04	7.44E-03
SRSF11	2122.19	0.7	0.21	3.32	8.84E-04	7.44E-03
COL9A3	393.42	-0.77	0.23	-3.3	9.75E-04	8.09E-03
SMC4	2077.88	0.66	0.2	3.3	9.75E-04	8.09E-03
RPL12	2548.91	0.65	0.2	3.29	1.00E-03	8.25E-03
TOP2A	2567.65	0.67	0.2	3.29	1.01E-03	8.27E-03
LMNA	834.64	-0.75	0.23	-3.28	1.02E-03	8.31E-03
RBBP6	1623.03	0.69	0.21	3.26	1.12E-03	8.97E-03
RBM47	1155.01	0.76	0.23	3.26	1.12E-03	8.97E-03
RPS13	1729.37	0.64	0.2	3.26	1.13E-03	8.97E-03
NLRP2	1696.6	0.66	0.2	3.25	1.14E-03	8.99E-03
PTGES	2624.92	0.71	0.22	3.25	1.17E-03	9.19E-03
NOP56	1103.36	0.68	0.21	3.22	1.29E-03	1.01E-02
GIGYF2	1423.17	0.66	0.21	3.22	1.30E-03	1.01E-02
CCAR1	1284.43	0.63	0.2	3.21	1.33E-03	1.02E-02
RSL1D1	865.31	0.67	0.21	3.21	1.33E-03	1.02E-02
CCDC174	903.01	0.66	0.21	3.2	1.36E-03	1.03E-02
WDR60	651.32	0.79	0.25	3.2	1.38E-03	1.04E-02
MYO10	406.01	0.78	0.24	3.18	1.47E-03	1.11E-02
JUND	472.94	-0.71	0.22	-3.17	1.54E-03	1.14E-02
SYNE2	2082.66	0.64	0.2	3.17	1.55E-03	1.14E-02
UPF2	1898.19	0.67	0.21	3.17	1.54E-03	1.14E-02
ASH1L	478.07	0.68	0.22	3.15	1.65E-03	1.21E-02
DKC1	1367.04	0.69	0.22	3.14	1.70E-03	1.21E-02
NACA3P	1677.79	0.73	0.23	3.14	1.67E-03	1.21E-02
NCOR1	585.7	0.66	0.21	3.14	1.68E-03	1.21E-02
PIDD1	570.5	-0.75	0.24	-3.14	1.69E-03	1.21E-02
RPL24	2000.47	0.6	0.19	3.14	1.69E-03	1.21E-02
ROMO1	4502.94	0.67	0.21	3.13	1.72E-03	1.21E-02
TPM1	5507.33	0.73	0.23	3.13	1.72E-03	1.21E-02
NDUFA4	2360.14	0.59	0.19	3.13	1.75E-03	1.22E-02
SFPQ	1451.12	0.65	0.21	3.13	1.77E-03	1.22E-02
SRSF4	718.33	0.9	0.29	3.13	1.76E-03	1.22E-02

Supplementary Table S1 (continued). DE genes in Hypoxia vs. Control microchip.

Genes	*baseMean*	*log2FoldChange*	*lfcSE*	*stat*	*pvalue*	*padj*
RPL35A	2756.76	0.62	0.2	3.11	1.85E-03	1.26E-02
TUBA1B	2090.89	0.65	0.21	3.11	1.84E-03	1.26E-02
CALD1	3578.32	0.66	0.21	3.1	1.94E-03	1.31E-02
CBX3	2954.96	0.64	0.21	3.1	1.94E-03	1.31E-02
COX7B	1595.91	0.59	0.19	3.08	2.09E-03	1.40E-02
PELP1	770.9	0.65	0.21	3.07	2.16E-03	1.43E-02
RPL19	2264.21	0.59	0.19	3.07	2.16E-03	1.43E-02
EIF4B	1030.57	0.65	0.21	3.06	2.19E-03	1.43E-02
YAP1	552.7	0.71	0.23	3.06	2.19E-03	1.43E-02
MAN1A2	704.22	0.64	0.21	3.03	2.42E-03	1.58E-02
TIAM1	689.2	0.78	0.26	3.01	2.58E-03	1.67E-02
ZNF292	1766.63	0.62	0.21	3.01	2.62E-03	1.69E-02
LARP1	1548.21	0.63	0.21	3	2.70E-03	1.73E-02
RPL10A	2564.56	0.61	0.2	3	2.73E-03	1.74E-02
KNOP1	1045.12	0.62	0.21	2.99	2.78E-03	1.76E-02
CCSAP	542.82	0.63	0.21	2.97	2.96E-03	1.87E-02
ARPC2	1610.73	0.58	0.2	2.95	3.14E-03	1.97E-02
KIF20B	948.52	0.61	0.21	2.94	3.28E-03	2.05E-02
EIF4G1	1293.05	0.59	0.2	2.94	3.33E-03	2.06E-02
GPATCH4	1324.73	0.62	0.21	2.93	3.42E-03	2.11E-02
REST	1489.31	0.6	0.2	2.92	3.48E-03	2.14E-02
CCDC34	1108.96	0.59	0.2	2.92	3.51E-03	2.14E-02
SMC2	863.86	0.58	0.2	2.92	3.52E-03	2.14E-02
NEAT1	3147.83	0.63	0.22	2.91	3.61E-03	2.18E-02
EIF4E2	426	0.66	0.23	2.91	3.66E-03	2.20E-02
SON	2328.12	0.61	0.21	2.9	3.71E-03	2.22E-02
CDK5RAP2	788.8	0.59	0.2	2.89	3.86E-03	2.29E-02
GNG12	403.12	0.71	0.25	2.89	3.85E-03	2.29E-02
LARP7	731.77	0.59	0.2	2.89	3.91E-03	2.31E-02
ANKRD17	795.69	0.6	0.21	2.87	4.13E-03	2.42E-02
LYAR	392.48	0.71	0.25	2.86	4.21E-03	2.46E-02
RPL23	3017.73	0.58	0.2	2.86	4.24E-03	2.46E-02
FAM32A	1208.71	0.57	0.2	2.86	4.30E-03	2.48E-02
RPL26	1242.89	0.61	0.21	2.86	4.30E-03	2.48E-02

Supplementary Table S1 (continued). DE genes in Hypoxia vs. Control microchip.

Genes	*baseMean*	*log2FoldChange*	*lfcSE*	*stat*	*pvalue*	*padj*
RAN	1836.9	0.58	0.2	2.85	4.35E-03	2.48E-02
RRP15	757.33	0.59	0.21	2.84	4.45E-03	2.53E-02
MYL6	2040.82	0.56	0.2	2.84	4.52E-03	2.55E-02
HSPA9	2033.87	0.57	0.2	2.84	4.58E-03	2.57E-02
CLIP1	1450.83	0.57	0.2	2.83	4.70E-03	2.63E-02
PSIP1	825.6	0.58	0.21	2.82	4.82E-03	2.68E-02
PTN	557.07	0.59	0.21	2.81	4.89E-03	2.70E-02
RPS4X	1667.49	0.57	0.2	2.82	4.87E-03	2.70E-02
GOLGA4	1686.3	0.59	0.21	2.8	5.06E-03	2.78E-02
PDS5B	660.53	0.57	0.21	2.8	5.14E-03	2.81E-02
SSRP1	865.53	0.56	0.2	2.79	5.26E-03	2.86E-02
GPX4	1000.5	-0.68	0.24	-2.79	5.30E-03	2.87E-02
GM2A	915.85	0.56	0.2	2.79	5.34E-03	2.88E-02
CALU	1119.96	0.66	0.24	2.78	5.40E-03	2.90E-02
EIF3D	412.77	0.62	0.22	2.78	5.44E-03	2.91E-02
ZMYND8	1629.29	0.54	0.2	2.76	5.70E-03	3.03E-02
NEMF	844.75	0.6	0.22	2.76	5.76E-03	3.04E-02
RPS15A	2473.8	0.57	0.21	2.76	5.74E-03	3.04E-02
BDP1	1366.96	0.56	0.2	2.76	5.84E-03	3.04E-02
FANCI	643.49	0.58	0.21	2.76	5.84E-03	3.04E-02
SSB	1002.36	0.54	0.2	2.76	5.84E-03	3.04E-02
ATP5ME	2525.34	0.57	0.21	2.75	5.97E-03	3.06E-02
ATRX	1265.36	0.55	0.2	2.75	6.02E-03	3.06E-02
CWC25	496.01	0.61	0.22	2.75	5.98E-03	3.06E-02
ENO1	2016.83	0.52	0.19	2.75	5.96E-03	3.06E-02
POLDIP2	1058.34	0.59	0.21	2.75	6.01E-03	3.06E-02
TBCA	1097.19	0.57	0.21	2.75	5.96E-03	3.06E-02
RBMX	1684.36	0.54	0.2	2.74	6.13E-03	3.10E-02
HIST1H1E	611.82	-0.63	0.23	-2.73	6.31E-03	3.16E-02
RIF1	1308.81	0.54	0.2	2.73	6.30E-03	3.16E-02
SEPTIN7	817.87	0.56	0.2	2.73	6.37E-03	3.18E-02
SMC3	979.42	0.55	0.2	2.73	6.40E-03	3.18E-02
RPL31	1931.08	0.51	0.19	2.72	6.49E-03	3.21E-02
KDM5A	719.9	0.56	0.2	2.72	6.53E-03	3.22E-02

Supplementary Table S1 (continued). DE genes in Hypoxia vs. Control microchip.

Genes	*baseMean*	*log2FoldChange*	*lfcSE*	*stat*	*pvalue*	*padj*
CD3EAP	1470.39	0.68	0.25	2.71	6.69E-03	3.26E-02
OVOL1	2002.07	0.54	0.2	2.71	6.74E-03	3.26E-02
PSMB6	1397.48	0.55	0.2	2.71	6.73E-03	3.26E-02
SETD2	496.79	0.59	0.22	2.71	6.73E-03	3.26E-02
AKAP12	1169.89	0.56	0.21	2.71	6.80E-03	3.27E-02
UPP1	1104.28	-0.59	0.22	-2.7	6.92E-03	3.32E-02
ASL	527.59	-0.68	0.25	-2.69	7.07E-03	3.37E-02
GATA2	3004.08	0.56	0.21	2.68	7.29E-03	3.46E-02
PHGDH	836.88	0.55	0.21	2.68	7.34E-03	3.47E-02
RPL10	2098.06	0.53	0.2	2.67	7.51E-03	3.54E-02
NASP	2138.46	0.52	0.2	2.67	7.68E-03	3.60E-02
SLTM	1294.89	0.54	0.2	2.67	7.69E-03	3.60E-02
MRPL52	2415.28	0.53	0.2	2.66	7.90E-03	3.67E-02
TUBB	2060.87	0.58	0.22	2.66	7.87E-03	3.67E-02
RPS16	4708.1	0.58	0.22	2.65	7.98E-03	3.69E-02
PKMYT1	472.02	0.57	0.22	2.65	8.04E-03	3.71E-02
KIAA1217	1207.74	0.57	0.21	2.64	8.17E-03	3.74E-02
TAF11	565.77	0.57	0.21	2.65	8.16E-03	3.74E-02
PITHD1	569.52	0.58	0.22	2.64	8.36E-03	3.81E-02
RACK1	4352.46	0.59	0.23	2.63	8.49E-03	3.85E-02
RPL41	1703.03	0.52	0.2	2.63	8.51E-03	3.85E-02
TBC1D10B	400.7	0.73	0.28	2.63	8.65E-03	3.90E-02
HMGXB4	410.19	0.62	0.24	2.62	8.71E-03	3.91E-02
NME1	1543.29	0.59	0.23	2.61	8.95E-03	4.00E-02
PARD3	979.97	0.55	0.21	2.61	9.08E-03	4.05E-02
CHD9	450.05	0.57	0.22	2.6	9.28E-03	4.11E-02
RALY	971.46	-0.55	0.21	-2.6	9.26E-03	4.11E-02
GNL3L	651.8	0.57	0.22	2.6	9.33E-03	4.11E-02
KIF5B	833.04	0.54	0.21	2.6	9.36E-03	4.11E-02
ZNF638	790.37	0.55	0.21	2.59	9.52E-03	4.16E-02
RPLP2	4673.09	0.58	0.22	2.59	9.56E-03	4.17E-02
SF3B2	813.75	0.57	0.22	2.59	9.61E-03	4.18E-02
CHD1	647.92	0.54	0.21	2.59	9.66E-03	4.18E-02
AKAP9	1028.38	0.52	0.2	2.58	9.78E-03	4.21E-02

Supplementary Table S1 (continued). DE genes in Hypoxia vs. Control microchip.

Genes	*baseMean*	*log2FoldChange*	*lfcSE*	*stat*	*pvalue*	*padj*
SPEN	1075.65	0.58	0.22	2.58	9.80E-03	4.21E-02
BAZ1A	996.95	0.51	0.2	2.58	9.88E-03	4.21E-02
U2SURP	794.3	0.52	0.2	2.58	9.87E-03	4.21E-02
EBNA1BP2	537.38	0.55	0.21	2.58	9.98E-03	4.24E-02
GOLGB1	1159.18	0.54	0.21	2.57	1.00E-02	4.25E-02
GTPBP4	469.43	0.56	0.22	2.57	1.02E-02	4.29E-02
KIF14	683.86	0.54	0.21	2.57	1.02E-02	4.30E-02
RPS19BP1	1027.15	-0.55	0.21	-2.55	1.06E-02	4.46E-02
FBXW11	580.55	-0.57	0.22	-2.55	1.07E-02	4.46E-02
METAP2	1941.96	0.55	0.22	2.55	1.07E-02	4.46E-02
DROSHA	483	0.61	0.24	2.54	1.10E-02	4.53E-02
RAD21	533.99	0.55	0.21	2.55	1.09E-02	4.53E-02
TPI1	1735.21	0.57	0.22	2.54	1.11E-02	4.59E-02
RRP1B	1237.34	0.57	0.22	2.54	1.12E-02	4.61E-02
CEP350	633.87	0.55	0.22	2.53	1.13E-02	4.64E-02
BAZ2A	1416.37	0.53	0.21	2.53	1.15E-02	4.68E-02
PNN	977.19	0.51	0.2	2.53	1.15E-02	4.68E-02
STAU1	608.82	0.57	0.23	2.52	1.17E-02	4.73E-02
PABPC1	1011.53	0.49	0.2	2.52	1.18E-02	4.75E-02
BOD1L1	794.6	0.51	0.2	2.51	1.20E-02	4.81E-02
DNAJC2	608.37	0.53	0.21	2.51	1.20E-02	4.83E-02
DDX27	1317.7	0.52	0.21	2.51	1.21E-02	4.85E-02
MRPL21	558.81	0.53	0.21	2.5	1.23E-02	4.88E-02
TARS	723.87	0.54	0.22	2.5	1.23E-02	4.88E-02
PSMC5	1283.18	0.5	0.2	2.5	1.24E-02	4.92E-02
HNRNPM	2179.69	0.64	0.26	2.49	1.27E-02	4.98E-02
SLC39A7	818.47	0.65	0.26	2.49	1.26E-02	4.98E-02
MT-ND4L	1159.74	0.96	0.19	5.01	5.35E-07	1.84E-05
THOC2	1330.48	0.84	0.2	4.21	2.55E-05	4.43E-04
SEC62	1245.65	0.71	0.2	3.63	2.79E-04	3.20E-03
CMTM3	737.22	-0.7	0.21	-3.33	8.79E-04	7.44E-03
DSP	1059.52	0.61	0.2	3.11	1.85E-03	1.26E-02
DDX46	2144.83	0.57	0.2	2.85	4.33E-03	2.48E-02
ATP5F1A	1160.07	0.59	0.22	2.71	6.66E-03	3.26E-02

Supplementary Table S1 (continued). DE genes in Hypoxia vs. Control microchip.

Term	Our P-value	Zhu et al. P-value
Myc Targets V1	6.29E-15	2.30E-15
E2F Targets	2.72E-10	0.087
G2-M Checkpoint	8.21E-08	0.009
Unfolded Protein Response	6.06E-05	3.80E-04
Myc Targets V2	2.53E-04	0.148
Oxidative Phosphorylation	0.001	0.087
mTORC1 Signaling	0.012	1.16E-18
Apoptosis	0.037	0.003
Reactive Oxygen Species Pathway	0.172	0.02
Protein Secretion	0.182	0.311
Hypoxia	0.192	1.16E-29
Adipogenesis	0.192	1.33E-04
p53 Pathway	0.192	1.97E-09
Allograft Rejection	0.192	0.087
DNA Repair	0.197	0.258
Peroxisome	0.213	0.708
PI3K/AKT/mTOR Signaling	0.217	0.008
Estrogen Response Early	0.366	0.009
Estrogen Response Late	0.366	0.087
Glycolysis	0.366	2.37E-10
Androgen Response	0.453	0.006
Xenobiotic Metabolism	0.593	0.907
UV Response Up	0.7	0.011
IL-2/STAT5 Signaling	0.811	0.002
TNF-alpha Signaling via NF-kB	0.813	6.19E-04
KRAS Signaling Up	0.813	0.418
Coagulation	0.881	0.221
UV Response Dn	0.892	0.027
Fatty Acid Metabolism	0.913	0.011
Interferon Gamma Response	0.954	0.087
Apical Junction	0.954	0.21
Complement	0.954	0.209
Heme Metabolism	0.954	0.209

Supplementary Table S2. Similar pathways in our study with hypoxia microchip compared to a study with hypoxia chamber.

Genes	*baseMean*	*log2FoldChange*	*lfcSE*	*stat*	*pvalue*	*padj*
CRIP2	72.23	1.37	0.21	6.36	1.98E-10	1.43E-06
EMP2	46.57	-1.34	0.23	-5.85	5.01E-09	1.80E-05
MLLT1	38.57	1.34	0.24	5.69	1.25E-08	2.99E-05
SP6	36.17	1.2	0.23	5.28	1.26E-07	0.0002267
MFAP5	34.2	-1.21	0.24	-5.15	2.66E-07	0.0003004
SLC7A4	79.61	-1.19	0.23	-5.19	2.11E-07	0.0003004
SPTBN1	35.01	-1.15	0.22	-5.13	2.92E-07	0.0003004
IRX4	62.78	1.09	0.21	5.08	3.75E-07	0.0003374
PLEKHF1	92.48	0.97	0.2	4.97	6.80E-07	0.0005431
OVOL1	30.05	1.3	0.28	4.7	2.58E-06	0.00183
SLC2A1	63.06	1.02	0.22	4.69	2.80E-06	0.00183
ARL4C	12.48	1.32	0.28	4.63	3.68E-06	0.002204
CTSV	69.91	-1.12	0.25	-4.57	4.88E-06	0.002702
ATP2B4	79.12	0.94	0.21	4.44	9.12E-06	0.004572
CD81	38.59	1.24	0.28	4.43	9.54E-06	0.004572
EFNA1	64.01	-1.13	0.26	-4.4	1.10E-05	0.004934
CEP131	31.99	0.98	0.22	4.34	1.41E-05	0.005713
HAND1	13.58	-1.24	0.29	-4.33	1.51E-05	0.005713
KRT23	23.31	1.14	0.26	4.34	1.44E-05	0.005713
SH3GLB2	42.08	1	0.23	4.3	1.70E-05	0.006106
GATA2	86.31	0.88	0.21	4.22	2.41E-05	0.00825
OLFML1	10	-1.18	0.29	-4.13	3.59E-05	0.01123
SLC22A31	9.7	1.18	0.29	4.13	3.59E-05	0.01123
SMARCA2	29.7	1.04	0.25	4.12	3.79E-05	0.01135
MYLPF	59.53	-0.98	0.24	-4.06	4.91E-05	0.01412
EPS8L1	29.17	0.96	0.24	3.99	6.72E-05	0.01859
LAYN	9.32	-1.12	0.28	-3.94	8.14E-05	0.02167
CGA	419.23	1.04	0.27	3.91	9.29E-05	0.02302
SFRP1	168.25	-0.92	0.23	-3.91	9.08E-05	0.02302
EIF2AK4	125.98	-0.82	0.21	-3.83	0.0001304	0.03126

Supplementary Table S3. DE genes in PDMS vs. Control.

Genes	*baseMean*	*log2FoldChange*	*lfcSE*	*stat*	*pvalue*	*padj*
CSRP2	25.68	-1	0.26	-3.81	0.0001411	0.03267
ZDHHC3	29.28	0.93	0.25	3.8	0.0001454	0.03267
FBXL15	22.12	0.93	0.25	3.77	0.0001617	0.03454
IFNAR1	12.38	-1.07	0.29	-3.77	0.0001633	0.03454
DCTD	50.34	-0.78	0.21	-3.75	0.000174	0.03575
EPB41L3	19.33	0.96	0.26	3.68	0.0002298	0.04589
MXD1	17.11	0.95	0.26	3.66	0.0002537	0.0493
VWA1	120.44	0.87	0.24	3.65	0.0002633	0.04982

Supplementary Table S3(continued). DE genes in PDMS vs. Control.

Genes	*baseMean*	*log2FoldChange*	*lfcSE*	*stat*	*pvalue*	*padj*
CDC20	101.2	-1.3	0.33	-3.98	7.02E-05	4.22E-03
RPL37	353.58	0.98	0.25	3.97	7.07E-05	4.22E-03
NDUFA10	47.16	-1.51	0.38	-3.95	7.93E-05	4.64E-03
ATP5PF	356.83	0.88	0.22	3.92	8.73E-05	4.68E-03
COX7A2	758.62	1.21	0.31	3.93	8.67E-05	4.68E-03
EIF4G2	59.87	-1.2	0.31	-3.92	8.91E-05	4.68E-03
FST	16.41	-1.97	0.5	-3.94	8.19E-05	4.68E-03
SF3B1	30.95	-1.61	0.41	-3.93	8.52E-05	4.68E-03
TMEM106C	35.07	-1.69	0.43	-3.92	8.92E-05	4.68E-03
KIF2A	26.54	-1.6	0.41	-3.9	9.75E-05	5.03E-03
YWHAH	89.09	-1.29	0.33	-3.89	1.01E-04	5.11E-03
C7orf50	48.05	-1.59	0.41	-3.87	1.07E-04	5.33E-03
RGS16	98.03	-1.12	0.29	-3.85	1.17E-04	5.71E-03
AKR1B1	190.31	-1.05	0.27	-3.82	1.32E-04	6.17E-03
COX8A	289.96	0.77	0.2	3.83	1.31E-04	6.17E-03
SNRPE	114.52	0.97	0.25	3.83	1.31E-04	6.17E-03
NEK6	64.66	-1.3	0.34	-3.81	1.40E-04	6.44E-03
GINS2	68.74	-1.14	0.3	-3.8	1.43E-04	6.47E-03
CAV1	15.49	-1.84	0.49	-3.78	1.58E-04	6.94E-03
FTH1P11	52.84	-1.29	0.34	-3.76	1.67E-04	6.94E-03
KRT19	258.53	-1.2	0.32	-3.78	1.55E-04	6.94E-03
PPIB	79.68	-1.05	0.28	-3.77	1.66E-04	6.94E-03
TAF5	26.12	-1.54	0.41	-3.77	1.62E-04	6.94E-03
ZNRD2	348.52	1.09	0.29	3.77	1.63E-04	6.94E-03
TALDO1	43.9	-1.46	0.39	-3.75	1.79E-04	7.37E-03
NDUFC1	91.25	1.18	0.32	3.73	1.91E-04	7.71E-03
PRXL2C	19.06	-1.84	0.49	-3.73	1.93E-04	7.71E-03
EEF1D	164.1	-0.99	0.27	-3.72	1.95E-04	7.72E-03
PRPSAP1	16.23	-1.81	0.49	-3.72	1.99E-04	7.77E-03
EMC8	33.04	-1.47	0.4	-3.71	2.05E-04	7.89E-03
LRPAP1	101.46	-1.12	0.3	-3.69	2.28E-04	8.66E-03
ITPA	31.9	-1.39	0.38	-3.68	2.33E-04	8.74E-03
SNX3	35.38	-1.34	0.37	-3.66	2.55E-04	9.44E-03
NDUFB7	525.4	0.77	0.21	3.64	2.75E-04	9.91E-03

Supplementary Table S4. DE genes in Myogel vs. Control.

Genes	*baseMean*	*log2FoldChange*	*lfcSE*	*stat*	*pvalue*	*padj*
POLR2K	38.87	1.23	0.34	3.63	2.80E-04	9.91E-03
USP39	26.31	-1.6	0.44	-3.63	2.79E-04	9.91E-03
WWC2	37.49	-1.39	0.38	-3.64	2.74E-04	9.91E-03
SNRPG	100.04	1.02	0.28	3.62	2.92E-04	1.02E-02
ALG3	50.97	-1.41	0.39	-3.59	3.33E-04	1.12E-02
POLD2	56.12	-1.51	0.42	-3.59	3.25E-04	1.12E-02
REST	22.39	-1.74	0.49	-3.59	3.31E-04	1.12E-02
SON	193.81	1.42	0.4	3.59	3.35E-04	1.12E-02
AIMP2	85.32	-1.06	0.29	-3.58	3.40E-04	1.12E-02
MLF2	70.51	-1.34	0.38	-3.57	3.51E-04	1.15E-02
PRDX6	73.06	-1.2	0.33	-3.57	3.55E-04	1.15E-02
NDUFA3	163.1	0.86	0.24	3.56	3.64E-04	1.15E-02
UROS	18.04	-1.63	0.46	-3.57	3.62E-04	1.15E-02
UQCRQ	894.51	1.13	0.32	3.55	3.82E-04	1.20E-02
RCOR1	96.13	-1.06	0.3	-3.55	3.88E-04	1.21E-02
IMP3	34.14	-1.37	0.39	-3.54	3.99E-04	1.23E-02
UQCRC1	196.88	-1.06	0.3	-3.53	4.12E-04	1.25E-02
DCXR	221.44	-0.79	0.22	-3.53	4.21E-04	1.27E-02
SDR39U1	13.24	-1.88	0.54	-3.51	4.52E-04	1.35E-02
YBX1	95.43	-1.43	0.41	-3.51	4.56E-04	1.35E-02
DCUN1D5	17.96	-1.67	0.48	-3.49	4.76E-04	1.39E-02
URM1	57.2	-1.34	0.38	-3.49	4.86E-04	1.41E-02
HDHD5	14.06	-1.83	0.53	-3.48	4.99E-04	1.43E-02
CFL1	88.4	-1.18	0.34	-3.47	5.19E-04	1.47E-02
RPL23	128.04	0.9	0.26	3.47	5.22E-04	1.47E-02
NDUFAB1	300.59	0.76	0.22	3.47	5.30E-04	1.48E-02
ANXA2	268.46	-0.87	0.25	-3.43	6.00E-04	1.51E-02
ATP5MDP1	16.51	1.74	0.51	3.45	5.69E-04	1.51E-02
BOLA3	73.07	1.01	0.29	3.44	5.87E-04	1.51E-02
CNOT7	25.83	-1.5	0.43	-3.44	5.76E-04	1.51E-02
ELOB	651.97	1.02	0.3	3.45	5.62E-04	1.51E-02
LDHB	95.33	-1.04	0.3	-3.43	5.99E-04	1.51E-02
MICOS10	133.51	0.98	0.28	3.45	5.56E-04	1.51E-02
MRPL37	111.77	-1.03	0.3	-3.46	5.47E-04	1.51E-02

Supplementary Table S4 (Continued). DE genes in Myogel vs. Control.

Genes	*baseMean*	*log2FoldChange*	*lfcSE*	*stat*	*pvalue*	*padj*
PHPT1	1085.92	1.11	0.32	3.43	5.98E-04	1.51E-02
RPS19	1654.46	0.84	0.24	3.44	5.71E-04	1.51E-02
SNRPF	89.88	0.92	0.27	3.45	5.55E-04	1.51E-02
WDR45	14.01	-1.79	0.52	-3.44	5.88E-04	1.51E-02
ALKBH7	66.75	-1.21	0.35	-3.43	6.05E-04	1.51E-02
FBL	105.09	-1	0.29	-3.42	6.19E-04	1.53E-02
CHD1	34.92	-1.42	0.42	-3.42	6.36E-04	1.56E-02
DYNC1LI2	53.66	-1.15	0.34	-3.41	6.50E-04	1.56E-02
HSBP1	289.2	0.9	0.26	3.41	6.50E-04	1.56E-02
PEBP1	153.52	-0.94	0.28	-3.41	6.51E-04	1.56E-02
RAB11FIP1	95.61	-1.06	0.31	-3.4	6.73E-04	1.60E-02
PFN1	374.98	0.78	0.23	3.4	6.83E-04	1.61E-02
PERP	33.42	-1.36	0.4	-3.39	7.09E-04	1.65E-02
SLC25A4	43.42	-1.38	0.41	-3.39	7.10E-04	1.65E-02
ATP1B3	17.81	-1.73	0.51	-3.37	7.42E-04	1.70E-02
LMO4	18.09	-1.56	0.46	-3.37	7.41E-04	1.70E-02
MRPS6	20.9	-1.61	0.48	-3.36	7.68E-04	1.73E-02
NANS	38.07	-1.52	0.45	-3.36	7.79E-04	1.73E-02
NUDT16L1	24.78	-1.64	0.49	-3.36	7.72E-04	1.73E-02
PSMD8	105.57	-1.07	0.32	-3.36	7.73E-04	1.73E-02
QPRT	112.58	-0.99	0.29	-3.35	7.99E-04	1.76E-02
TCERG1	57.41	-1.23	0.37	-3.35	8.06E-04	1.76E-02
ECHS1	118.85	-1.08	0.32	-3.34	8.33E-04	1.80E-02
EIF5	250.52	-1.16	0.35	-3.34	8.48E-04	1.80E-02
FDPS	51.64	-1.13	0.34	-3.34	8.52E-04	1.80E-02
MLLT10	18.78	-1.58	0.47	-3.34	8.53E-04	1.80E-02
OAZ1	214.52	-0.99	0.3	-3.34	8.39E-04	1.80E-02
FTH1	415.75	-0.95	0.29	-3.33	8.78E-04	1.84E-02
MARCKSL1	25.6	-1.44	0.43	-3.32	8.94E-04	1.84E-02
SEM1	63.47	1.07	0.32	3.32	8.89E-04	1.84E-02
THOC6	23.33	-1.67	0.5	-3.32	8.89E-04	1.84E-02
BOD1	13.71	-1.71	0.52	-3.31	9.26E-04	1.86E-02
CPVL	53.63	-1.25	0.38	-3.32	9.16E-04	1.86E-02
SEL1L	25	1.27	0.38	3.32	9.09E-04	1.86E-02

Supplementary Table S4 (Continued). DE genes in Myogel vs. Control.

Genes	*baseMean*	*log2FoldChange*	*lfcSE*	*stat*	*pvalue*	*padj*
SUCLG1	65.54	-0.98	0.3	-3.31	9.27E-04	1.86E-02
ATPAF2	12.98	-1.73	0.53	-3.29	9.85E-04	1.95E-02
MBNL3	28.2	-1.45	0.44	-3.3	9.81E-04	1.95E-02
EXOSC3	35.78	-1.21	0.37	-3.29	1.00E-03	1.96E-02
TOMM5	40.24	1.39	0.42	3.29	1.00E-03	1.96E-02
NT5C	13.27	-1.65	0.5	-3.28	1.05E-03	2.02E-02
SGMS1	12.62	1.55	0.47	3.28	1.05E-03	2.02E-02
VPS4B	51.5	1.31	0.4	3.27	1.09E-03	2.08E-02
ZDHHC16	28.06	-1.44	0.44	-3.27	1.09E-03	2.08E-02
COX5B	1050.69	0.86	0.26	3.26	1.11E-03	2.09E-02
UQCRH	319.12	0.87	0.27	3.26	1.12E-03	2.10E-02
HMGCR	13.69	-1.65	0.51	-3.25	1.14E-03	2.10E-02
SINHCAF	62.31	-1.09	0.34	-3.25	1.14E-03	2.10E-02
THRAP3	120.34	-0.87	0.27	-3.26	1.13E-03	2.10E-02
COX7C	607.73	0.99	0.3	3.24	1.18E-03	2.14E-02
STUB1	71.21	-1.01	0.31	-3.25	1.17E-03	2.14E-02
WDR34	71.24	-1.14	0.35	-3.24	1.19E-03	2.15E-02
H1F0	15.38	-1.55	0.48	-3.23	1.22E-03	2.17E-02
RPS16	778.74	0.62	0.19	3.23	1.22E-03	2.17E-02
TUBB6	11.89	-1.84	0.57	-3.24	1.22E-03	2.17E-02
GABARAPL2	18.65	-1.6	0.5	-3.22	1.26E-03	2.23E-02
ATP6AP1	74.1	-1.2	0.37	-3.22	1.29E-03	2.27E-02
PDIA3	36.84	-1.37	0.42	-3.22	1.30E-03	2.27E-02
HSD17B10	107.01	-0.95	0.3	-3.21	1.34E-03	2.32E-02
HSP90B1	232.92	-0.77	0.24	-3.2	1.36E-03	2.32E-02
KLF3	36.45	-1.26	0.39	-3.2	1.36E-03	2.32E-02
RPL12	313.21	-0.98	0.3	-3.21	1.35E-03	2.32E-02
SPINT2	39.97	-1.16	0.36	-3.2	1.37E-03	2.32E-02
SRPK1	87.96	-0.96	0.3	-3.19	1.40E-03	2.36E-02
PGP	169.07	-0.8	0.25	-3.19	1.41E-03	2.37E-02
KDM5B	138.33	-1.02	0.32	-3.18	1.45E-03	2.42E-02
EIF2B4	37.11	-1.24	0.39	-3.18	1.47E-03	2.44E-02
PUF60	67.15	-1.13	0.36	-3.18	1.48E-03	2.45E-02
NDUFB3	33.09	1.2	0.38	3.17	1.50E-03	2.45E-02

Supplementary Table S4 (Continued). DE genes in Myogel vs. Control.

Genes	*baseMean*	*log2FoldChange*	*lfcSE*	*stat*	*pvalue*	*padj*
OGA	20.66	-1.54	0.49	-3.17	1.51E-03	2.45E-02
SRSF2	22.05	-1.48	0.46	-3.18	1.50E-03	2.45E-02
NOP14	40.87	-1.21	0.38	-3.17	1.52E-03	2.45E-02
BRI3BP	23.18	-1.52	0.48	-3.17	1.55E-03	2.45E-02
KDELR2	39.29	-1.18	0.37	-3.17	1.53E-03	2.45E-02
PSMC5	59.98	-1.11	0.35	-3.17	1.54E-03	2.45E-02
UBE2I	66.69	-1.1	0.35	-3.17	1.54E-03	2.45E-02
CAMTA1	94.2	0.91	0.29	3.16	1.59E-03	2.49E-02
CNBP	45.52	-1.23	0.39	-3.16	1.59E-03	2.49E-02
POP7	74.77	-0.89	0.28	-3.15	1.61E-03	2.51E-02
H2AFZ	145.71	-0.92	0.29	-3.15	1.63E-03	2.53E-02
FIBP	29.51	-1.42	0.45	-3.14	1.66E-03	2.57E-02
KRT18P10	39.46	-1.5	0.48	-3.14	1.70E-03	2.61E-02
SNU13	274.49	0.74	0.24	3.13	1.73E-03	2.64E-02
OSGEP	17.33	-1.55	0.49	-3.13	1.76E-03	2.67E-02
DDX18	121.57	0.96	0.31	3.12	1.78E-03	2.68E-02
MRPS21	237.61	0.92	0.3	3.13	1.78E-03	2.68E-02
EIF3F	19.02	-1.52	0.49	-3.12	1.84E-03	2.74E-02
RPL5	75.7	-1.01	0.32	-3.12	1.84E-03	2.74E-02
CDK2AP2	37.6	-1.1	0.35	-3.11	1.86E-03	2.76E-02
DCTPP1	89.44	-0.95	0.31	-3.1	1.94E-03	2.87E-02
SRSF7	21.97	-1.48	0.48	-3.1	1.95E-03	2.87E-02
AC015871.1	18.2	1.4	0.45	3.09	1.97E-03	2.87E-02
NDUFV1	52.74	-1.43	0.46	-3.09	1.97E-03	2.87E-02
MRPL48	22.17	-1.35	0.44	-3.09	1.98E-03	2.87E-02
CKAP5	27.08	-1.48	0.48	-3.08	2.09E-03	2.88E-02
EFNA1	32.5	-1.24	0.4	-3.08	2.06E-03	2.88E-02
FUCA2	32.54	-1.43	0.47	-3.07	2.16E-03	2.88E-02
GGNBP2	15.04	-1.5	0.49	-3.08	2.05E-03	2.88E-02
ILF2	40.97	-1.22	0.4	-3.09	2.00E-03	2.88E-02
KIN	12.65	-1.62	0.53	-3.07	2.16E-03	2.88E-02
LYRM7	44.45	1.62	0.53	3.07	2.14E-03	2.88E-02
MPG	15.17	-1.5	0.49	-3.08	2.05E-03	2.88E-02
MRPL3	17.79	-1.42	0.46	-3.07	2.15E-03	2.88E-02

Supplementary Table S4 (Continued). DE genes in Myogel vs. Control.

Genes	*baseMean*	*log2FoldChange*	*lfcSE*	*stat*	*pvalue*	*padj*
MRPL33	44.29	1.07	0.35	3.08	2.09E-03	2.88E-02
MRPL4	24.08	-1.43	0.47	-3.07	2.12E-03	2.88E-02
NFKBIA	18.72	-1.43	0.47	-3.07	2.11E-03	2.88E-02
PPDPF	34.88	-1.26	0.41	-3.07	2.16E-03	2.88E-02
RAN	87.19	-0.98	0.32	-3.07	2.15E-03	2.88E-02
SF3B2	67.08	-1.17	0.38	-3.07	2.12E-03	2.88E-02
SLC25A3	56.12	-1.11	0.36	-3.07	2.15E-03	2.88E-02
TMEM183A	131.33	1.02	0.33	3.08	2.08E-03	2.88E-02
TPX2	78.58	-0.85	0.28	-3.07	2.11E-03	2.88E-02
PLEKHJ1	56.67	-1.09	0.36	-3.06	2.21E-03	2.93E-02
ILF3	43.83	-1.14	0.37	-3.06	2.22E-03	2.94E-02
WIPI2	26.87	-1.54	0.5	-3.06	2.24E-03	2.95E-02
FOLR1	50.37	-1.05	0.34	-3.05	2.27E-03	2.96E-02
TIMM50	139.09	-0.97	0.32	-3.05	2.26E-03	2.96E-02
CCT6A	168.08	-1.22	0.4	-3.05	2.29E-03	2.98E-02
RACK1	342.79	-0.71	0.23	-3.04	2.37E-03	3.07E-02
PSMB6	623.81	0.63	0.21	3.04	2.38E-03	3.07E-02
PPAN	15.98	-1.5	0.49	-3.03	2.41E-03	3.08E-02
RAB5IF	57.26	-1.04	0.34	-3.03	2.41E-03	3.08E-02
ASAP1	23.06	-1.33	0.44	-3.03	2.42E-03	3.08E-02
RPLP0	61.92	-1.18	0.39	-3.03	2.48E-03	3.14E-02
MRPS18A	75.4	-0.97	0.32	-3.02	2.50E-03	3.15E-02
YY1	20.78	-1.33	0.44	-3.02	2.50E-03	3.15E-02
C1QBP	104.9	-0.83	0.28	-3.02	2.52E-03	3.15E-02
RPL35A	397.13	0.71	0.23	3.02	2.55E-03	3.18E-02
AC012146.1	35.27	1.05	0.35	3.02	2.56E-03	3.18E-02
COX7B	168.36	0.74	0.25	3.01	2.61E-03	3.23E-02
CIAPIN1	27.5	-1.37	0.46	-2.99	2.79E-03	3.38E-02
CLTA	179.15	-0.92	0.31	-2.99	2.79E-03	3.38E-02
EIF2AK1	59.15	-0.95	0.32	-2.99	2.78E-03	3.38E-02
FTLP2	16.66	-1.42	0.47	-2.99	2.78E-03	3.38E-02
LSM2	61.03	-1.11	0.37	-2.99	2.77E-03	3.38E-02
VTRNA1-3	35.47	1.33	0.45	2.98	2.84E-03	3.43E-02
CMTM6	21.08	-1.48	0.5	-2.98	2.93E-03	3.52E-02

Supplementary Table S4 (Continued). DE genes in Myogel vs. Control.

Genes	*baseMean*	*log2FoldChange*	*lfcSE*	*stat*	*pvalue*	*padj*
LSM7	262.63	0.97	0.33	2.97	2.94E-03	3.52E-02
ITSN1	38.37	-1.3	0.44	-2.97	2.96E-03	3.53E-02
CHCHD10	222.07	-0.81	0.27	-2.97	2.99E-03	3.53E-02
FADS1	64.4	-0.91	0.31	-2.97	2.98E-03	3.53E-02
COPS9	225.57	0.76	0.26	2.97	3.01E-03	3.54E-02
UPF2	126.64	1.01	0.34	2.97	3.01E-03	3.54E-02
SLC39A7	14.22	-1.46	0.49	-2.96	3.09E-03	3.62E-02
NOP10	117.5	0.89	0.3	2.96	3.12E-03	3.64E-02
CDPF1	11.39	-1.55	0.53	-2.95	3.17E-03	3.68E-02
ELAVL1	21.4	-1.29	0.44	-2.95	3.18E-03	3.68E-02
POP4	24.05	-1.24	0.42	-2.95	3.21E-03	3.70E-02
AGPAT2	15.03	-1.47	0.5	-2.94	3.26E-03	3.73E-02
TMEM120A	25.76	-1.16	0.4	-2.94	3.25E-03	3.73E-02
ANKRD36BP1	61.68	-0.86	0.29	-2.94	3.31E-03	3.75E-02
RNASEH1	18.34	-1.32	0.45	-2.94	3.31E-03	3.75E-02
UBL5	100.4	0.77	0.26	2.94	3.32E-03	3.75E-02
TXNL1	11.1	-1.55	0.53	-2.93	3.34E-03	3.76E-02
EPCAM	32.67	-1.17	0.4	-2.93	3.42E-03	3.84E-02
SAC3D1	14	-1.53	0.52	-2.92	3.46E-03	3.86E-02
VGLL4	28.08	-1.13	0.38	-2.92	3.46E-03	3.86E-02
PRMT5	34.76	-1.15	0.4	-2.91	3.56E-03	3.93E-02
PTRH1	120.59	0.73	0.25	2.91	3.56E-03	3.93E-02
MTCH2	19.48	-1.44	0.5	-2.9	3.74E-03	4.09E-02
SELENOK	47.55	0.98	0.34	2.9	3.71E-03	4.09E-02
UQCR11	309.15	0.65	0.23	2.9	3.73E-03	4.09E-02
ELOA	28.52	-1.37	0.47	-2.9	3.76E-03	4.10E-02
TAF7	42.49	-1.12	0.39	-2.9	3.78E-03	4.10E-02
BCS1L	25.17	-1.25	0.43	-2.89	3.81E-03	4.10E-02
RAD23A	40.43	-1.06	0.36	-2.89	3.79E-03	4.10E-02
TRIR	61.43	-0.89	0.31	-2.89	3.89E-03	4.18E-02
NDUFS6	656.83	0.78	0.27	2.89	3.91E-03	4.18E-02
HDDC3	17.49	-1.34	0.46	-2.88	3.99E-03	4.26E-02
RTRAF	50.53	-1.08	0.38	-2.86	4.19E-03	4.45E-02
TNRC6A	20.69	1.34	0.47	2.86	4.28E-03	4.54E-02

Supplementary Table S4 (Continued). DE genes in Myogel vs. Control.

Genes	*baseMean*	*log2FoldChange*	*lfcSE*	*stat*	*pvalue*	*padj*
ECH1	54.14	-0.87	0.3	-2.85	4.37E-03	4.60E-02
TOMM22	23.77	-1.29	0.45	-2.85	4.36E-03	4.60E-02
HNRNPK	137.26	1.05	0.37	2.84	4.45E-03	4.63E-02
RPS4X	137.41	-0.81	0.28	-2.84	4.45E-03	4.63E-02
RTN4	56.66	-1.26	0.44	-2.84	4.45E-03	4.63E-02
C12orf57	97.88	0.8	0.28	2.84	4.51E-03	4.68E-02
EXOSC4	74.35	-0.81	0.28	-2.83	4.63E-03	4.79E-02
COPZ1	48.04	-0.97	0.34	-2.83	4.67E-03	4.80E-02
FARSA	46.74	-1.24	0.44	-2.83	4.66E-03	4.80E-02
SLC25A5	29.09	-1.11	0.39	-2.83	4.68E-03	4.80E-02
RPL10A	66.65	-0.84	0.3	-2.82	4.73E-03	4.83E-02
AC098934.1	259.35	0.82	0.29	2.82	4.85E-03	4.93E-02
EIF5P1	12.64	-1.47	0.52	-2.81	4.93E-03	4.93E-02
EMC3	19.14	-1.28	0.46	-2.81	4.94E-03	4.93E-02
GTF3A	158.29	-0.76	0.27	-2.81	4.91E-03	4.93E-02
LIN28B	81.18	-0.89	0.32	-2.81	4.98E-03	4.93E-02
MKI67	80.99	-0.88	0.31	-2.81	4.96E-03	4.93E-02
PIH1D1	22.71	-1.27	0.45	-2.82	4.86E-03	4.93E-02
PSIP1	71.83	-0.98	0.35	-2.81	4.94E-03	4.93E-02
RTF2	25.74	-1.16	0.41	-2.81	4.91E-03	4.93E-02
SLC25A1	134.14	-0.91	0.32	-2.81	4.99E-03	4.93E-02

Supplementary Table S4 (Continued). DE genes in Myogel vs. Control.

Genes	*baseMean*	*log2FoldChange*	*lfcSE*	*stat*	*pvalue*	*padj*
CST6	108.38	2.47	0.29	8.56	1.10E-17	9.73E-14
S100A16	395.13	1.51	0.22	6.95	3.71E-12	1.63E-08
ITGA2	10.59	2.53	0.44	5.72	1.07E-08	3.15E-05
CLU	53.48	1.48	0.32	4.66	3.21E-06	4.71E-03
DNMT3L	13.99	-1.95	0.42	-4.67	2.98E-06	4.71E-03
SERINC2	31.79	2.03	0.43	4.73	2.28E-06	4.71E-03
DGKD	35.45	1.54	0.34	4.52	6.27E-06	5.82E-03
MYL9	45.23	1.57	0.35	4.51	6.56E-06	5.82E-03
SOX15	26.89	2.02	0.44	4.58	4.72E-06	5.82E-03
TIMP1	24.47	1.95	0.43	4.51	6.60E-06	5.82E-03
GALM	29.94	-1.69	0.38	-4.45	8.63E-06	6.34E-03
HMGCR	51.67	-1.39	0.31	-4.46	8.27E-06	6.34E-03
MBOAT7	50.57	1.49	0.34	4.35	1.39E-05	9.40E-03
CLTB	676.26	1.09	0.25	4.3	1.74E-05	1.02E-02
DHX16	82.99	1.41	0.33	4.3	1.68E-05	1.02E-02
ATMIN	218.42	-1.02	0.24	-4.24	2.22E-05	1.09E-02
FADS1	207.66	-1.14	0.27	-4.26	2.00E-05	1.09E-02
GPAM	16.18	-1.74	0.41	-4.25	2.14E-05	1.09E-02
LINC01356	152.66	-1.1	0.26	-4.21	2.57E-05	1.12E-02
LINC01819	20.77	1.72	0.41	4.2	2.66E-05	1.12E-02
PLIN2	755.09	0.95	0.22	4.22	2.49E-05	1.12E-02
COTL1	150.72	1.47	0.36	4.13	3.62E-05	1.45E-02
EMP2	82.33	-1.21	0.29	-4.1	4.07E-05	1.56E-02
PRPF19	61.63	-1.32	0.32	-4.07	4.63E-05	1.70E-02
KRT8	1334.32	0.91	0.22	4.05	5.16E-05	1.80E-02
TNFRSF12A	163.82	1.15	0.28	4.04	5.31E-05	1.80E-02
CYTOR	27.84	1.39	0.35	3.99	6.62E-05	2.14E-02
TLN1	569.04	-0.87	0.22	-3.98	6.81E-05	2.14E-02
SAT1	40.64	1.51	0.38	3.96	7.38E-05	2.24E-02
KRT18P16	156.19	1.15	0.29	3.91	9.15E-05	2.69E-02
PIK3CB	37.87	-1.35	0.35	-3.86	1.12E-04	3.17E-02
SPHK1	6.61	1.7	0.44	3.86	1.15E-04	3.17E-02
TECR	28.7	-1.43	0.38	-3.82	1.35E-04	3.60E-02
PHLDA1	6.67	1.67	0.44	3.8	1.47E-04	3.81E-02
GADD45G	94.96	-1.18	0.31	-3.78	1.58E-04	3.98E-02

Supplementary Table S5 (Continued). DE genes in Myogel+Fibrin vs. Control.

Genes	*baseMean*	*log2FoldChange*	*lfcSE*	*stat*	*pvalue*	*padj*
TINAGL1	154.93	1.03	0.27	3.77	1.64E-04	4.02E-02
LBR	90.22	-1.14	0.3	-3.76	1.73E-04	4.12E-02
MYLPF	82.48	-1.23	0.33	-3.72	2.02E-04	4.68E-02
SFN	11.89	1.61	0.44	3.71	2.07E-04	4.68E-02
SQLE	144.65	-1.04	0.28	-3.7	2.13E-04	4.68E-02
TRAPPC6A	213.16	-0.94	0.26	-3.7	2.20E-04	4.73E-02
TIMP4	58.03	1.32	0.36	3.68	2.30E-04	4.83E-02
EPB41	112.65	-1.05	0.29	-3.67	2.39E-04	4.91E-02

Supplementary Table S5 (Continued). DE genes in Myogel+Fibrin vs. Control.

Genes	*baseMean*	*log2FoldChange*	*lfcSE*	*stat*	*pvalue*	*padj*
HIST2H2AC	38.29	2.82	0.5	5.61	2.01E-08	1.49E-04
EMILIN2	13.9	2.79	0.56	4.94	7.80E-07	2.68E-03
TTF2	64.26	-1.45	0.3	-4.88	1.09E-06	2.68E-03
SMAP2	14.66	2.54	0.55	4.61	4.11E-06	7.60E-03
CKMT2-AS	17.98	-2.45	0.56	-4.37	1.24E-05	1.83E-02
EPCAM	60.88	1.82	0.43	4.26	2.00E-05	2.46E-02
FAM89A	39.12	1.59	0.38	4.19	2.81E-05	2.97E-02
SP6	24.16	-1.75	0.42	-4.13	3.56E-05	3.29E-02
HIST1H1B	8.89	2.43	0.6	4.06	4.87E-05	3.60E-02
TMEM80	11.09	-2.09	0.52	-4.06	4.83E-05	3.60E-02
HIST1H1E	43.16	1.98	0.5	3.98	6.78E-05	4.29E-02
MTRNR2L1	30.03	-1.8	0.45	-3.97	7.06E-05	4.29E-02
MUS81	7.16	2.48	0.63	3.96	7.54E-05	4.29E-02
EHD4	10.46	2.28	0.58	3.91	9.27E-05	4.88E-02
MRPL28	175.47	1.25	0.32	3.89	9.91E-05	4.88E-02

Supplementary Table S6. DE genes in Myogel+Fibrin vs. Myogel+Fibrin+PDMS.

Genes	*baseMean*	*log2FoldChange*	*lfcSE*	*stat*	*pvalue*	*padj*
PLIN2	323.15	1.54	0.21	7.15	8.82E-13	4.27E-09
HNRNPL	12.91	-2.75	0.51	-5.41	6.29E-08	1.01E-04
TM9SF3	155.05	-2	0.36	-5.48	4.23E-08	1.01E-04
SMARCA5	58.44	-1.6	0.32	-5	5.68E-07	6.87E-04
SMAP2	14.44	-2.39	0.5	-4.78	1.75E-06	1.69E-03
KLHL5	20.28	-2.18	0.46	-4.7	2.61E-06	2.11E-03
NUCKS1	370.42	-1.04	0.22	-4.64	3.43E-06	2.37E-03
KRT18	1010.32	0.99	0.22	4.56	5.02E-06	3.03E-03
KDM5B	166.37	-1.18	0.26	-4.52	6.30E-06	3.38E-03
CTCF	126.4	-1.35	0.3	-4.48	7.34E-06	3.55E-03
AFDN	43.65	-1.58	0.36	-4.42	1.01E-05	4.06E-03
S100A16	185.55	1.31	0.3	4.42	9.91E-06	4.06E-03
ECI1	65.96	-1.57	0.37	-4.3	1.68E-05	6.26E-03
GCM1	13.39	-2.11	0.49	-4.28	1.87E-05	6.44E-03
NOL9	18.47	-1.91	0.46	-4.14	3.52E-05	1.14E-02
KRT18P16	61.27	1.49	0.37	4.08	4.59E-05	1.39E-02
BASP1	75.32	-1.49	0.37	-4.05	5.04E-05	1.43E-02
CENPX	457.19	0.78	0.19	4.02	5.83E-05	1.57E-02
SOX15	7.18	2.1	0.53	3.94	8.31E-05	2.11E-02
SQLE	125.6	-1.09	0.28	-3.91	9.14E-05	2.21E-02
PRDX6	124.65	1.03	0.27	3.83	1.29E-04	2.84E-02
STRN3	18.32	-1.92	0.5	-3.83	1.28E-04	2.84E-02
DOCK7	21.48	-1.82	0.48	-3.81	1.40E-04	2.95E-02
TAGLN	10.97	1.89	0.5	3.76	1.73E-04	3.48E-02
HMGCR	13.07	-1.76	0.47	-3.7	2.13E-04	4.11E-02
DNAJC21	50.69	-1.29	0.35	-3.68	2.31E-04	4.30E-02
ANXA2	429.06	0.71	0.19	3.65	2.65E-04	4.64E-02
NACA3P	190.67	0.85	0.23	3.64	2.69E-04	4.64E-02

Supplementary Table S7. DE genes in Myogel+Fibrin+ PDMS vs. Myogel+PDMS.

Genes	*baseMean*	*log2FoldChange*	*lfcSE*	*stat*	*pvalue*	*padj*
PCYT2	21.7	-1.53	0.42	-3.62	2.98E-04	4.74E-02
URI1	49.15	-1.33	0.37	-3.61	3.04E-04	4.74E-02
WWC2	45.25	-1.25	0.35	-3.62	2.93E-04	4.74E-02
ASAP1	32.25	-1.3	0.36	-3.58	3.44E-04	4.91E-02
KRT8	552.04	0.83	0.23	3.57	3.54E-04	4.91E-02
NFATC2IP	67.75	-1.17	0.33	-3.57	3.61E-04	4.91E-02
OSBP	7.58	-1.87	0.53	-3.56	3.65E-04	4.91E-02
SNX10	299.32	-0.83	0.23	-3.58	3.40E-04	4.91E-02

Supplementary Table S7 (Continued). DE genes in Myogel+Fibrin+ PDMS vs. Myogel+PDMS.

Genes	*baseMean*	*log2FoldChange*	*lfcSE*	*stat*	*pvalue*	*padj*
S100A16	224.67	1.97	0.25	7.88	3.19E-15	1.04E-11
RPS27	549.95	2.07	0.28	7.52	5.48E-14	8.94E-11
COX7C	1233.6	1.51	0.23	6.64	3.18E-11	3.46E-08
CTNNAL1	581.53	1.47	0.23	6.36	2.02E-10	1.32E-07
RPL37A	657.86	1.43	0.22	6.39	1.66E-10	1.32E-07
TPM1	851.18	1.39	0.22	6.31	2.80E-10	1.52E-07
YWHAH	137.63	-2.13	0.34	-6.28	3.48E-10	1.62E-07
LRRC75A	27.58	3	0.51	5.92	3.20E-09	1.04E-06
MT-ND4	330.44	1.35	0.23	5.94	2.93E-09	1.04E-06
SLIRP	1252.81	1.52	0.26	5.95	2.69E-09	1.04E-06
INSIG1	67.18	-2.25	0.38	-5.89	3.96E-09	1.17E-06
RPS20	202	1.61	0.28	5.84	5.12E-09	1.39E-06
ATP5F1E	552.81	1.36	0.24	5.79	7.09E-09	1.41E-06
ATP5ME	1048.93	1.47	0.25	5.8	6.59E-09	1.41E-06
CCNE1	172.58	-1.68	0.29	-5.78	7.31E-09	1.41E-06
KDM5B	213.48	-1.51	0.26	-5.78	7.33E-09	1.41E-06
S100A11	530.03	1.37	0.24	5.79	7.23E-09	1.41E-06
FAM89A	55.41	-2.21	0.39	-5.7	1.21E-08	1.82E-06
GAPDH	752.85	1.44	0.25	5.7	1.23E-08	1.82E-06
RCOR1	172.45	-2.18	0.38	-5.71	1.12E-08	1.82E-06
TDG	204.38	1.55	0.27	5.71	1.14E-08	1.82E-06
TOMM7	682.5	1.27	0.22	5.72	1.08E-08	1.82E-06
MALAT1	505.44	1.62	0.29	5.55	2.82E-08	3.83E-06
RPL38	494.83	1.32	0.24	5.56	2.72E-08	3.83E-06
RPS27AP11	117.11	1.61	0.29	5.52	3.46E-08	4.51E-06
SLC40A1	57.5	-2.1	0.38	-5.49	3.98E-08	4.99E-06
BAG1	73.67	-2.04	0.37	-5.46	4.84E-08	5.84E-06
COX7A2	1508.01	1.39	0.26	5.39	7.00E-08	8.15E-06
UQCR10	2508.22	1.52	0.28	5.35	8.84E-08	9.94E-06
ATP5MPL	1106.89	1.26	0.24	5.32	1.04E-07	1.13E-05
MT-CO3	124.54	1.52	0.29	5.23	1.68E-07	1.76E-05
SNRPE	207.99	1.35	0.26	5.22	1.78E-07	1.81E-05
POMP	723.73	1.33	0.26	5.2	2.01E-07	1.98E-05

Supplementary Table S8. DE genes in Myogel+Fibrin+ PDMS vs.PDMS.

Genes	*baseMean*	*log2FoldChange*	*lfcSE*	*stat*	*pvalue*	*padj*
KRT8	664.92	1.13	0.22	5.18	2.26E-07	2.16E-05
PAPOLA	214.65	-1.42	0.27	-5.17	2.39E-07	2.22E-05
ECI1	74.41	-1.76	0.34	-5.16	2.52E-07	2.28E-05
ATP5MD	490.09	1.17	0.23	5.14	2.73E-07	2.37E-05
NDUFB2	1481.24	1.42	0.28	5.14	2.76E-07	2.37E-05
RPL37	628.26	1.26	0.25	5.13	2.97E-07	2.48E-05
GTSF1	358.05	1.22	0.24	5.11	3.17E-07	2.58E-05
CTCF	158.1	-1.64	0.32	-5.09	3.66E-07	2.91E-05
DDX18	242.42	1.35	0.27	5.06	4.25E-07	3.30E-05
PGF	170.18	-1.42	0.28	-5.04	4.58E-07	3.47E-05
TMA7	247.95	1.46	0.29	5.03	4.85E-07	3.59E-05
AGFG1	153.12	-1.68	0.34	-4.99	6.07E-07	4.36E-05
FAM136A	483.12	1.19	0.24	4.99	6.15E-07	4.36E-05
EIF4E2	286.28	1.15	0.23	4.97	6.66E-07	4.62E-05
SRP14	475.68	1.19	0.24	4.96	7.07E-07	4.71E-05
ZNRD2	753.35	1.19	0.24	4.96	6.99E-07	4.71E-05
ATP5MG	444.03	1.23	0.25	4.95	7.29E-07	4.76E-05
ROMO1	523.47	1.17	0.24	4.94	7.68E-07	4.91E-05
UQCRQ	1686.3	1.29	0.26	4.9	9.63E-07	6.04E-05
COPS9	458.46	1.31	0.27	4.89	9.84E-07	6.05E-05
AC012146.1	70.72	1.67	0.34	4.87	1.10E-06	6.64E-05
COX7B	313.07	1.25	0.26	4.87	1.14E-06	6.74E-05
THOC6	44.25	-2.04	0.42	-4.86	1.16E-06	6.74E-05
MYL6	1926.47	1.25	0.26	4.86	1.19E-06	6.80E-05
WAPL	43.91	-2.02	0.42	-4.85	1.23E-06	6.93E-05
MRPS21	503.82	1.16	0.24	4.84	1.30E-06	7.21E-05
AC098934.1	626.62	1.13	0.23	4.8	1.55E-06	8.43E-05
DNAJC21	69.17	-1.76	0.37	-4.79	1.65E-06	8.79E-05
NDUFA12	645.48	1.21	0.25	4.79	1.67E-06	8.79E-05

Supplementary Table S8 (Continued). DE genes in Myogel+Fibrin+ PDMS vs.PDMS.

Genes	*baseMean*	*log2FoldChange*	*lfcSE*	*stat*	*pvalue*	*padj*
RPS21	440.3	1.04	0.22	4.78	1.75E-06	9.04E-05
SUZ12	32.04	-2.35	0.49	-4.78	1.78E-06	9.06E-05
UBN1	25.84	-2.55	0.54	-4.76	1.96E-06	9.81E-05
XPO1	100.31	-1.68	0.35	-4.74	2.12E-06	1.05E-04
CHORDC1	126.96	1.38	0.29	4.72	2.33E-06	1.13E-04
NDUFA6	1844.56	1.29	0.28	4.7	2.60E-06	1.25E-04
NDUFA1	161.6	1.26	0.27	4.69	2.77E-06	1.31E-04
SMARCA5	79.9	-1.95	0.42	-4.67	3.01E-06	1.40E-04
TOMM5	91.96	1.56	0.34	4.66	3.23E-06	1.49E-04
ENO1	1303.59	1	0.22	4.63	3.59E-06	1.62E-04
SLC9A3R1	64.07	-1.7	0.37	-4.62	3.78E-06	1.69E-04
CYTOR	23.02	2.24	0.48	4.61	3.95E-06	1.74E-04
RPS29	186.61	1.22	0.26	4.61	4.02E-06	1.75E-04
GLRX5	278.49	-1.22	0.26	-4.6	4.19E-06	1.80E-04
MRPS18C	270.42	1.28	0.28	4.59	4.41E-06	1.87E-04
RPS14	4177.67	1.33	0.29	4.59	4.51E-06	1.89E-04
CLTB	224.17	1.22	0.27	4.58	4.64E-06	1.92E-04
ITGB1	662.45	1.21	0.27	4.56	5.23E-06	2.12E-04
PTP4A2	50.07	-1.89	0.42	-4.55	5.26E-06	2.12E-04
MRPL20	653.56	1.07	0.23	4.55	5.33E-06	2.12E-04
LSM3	229.42	1.16	0.25	4.54	5.67E-06	2.20E-04
MEA1	378.08	1.08	0.24	4.54	5.64E-06	2.20E-04
MARCKSL1	35.57	-2.1	0.46	-4.53	5.80E-06	2.23E-04
URI1	64.98	-1.74	0.38	-4.52	6.18E-06	2.34E-04
KRT18	1310.52	0.94	0.21	4.52	6.30E-06	2.36E-04
NOL9	24.92	-2.35	0.52	-4.48	7.52E-06	2.79E-04
AC244090.1	46.56	1.91	0.43	4.46	8.07E-06	2.88E-04
COX6B1	978.7	1.02	0.23	4.47	7.98E-06	2.88E-04
LINC01357	32.66	2.09	0.47	4.46	8.08E-06	2.88E-04
MRPL21	896.44	1.1	0.25	4.46	8.13E-06	2.88E-04
MRPL52	509.21	1.01	0.23	4.45	8.68E-06	3.04E-04
RPL35	3506.89	1.3	0.29	4.44	8.84E-06	3.07E-04
TM9SF3	129.88	-1.7	0.38	-4.43	9.47E-06	3.25E-04
QKI	18.23	-2.66	0.6	-4.42	9.93E-06	3.31E-04

Supplementary Table S8 (Continued). DE genes in Myogel+Fibrin+ PDMS vs.PDMS.

Genes	*baseMean*	*log2FoldChange*	*lfcSE*	*stat*	*pvalue*	*padj*
RPL35A	692	0.97	0.22	4.42	9.77E-06	3.31E-04
VPS29	683.85	1.12	0.25	4.42	9.89E-06	3.31E-04
UFM1	92.83	1.37	0.31	4.41	1.05E-05	3.46E-04
CRIP2	49.45	-1.72	0.39	-4.4	1.10E-05	3.55E-04
TMSB10	1394.83	0.93	0.21	4.4	1.10E-05	3.55E-04
PDCD5	266.38	1.05	0.24	4.38	1.18E-05	3.77E-04
TCERG1	102.47	-1.33	0.3	-4.38	1.19E-05	3.77E-04
RFC3	138.33	1.26	0.29	4.37	1.25E-05	3.93E-04
COMTD1	64.33	-1.61	0.37	-4.36	1.30E-05	4.03E-04
SEPTIN11	226.45	-1.23	0.28	-4.36	1.32E-05	4.07E-04
SEC62	257.21	1.2	0.28	4.34	1.41E-05	4.30E-04
SNRPG	182.06	1.13	0.26	4.32	1.55E-05	4.69E-04
CCND3	88.61	-1.52	0.35	-4.31	1.60E-05	4.78E-04
BOLA3	103.49	1.38	0.32	4.31	1.66E-05	4.86E-04
LIMA1	212.95	1.26	0.29	4.31	1.67E-05	4.86E-04
UQCRH	602.92	1	0.23	4.31	1.66E-05	4.86E-04
CLU	29.11	1.9	0.44	4.29	1.77E-05	5.11E-04
ATP2B1	29.95	-1.98	0.46	-4.28	1.89E-05	5.40E-04
DCXR	413.54	-0.99	0.23	-4.28	1.90E-05	5.40E-04
PEBP1	284.47	-0.99	0.23	-4.26	2.01E-05	5.65E-04
COX20	148.53	1.23	0.29	4.24	2.21E-05	6.16E-04
CDC20	153.25	-1.21	0.29	-4.23	2.33E-05	6.44E-04
DCBLD2	17.67	-2.35	0.56	-4.23	2.38E-05	6.48E-04
STARD10	91.75	-1.35	0.32	-4.23	2.38E-05	6.48E-04
PLIN2	460.67	1.07	0.25	4.22	2.41E-05	6.49E-04
MT-RNR2	4016.74	1.37	0.32	4.22	2.43E-05	6.49E-04
CMSS1	147.93	1.36	0.32	4.2	2.65E-05	7.03E-04
CAV1	43.01	-1.71	0.41	-4.19	2.83E-05	7.43E-04
TMEM258	1025.03	1.17	0.28	4.17	3.10E-05	8.08E-04
SLAIN2	66.1	-1.89	0.46	-4.15	3.35E-05	8.68E-04
NDUFB3	67.3	1.43	0.35	4.12	3.71E-05	9.52E-04
CENPN	314.99	1.06	0.26	4.12	3.79E-05	9.58E-04
YWHAQ	64.93	-1.62	0.39	-4.12	3.77E-05	9.58E-04
MT-CO1	136.16	1.15	0.28	4.12	3.86E-05	9.69E-04

Supplementary Table S8 (Continued). DE genes in Myogel+Fibrin+ PDMS vs.PDMS.

Genes	*baseMean*	*log2FoldChange*	*lfcSE*	*stat*	*pvalue*	*padj*
UBA52	978.98	1.03	0.25	4.11	3.92E-05	9.76E-04
PLEKHF1	59.39	-1.6	0.39	-4.11	4.02E-05	9.88E-04
TTC1	166.1	1.05	0.26	4.11	4.03E-05	9.88E-04
ATP6AP1	131.01	-1.23	0.3	-4.09	4.38E-05	1.07E-03
TXN	119.69	1.22	0.3	4.07	4.73E-05	1.14E-03
UQCRC1	251.73	-1.01	0.25	-4.05	5.21E-05	1.25E-03
MSX2	34.15	-2.03	0.5	-4.04	5.35E-05	1.27E-03
RPF2	127.36	1.21	0.3	4.04	5.40E-05	1.28E-03
RPL23	197.27	1.15	0.29	4.02	5.72E-05	1.34E-03
CCSAP	46.31	-1.82	0.45	-4.01	6.07E-05	1.41E-03
UBL5	198.14	1.19	0.3	4.01	6.08E-05	1.41E-03
VTRNA1-3	61.82	1.59	0.4	4	6.27E-05	1.44E-03
UBE3A	20.1	-2.1	0.53	-3.99	6.59E-05	1.50E-03
RRAGA	49.9	-1.72	0.43	-3.98	6.84E-05	1.55E-03
SEC61G	60.45	1.38	0.35	3.97	7.09E-05	1.59E-03
SVIP	66.75	1.35	0.34	3.97	7.08E-05	1.59E-03
ASAP1	41.47	-1.61	0.41	-3.93	8.33E-05	1.82E-03
ATP5MC1	950.6	1.06	0.27	3.94	8.28E-05	1.82E-03
RPL36AL	401.77	1.03	0.26	3.94	8.22E-05	1.82E-03
NFE2L3	20.19	-2.11	0.54	-3.93	8.67E-05	1.89E-03
CHID1	39.07	-1.72	0.44	-3.92	8.78E-05	1.90E-03
COA3	321.08	0.97	0.25	3.92	8.86E-05	1.90E-03
MTIF3	73.97	1.29	0.33	3.91	9.11E-05	1.92E-03
REEP1	55.94	-1.5	0.38	-3.92	9.04E-05	1.92E-03
SNU13	590.26	0.97	0.25	3.91	9.09E-05	1.92E-03
ELOB	1284.61	1.02	0.26	3.91	9.30E-05	1.95E-03
STRN	34.45	-1.84	0.47	-3.89	9.97E-05	2.07E-03
POLR2I	597	0.95	0.24	3.89	1.01E-04	2.08E-03
RPA3	209.65	0.97	0.25	3.88	1.05E-04	2.15E-03
TMEM208	423.12	0.9	0.23	3.88	1.05E-04	2.15E-03
NANS	58.85	-1.53	0.4	-3.87	1.07E-04	2.17E-03
WDR18	123.46	-1.26	0.33	-3.86	1.14E-04	2.29E-03
AFDN	45.88	-1.56	0.4	-3.85	1.17E-04	2.35E-03
AC010343.1	109.23	1.25	0.32	3.85	1.18E-04	2.35E-03

Supplementary Table S8 (Continued). DE genes in Myogel+Fibrin+ PDMS vs.PDMS.

Genes	*baseMean*	*log2FoldChange*	*lfcSE*	*stat*	*pvalue*	*padj*
RPS19	3723.8	1.1	0.29	3.85	1.19E-04	2.35E-03
MT-RNR1	191.25	1.04	0.27	3.83	1.26E-04	2.48E-03
AIMP1	150.56	1.13	0.3	3.82	1.33E-04	2.57E-03
RPS16P9	262.11	0.92	0.24	3.82	1.33E-04	2.57E-03
SOX4	578.5	1.01	0.26	3.82	1.33E-04	2.57E-03
CENPX	573.42	0.85	0.22	3.81	1.37E-04	2.61E-03
FAM136BP	78.25	1.31	0.34	3.81	1.39E-04	2.61E-03
RPL31	875.91	0.82	0.22	3.81	1.39E-04	2.61E-03
SNHG6	39.62	1.56	0.41	3.81	1.38E-04	2.61E-03
ZFHX2-AS1	41.3	1.81	0.48	3.82	1.36E-04	2.61E-03
FKBP3	223.83	1.03	0.27	3.8	1.47E-04	2.74E-03
SLC25A1	202.49	-1.03	0.27	-3.79	1.52E-04	2.82E-03
GSPT1	262.64	-1.04	0.28	-3.78	1.57E-04	2.90E-03
FMNL2	20.93	-1.93	0.51	-3.77	1.61E-04	2.93E-03
RNF181	555.86	0.94	0.25	3.77	1.60E-04	2.93E-03
RPLP2	1168.62	0.88	0.23	3.76	1.67E-04	3.02E-03
AMDHD2	50.03	-1.52	0.41	-3.76	1.68E-04	3.04E-03
GRB2	161.27	-1.14	0.3	-3.76	1.71E-04	3.07E-03
SENP6	38.19	-1.82	0.49	-3.73	1.88E-04	3.35E-03
CST6	41.69	1.84	0.49	3.73	1.94E-04	3.43E-03
BLOC1S4	73.64	-1.37	0.37	-3.72	1.98E-04	3.47E-03
COX6C	74.32	1.29	0.35	3.72	1.98E-04	3.47E-03
KRT10	336.62	0.98	0.26	3.71	2.06E-04	3.58E-03
GADD45G	54.74	-1.49	0.4	-3.71	2.09E-04	3.60E-03
MNAT1	125.81	1.2	0.32	3.71	2.08E-04	3.60E-03
ANAPC11	730.3	0.92	0.25	3.7	2.14E-04	3.67E-03
ROGDI	29.06	-1.69	0.46	-3.69	2.23E-04	3.76E-03
SECISBP2	56.02	-1.47	0.4	-3.69	2.22E-04	3.76E-03
TMEM165	87.84	-1.25	0.34	-3.69	2.21E-04	3.76E-03
RABIF	388.9	0.92	0.25	3.68	2.31E-04	3.88E-03
NAP1L1	99.63	1.08	0.29	3.68	2.35E-04	3.94E-03
HSBP1	496.91	0.87	0.24	3.66	2.48E-04	4.12E-03
VAMP8	309.29	0.89	0.24	3.66	2.53E-04	4.19E-03
TLK1	32.15	-1.63	0.45	-3.66	2.56E-04	4.22E-03

Supplementary Table S8 (Continued). DE genes in Myogel+Fibrin+ PDMS vs.PDMS.

Genes	*baseMean*	*log2FoldChange*	*lfcSE*	*stat*	*pvalue*	*padj*
NDUFA2	464.52	0.89	0.24	3.65	2.59E-04	4.23E-03
WAC	54.71	-1.5	0.41	-3.65	2.59E-04	4.23E-03
SPIN1	43.5	-1.55	0.43	-3.65	2.61E-04	4.24E-03
REV1	46.2	-1.46	0.4	-3.65	2.64E-04	4.27E-03
CISD3	304.5	0.96	0.26	3.64	2.70E-04	4.34E-03
FAM120AOS	68.52	-1.42	0.39	-3.64	2.75E-04	4.38E-03
S100P	85.64	1.24	0.34	3.64	2.76E-04	4.38E-03
SCNM1	123.13	1.04	0.29	3.64	2.77E-04	4.39E-03
BRI3	39.47	-1.47	0.41	-3.62	3.00E-04	4.72E-03
YBX1	115.67	-1.11	0.31	-3.61	3.01E-04	4.72E-03
MRPL22	334.94	0.97	0.27	3.61	3.11E-04	4.84E-03
MT-ND3	22.78	1.87	0.52	3.61	3.12E-04	4.84E-03
SNAP47	79.78	1.31	0.36	3.6	3.20E-04	4.94E-03
TFAP2C	36.58	-1.71	0.48	-3.59	3.31E-04	5.09E-03
MT-CYB	115.58	1.2	0.33	3.59	3.34E-04	5.11E-03
DMKN	90.87	1.21	0.34	3.58	3.45E-04	5.23E-03
KRT18P16	81.68	1.41	0.39	3.58	3.45E-04	5.23E-03
VGLL4	57.56	-1.45	0.41	-3.57	3.56E-04	5.38E-03
FTL	2391.68	0.91	0.26	3.57	3.61E-04	5.42E-03
HNRNPD	67.64	-1.22	0.34	-3.57	3.63E-04	5.43E-03
AVPI1	25.52	-1.7	0.48	-3.56	3.68E-04	5.48E-03
TBC1D20	30.51	-1.6	0.45	-3.56	3.70E-04	5.48E-03
RPL24	110.37	1.04	0.29	3.56	3.73E-04	5.51E-03
WIPI1	23.1	-1.79	0.5	-3.55	3.79E-04	5.57E-03
MRPL41	1881.34	0.97	0.27	3.54	3.97E-04	5.80E-03
ISG15	317.1	0.84	0.24	3.54	3.99E-04	5.80E-03
RBBP7	51.68	-1.42	0.4	-3.54	4.04E-04	5.86E-03
SON	313.09	1.09	0.31	3.53	4.10E-04	5.92E-03
RMDN1	45.88	1.38	0.39	3.53	4.15E-04	5.94E-03
VPS4B	113.28	1.15	0.33	3.53	4.15E-04	5.94E-03
CBX1	170.61	1.03	0.29	3.53	4.21E-04	5.99E-03
NEDD8	940.06	0.91	0.26	3.52	4.29E-04	6.09E-03
KTN1	356.25	0.83	0.24	3.52	4.35E-04	6.14E-03
NDUFA13	428.79	0.84	0.24	3.51	4.55E-04	6.37E-03

Supplementary Table S8 (Continued). DE genes in Myogel+Fibrin+ PDMS vs.PDMS.

Genes	*baseMean*	*log2FoldChange*	*lfcSE*	*stat*	*pvalue*	*padj*
SNRPD2	376.7	0.84	0.24	3.51	4.55E-04	6.37E-03
RPS16	1424.55	0.85	0.24	3.5	4.59E-04	6.39E-03
SPPL2A	19.63	-1.96	0.56	-3.5	4.71E-04	6.53E-03
VWA1	88.36	-1.12	0.32	-3.48	4.93E-04	6.82E-03
VPS36	146.13	1.1	0.32	3.48	4.97E-04	6.83E-03
PTTG1	184.25	0.88	0.25	3.48	5.03E-04	6.90E-03
BASP1	71.64	-1.3	0.37	-3.47	5.23E-04	7.07E-03
CEP131	17.24	-1.88	0.54	-3.47	5.22E-04	7.07E-03
POLR2L	326.58	0.79	0.23	3.47	5.23E-04	7.07E-03
BRD4	33.45	-1.69	0.49	-3.47	5.29E-04	7.13E-03
SMARCC1	68.34	-1.25	0.36	-3.46	5.38E-04	7.22E-03
ATXN10	40.61	-1.49	0.43	-3.46	5.46E-04	7.25E-03
KNOP1	280.62	0.94	0.27	3.46	5.48E-04	7.25E-03
MRPL37	175.53	-1.08	0.31	-3.45	5.51E-04	7.25E-03
NDUFA4	342.78	0.83	0.24	3.46	5.46E-04	7.25E-03
NDUFAB1	536.39	0.86	0.25	3.46	5.50E-04	7.25E-03
TXNDC17	222.98	0.98	0.28	3.45	5.68E-04	7.44E-03
SNX17	43.19	-1.39	0.41	-3.42	6.18E-04	8.06E-03
PCBP1	50.97	-1.43	0.42	-3.42	6.27E-04	8.14E-03
TIMM10	278.02	0.88	0.26	3.42	6.32E-04	8.18E-03
RPL27	693.91	0.86	0.25	3.41	6.41E-04	8.27E-03
BTG1	31.06	-1.55	0.45	-3.41	6.54E-04	8.39E-03
EFHD2	98.77	-1.25	0.37	-3.39	6.91E-04	8.79E-03
MKI67	121.69	-1.11	0.33	-3.39	6.89E-04	8.79E-03
SEM1	92.13	1.06	0.31	3.39	6.93E-04	8.79E-03
MRPS10	187.04	0.96	0.28	3.39	6.98E-04	8.82E-03
NAA38	284.88	0.87	0.26	3.39	7.07E-04	8.90E-03
SLC25A4	69.92	-1.33	0.39	-3.38	7.24E-04	9.09E-03
RPL41	383.63	0.95	0.28	3.38	7.36E-04	9.20E-03
TAF1D	59.21	1.18	0.35	3.37	7.40E-04	9.20E-03
CUL4A	56.47	-1.22	0.36	-3.36	7.74E-04	9.56E-03
U2SURP	256.18	0.93	0.28	3.36	7.72E-04	9.56E-03
NDUFS5	379.48	0.83	0.25	3.36	7.78E-04	9.58E-03
FADS3	34.56	-1.57	0.47	-3.36	7.90E-04	9.69E-03

Supplementary Table S8 (Continued). DE genes in Myogel+Fibrin+ PDMS vs.PDMS.

Genes	*baseMean*	*log2FoldChange*	*lfcSE*	*stat*	*pvalue*	*padj*
PHPT1	2030.58	0.93	0.28	3.35	7.98E-04	9.74E-03
ARF6	23.44	-1.69	0.5	-3.35	8.11E-04	9.87E-03
RPL15	808.08	0.67	0.2	3.35	8.16E-04	9.90E-03
MRPL33	80.36	1.17	0.35	3.34	8.25E-04	9.97E-03
SREK1IP1	54.08	1.22	0.37	3.34	8.40E-04	1.01E-02
AHCY	45.1	1.39	0.42	3.33	8.59E-04	1.03E-02
CSNK2B	137.4	0.91	0.28	3.32	8.93E-04	1.07E-02
GNL3	256.97	0.83	0.25	3.32	8.99E-04	1.07E-02
ZNRF1	36.53	-1.41	0.42	-3.32	9.03E-04	1.07E-02
C6orf132	29.38	1.49	0.45	3.31	9.37E-04	1.10E-02
DYNC1H1	43.35	-1.3	0.39	-3.31	9.31E-04	1.10E-02
GARS	33.71	-1.45	0.44	-3.31	9.44E-04	1.10E-02
PDIA3	61.54	-1.34	0.4	-3.31	9.46E-04	1.10E-02
POLE3	92.12	1.07	0.32	3.31	9.45E-04	1.10E-02
SRSF9	74.5	-1.12	0.34	-3.31	9.48E-04	1.10E-02
XBP1	65.28	-1.21	0.37	-3.31	9.49E-04	1.10E-02
RPS23	588.84	0.75	0.23	3.3	9.67E-04	1.11E-02
CHURC1	50.59	1.2	0.37	3.29	9.91E-04	1.14E-02
HINT1	681.64	0.78	0.24	3.29	1.00E-03	1.14E-02
PCYT2	23.5	-1.62	0.49	-3.29	1.00E-03	1.14E-02
DPM3	65.28	1.12	0.34	3.27	1.08E-03	1.23E-02
RPS25	116.34	1.01	0.31	3.27	1.09E-03	1.23E-02
UQCC2	358.06	0.86	0.26	3.27	1.09E-03	1.23E-02
PSENEN	66.05	1.13	0.35	3.26	1.11E-03	1.25E-02
CCDC167	334.28	0.89	0.27	3.26	1.13E-03	1.27E-02
STMN1	304.15	0.76	0.23	3.25	1.13E-03	1.27E-02
CDC34	31.85	-1.43	0.44	-3.25	1.14E-03	1.27E-02
CEBPD	49.14	-1.37	0.42	-3.25	1.17E-03	1.29E-02
SLC25A3	87.46	-1.08	0.33	-3.25	1.17E-03	1.29E-02
SMIM26	86.76	1.03	0.32	3.24	1.21E-03	1.33E-02
GLO1	152.69	0.96	0.3	3.24	1.22E-03	1.33E-02
UBB	905.25	0.7	0.22	3.23	1.22E-03	1.33E-02
SUPT20H	30.35	-1.47	0.45	-3.23	1.22E-03	1.34E-02
KDM3B	55.27	-1.32	0.41	-3.22	1.26E-03	1.37E-02

Supplementary Table S8 (Continued). DE genes in Myogel+Fibrin+ PDMS vs.PDMS.

Genes	*baseMean*	*log2FoldChange*	*lfcSE*	*stat*	*pvalue*	*padj*
CEBPB	25.31	-1.55	0.48	-3.22	1.28E-03	1.39E-02
CYBA	122.26	-1.07	0.33	-3.21	1.31E-03	1.40E-02
DGKD	24.03	1.53	0.47	3.21	1.31E-03	1.40E-02
EIF2AK1	122.93	-1.01	0.31	-3.21	1.31E-03	1.40E-02
KDELR2	66.69	-1.15	0.36	-3.22	1.30E-03	1.40E-02
APPL1	173.61	0.89	0.28	3.21	1.34E-03	1.43E-02
ENDOG	17.68	-1.77	0.55	-3.2	1.37E-03	1.45E-02
SIGIRR	28.64	-1.6	0.5	-3.2	1.37E-03	1.45E-02
NUP62	77.96	-1.1	0.34	-3.19	1.42E-03	1.50E-02
DDX21	267.18	0.88	0.28	3.19	1.44E-03	1.51E-02
MT-CO2	1413.92	0.87	0.27	3.19	1.45E-03	1.51E-02
PPP2CA	42.25	-1.37	0.43	-3.19	1.44E-03	1.51E-02
DOCK7	19.23	-1.75	0.55	-3.18	1.48E-03	1.54E-02
MLF2	131.15	-1.03	0.32	-3.18	1.48E-03	1.54E-02
LSM7	521.81	0.81	0.26	3.17	1.54E-03	1.60E-02
SMARCA4	101.49	-1.12	0.35	-3.16	1.57E-03	1.62E-02
STRN3	17.57	-1.88	0.6	-3.16	1.59E-03	1.63E-02
B2M	181.58	0.94	0.3	3.15	1.61E-03	1.64E-02
MT2A	22.72	1.5	0.48	3.16	1.60E-03	1.64E-02
TTF2	84.07	1.01	0.32	3.15	1.61E-03	1.64E-02
UBTF	37.54	-1.33	0.42	-3.15	1.63E-03	1.65E-02
BEX4	27.52	-1.46	0.46	-3.15	1.64E-03	1.65E-02
PBRM1	52.64	-1.19	0.38	-3.15	1.63E-03	1.65E-02
PTN	66.72	1.11	0.35	3.15	1.64E-03	1.65E-02
KRT7	147.45	0.98	0.31	3.14	1.68E-03	1.68E-02
CCND1	200.45	0.81	0.26	3.14	1.68E-03	1.68E-02
FAU	806.82	0.66	0.21	3.14	1.69E-03	1.68E-02
NSD3	171.21	-0.83	0.27	-3.13	1.74E-03	1.73E-02
C1orf109	36.46	1.37	0.44	3.13	1.76E-03	1.73E-02
MDM2	18.26	-1.65	0.53	-3.13	1.76E-03	1.73E-02
PPDPF	51.01	-1.2	0.38	-3.13	1.76E-03	1.73E-02
RAB7A	35.5	-1.39	0.45	-3.12	1.80E-03	1.77E-02
MCUR1	67.8	-1.06	0.34	-3.11	1.86E-03	1.81E-02
NDUFAF2	93.52	0.97	0.31	3.11	1.85E-03	1.81E-02

Supplementary Table S8 (Continued). DE genes in Myogel+Fibrin+ PDMS vs.PDMS.

Genes	*baseMean*	*log2FoldChange*	*lfcSE*	*stat*	*pvalue*	*padj*
NUDT14	22.86	-1.68	0.54	-3.11	1.88E-03	1.83E-02
CIB1	132.73	0.9	0.29	3.11	1.89E-03	1.84E-02
ABHD12	30.8	-1.46	0.47	-3.1	1.92E-03	1.86E-02
INF2	42.78	1.36	0.44	3.1	1.92E-03	1.86E-02
EIF5B	297.65	0.88	0.28	3.1	1.94E-03	1.86E-02
PSMB1	680.85	0.72	0.23	3.09	1.98E-03	1.90E-02
LINC00467	25.78	1.41	0.46	3.09	1.98E-03	1.90E-02
DYNC1I2	48.63	1.19	0.38	3.09	2.01E-03	1.92E-02
ATMIN	97.3	-1.32	0.43	-3.08	2.05E-03	1.94E-02
CDKN3	160.4	0.84	0.27	3.08	2.04E-03	1.94E-02
RPL34	377.15	0.72	0.23	3.08	2.09E-03	1.98E-02
TAF11	104.09	1.04	0.34	3.07	2.15E-03	2.03E-02
COX5B	1982.74	0.79	0.26	3.06	2.18E-03	2.05E-02
TP53RK	73.05	-1.06	0.35	-3.06	2.24E-03	2.09E-02
SBNO1	42.72	-1.26	0.41	-3.05	2.28E-03	2.13E-02
RAB11FIP1	193.95	-1.04	0.34	-3.05	2.31E-03	2.15E-02
CHTOP	53.32	-1.15	0.38	-3.05	2.32E-03	2.16E-02
PHLDA3	25.53	-1.45	0.48	-3.04	2.34E-03	2.16E-02
RPS24	200.51	0.79	0.26	3.04	2.36E-03	2.17E-02
TINAGL1	61.01	1.08	0.36	3.04	2.36E-03	2.17E-02
FAM133B	56.82	1.14	0.38	3.04	2.39E-03	2.20E-02
RPS18	1423.94	0.66	0.22	3.03	2.42E-03	2.21E-02
SLC29A1	47.79	-1.19	0.39	-3.03	2.44E-03	2.22E-02
TMEM203	58.73	-1.13	0.37	-3.03	2.45E-03	2.23E-02
NDUFS6	1148.29	0.73	0.24	3.03	2.47E-03	2.24E-02
RTN2	23.81	-1.49	0.49	-3.03	2.47E-03	2.24E-02
SVIL	24.07	1.5	0.5	3.02	2.49E-03	2.25E-02
GOLM1	28.53	-1.43	0.47	-3.02	2.52E-03	2.27E-02
AC087752.1	26.27	1.39	0.46	3.02	2.57E-03	2.29E-02
AKR7A2	33.87	-1.39	0.46	-3.02	2.55E-03	2.29E-02
CALM1	1907.68	0.79	0.26	3.02	2.56E-03	2.29E-02
RPLP1	1698.93	0.68	0.23	3.02	2.55E-03	2.29E-02
RRP15	52.26	1.12	0.37	3.01	2.59E-03	2.30E-02
AKIP1	58.36	1.04	0.35	3.01	2.64E-03	2.34E-02

Supplementary Table S8 (Continued). DE genes in Myogel+Fibrin+ PDMS vs.PDMS.

Genes	*baseMean*	*log2FoldChange*	*lfcSE*	*stat*	*pvalue*	*padj*
APOE	23.67	-1.57	0.52	-3	2.69E-03	2.36E-02
DAD1	361.65	0.73	0.24	3	2.70E-03	2.36E-02
EHMT1	106.43	-0.9	0.3	-3	2.70E-03	2.36E-02
MED27	36.43	-1.29	0.43	-3	2.70E-03	2.36E-02
MRPL28	155.79	-0.93	0.31	-3	2.69E-03	2.36E-02
MRPS5	599.37	0.8	0.27	3	2.70E-03	2.36E-02
POLR2G	104.23	0.92	0.31	3	2.72E-03	2.36E-02
PGRMC2	17.71	-1.58	0.53	-3	2.74E-03	2.37E-02
SPG21	19.28	-1.59	0.53	-3	2.73E-03	2.37E-02
SCAND1	227.92	-0.95	0.32	-2.99	2.75E-03	2.37E-02
SET	184.01	-0.89	0.3	-2.99	2.76E-03	2.37E-02
ZSWIM7	97.39	0.9	0.3	2.99	2.76E-03	2.37E-02
AKIRIN1	31.38	-1.47	0.49	-2.99	2.79E-03	2.38E-02
NSA2	114.09	0.91	0.31	2.99	2.78E-03	2.38E-02
FNBP1	21.35	-1.5	0.5	-2.99	2.81E-03	2.38E-02
SORT1	22.01	-1.6	0.53	-2.99	2.80E-03	2.38E-02
RPS8	691.34	0.67	0.23	2.98	2.84E-03	2.41E-02
KRT18P3	37.94	1.28	0.43	2.98	2.86E-03	2.41E-02
HIST1H1E	30.71	-1.49	0.5	-2.98	2.90E-03	2.44E-02
TINCR	55.31	-1.08	0.36	-2.98	2.92E-03	2.45E-02
SERF2	1308.36	0.8	0.27	2.97	2.99E-03	2.51E-02
DENR	138.91	0.97	0.33	2.96	3.06E-03	2.56E-02
OGA	36.9	-1.33	0.45	-2.96	3.09E-03	2.58E-02
NACA3P	249.48	0.71	0.24	2.96	3.10E-03	2.58E-02
EIF2B1	29.86	-1.31	0.45	-2.95	3.16E-03	2.62E-02
PSMA7	1341.38	0.63	0.21	2.95	3.16E-03	2.62E-02
SH3GLB2	23.36	-1.45	0.49	-2.95	3.18E-03	2.63E-02
FST	19.91	-1.55	0.53	-2.95	3.21E-03	2.64E-02
ARPC5	216.14	0.84	0.28	2.95	3.22E-03	2.64E-02
TMA16	130.75	0.9	0.3	2.95	3.23E-03	2.64E-02
MFAP5	24.98	1.41	0.48	2.94	3.24E-03	2.65E-02
NFKBIA	28.09	-1.37	0.47	-2.94	3.27E-03	2.66E-02
METAP2	145.6	0.84	0.29	2.94	3.29E-03	2.67E-02
TMED2	78.67	-1.04	0.35	-2.94	3.30E-03	2.67E-02

Supplementary Table S8 (Continued). DE genes in Myogel+Fibrin+ PDMS vs.PDMS.

Genes	*baseMean*	*log2FoldChange*	*lfcSE*	*stat*	*pvalue*	*padj*
VKORC1	295.08	0.81	0.27	2.94	3.30E-03	2.67E-02
ISOC1	21.46	-1.49	0.51	-2.93	3.35E-03	2.71E-02
NOP10	215.02	0.88	0.3	2.93	3.41E-03	2.74E-02
XAGE3	67.35	1	0.34	2.92	3.46E-03	2.78E-02
AC015871.1	33.34	1.34	0.46	2.92	3.48E-03	2.79E-02
DDX52	73.51	0.97	0.33	2.92	3.52E-03	2.81E-02
PUM3	193.04	0.9	0.31	2.92	3.52E-03	2.81E-02
CENPW	141.44	0.81	0.28	2.92	3.54E-03	2.82E-02
RAB10	88.39	-1.14	0.39	-2.92	3.55E-03	2.82E-02
SAE1	59.84	-1.08	0.37	-2.91	3.58E-03	2.83E-02
RMI2	56.2	-1.24	0.43	-2.91	3.62E-03	2.86E-02
MICOS10	143.69	0.91	0.31	2.91	3.66E-03	2.88E-02
PAXIP1-AS1	19.09	1.56	0.54	2.91	3.65E-03	2.88E-02
EIF3F	30.7	-1.42	0.49	-2.9	3.69E-03	2.88E-02
NME2	86.15	1.02	0.35	2.9	3.70E-03	2.88E-02
TRAPPC2L	624.18	0.64	0.22	2.9	3.68E-03	2.88E-02
ATP2B4	63.41	-1.09	0.38	-2.9	3.71E-03	2.88E-02
TSEN34	59.23	-1.07	0.37	-2.9	3.71E-03	2.88E-02
RPS13	754.53	0.6	0.21	2.89	3.80E-03	2.94E-02
ZCRB1	60.43	1.06	0.37	2.89	3.81E-03	2.95E-02
CIAO2B	321.82	0.81	0.28	2.89	3.84E-03	2.96E-02
RBMX	136.9	0.8	0.28	2.89	3.88E-03	2.99E-02
CCDC47	103.4	-0.92	0.32	-2.89	3.90E-03	3.00E-02
TNIP2	69.91	-1.11	0.38	-2.88	3.94E-03	3.02E-02
RAI14	35.23	-1.21	0.42	-2.88	3.97E-03	3.03E-02
ATP5PD	160.67	0.82	0.29	2.88	4.01E-03	3.05E-02
PPP2R5E	23.87	-1.41	0.49	-2.87	4.06E-03	3.09E-02
MXRA8	55.51	-1.06	0.37	-2.87	4.08E-03	3.10E-02
UPF2	232.58	0.94	0.33	2.87	4.12E-03	3.12E-02
RPS11	471.63	0.64	0.22	2.86	4.20E-03	3.17E-02
CHCHD10	345.3	-0.77	0.27	-2.86	4.21E-03	3.17E-02
PDCD10	59.28	1	0.35	2.86	4.23E-03	3.18E-02
OST4	376.22	0.74	0.26	2.85	4.36E-03	3.27E-02
CENPP	24.65	-1.34	0.47	-2.84	4.45E-03	3.29E-02

Supplementary Table S8 (Continued). DE genes in Myogel+Fibrin+ PDMS vs.PDMS.

Genes	*baseMean*	*log2FoldChange*	*lfcSE*	*stat*	*pvalue*	*padj*
CPVL	57.23	-1.07	0.38	-2.85	4.41E-03	3.29E-02
EMC8	53.08	-1.15	0.4	-2.84	4.44E-03	3.29E-02
FDFT1	44.08	-1.13	0.4	-2.85	4.43E-03	3.29E-02
NDUFAF8	406.5	0.73	0.26	2.85	4.43E-03	3.29E-02
SLC2A1	39.05	-1.16	0.41	-2.84	4.45E-03	3.29E-02
MMP24OS	27.69	-1.3	0.46	-2.84	4.49E-03	3.31E-02
CBX3	405.87	0.78	0.28	2.84	4.56E-03	3.36E-02
PPP1R35	58.9	1.1	0.39	2.83	4.63E-03	3.40E-02
TPM4	57.29	-1.07	0.38	-2.83	4.68E-03	3.43E-02
MTRNR2L1	39.44	1.24	0.44	2.83	4.72E-03	3.45E-02
MRPL1	80.88	0.97	0.35	2.82	4.74E-03	3.46E-02
HMGB1	66.24	0.97	0.34	2.82	4.77E-03	3.47E-02
ATP5PF	573.13	0.75	0.27	2.82	4.84E-03	3.52E-02
ADIPOR1	69.78	-0.98	0.35	-2.81	4.88E-03	3.53E-02
NUDT16L1	41.56	-1.17	0.41	-2.81	4.88E-03	3.53E-02
CERT1	69.33	-1.04	0.37	-2.81	4.92E-03	3.55E-02
LAIR2	41.14	1.15	0.41	2.81	4.93E-03	3.55E-02
NMT1	52.12	1.04	0.37	2.8	5.10E-03	3.66E-02
TOMM40	52.01	-1.06	0.38	-2.8	5.12E-03	3.67E-02
MTCH1	169.28	-0.86	0.31	-2.8	5.15E-03	3.69E-02
MPST	60.58	-1.06	0.38	-2.8	5.17E-03	3.69E-02
MBNL3	106.1	-0.94	0.34	-2.79	5.20E-03	3.70E-02
DDX17	26.66	-1.33	0.48	-2.79	5.24E-03	3.71E-02
MORF4L2	31.99	-1.28	0.46	-2.79	5.25E-03	3.71E-02
RNF145	41.17	1.13	0.4	2.79	5.23E-03	3.71E-02
LMO4	36.55	-1.35	0.49	-2.79	5.34E-03	3.77E-02
NDUFA10	63.54	-1.07	0.38	-2.78	5.41E-03	3.81E-02
PSIP1	116.18	-0.9	0.32	-2.78	5.45E-03	3.83E-02
POLR2J	348.33	0.72	0.26	2.78	5.51E-03	3.85E-02
RAB1A	39.59	-1.16	0.42	-2.78	5.50E-03	3.85E-02
CHD1	68.54	-0.99	0.36	-2.77	5.53E-03	3.86E-02
BOD1	19.12	-1.48	0.54	-2.77	5.58E-03	3.88E-02
SPINT2	63.79	-1	0.36	-2.77	5.58E-03	3.88E-02
CCNA2	30.47	-1.39	0.5	-2.77	5.67E-03	3.92E-02

Supplementary Table S8 (Continued). DE genes in Myogel+Fibrin+ PDMS vs.PDMS.

Genes	*baseMean*	*log2FoldChange*	*lfcSE*	*stat*	*pvalue*	*padj*
FAM98A	114.61	0.93	0.33	2.77	5.67E-03	3.92E-02
LAMTOR5	264.7	0.81	0.29	2.77	5.67E-03	3.92E-02
NRP2	22.37	1.37	0.5	2.77	5.69E-03	3.92E-02
PSME2	289.62	0.7	0.25	2.77	5.69E-03	3.92E-02
SSB	571.52	0.77	0.28	2.76	5.72E-03	3.93E-02
ABHD11	52.96	-1.08	0.39	-2.76	5.77E-03	3.96E-02
PWWP2A	48.58	-1.13	0.41	-2.76	5.81E-03	3.97E-02
RPS27A	381.61	0.62	0.23	2.76	5.83E-03	3.97E-02
UBE2B	92.92	-0.9	0.33	-2.76	5.84E-03	3.97E-02
MVB12A	46.05	-1.15	0.42	-2.75	5.93E-03	4.02E-02
NSMCE1	62.98	0.99	0.36	2.75	5.92E-03	4.02E-02
FKBP4	79.97	0.9	0.33	2.75	5.94E-03	4.02E-02
EIF1	484.48	0.65	0.24	2.75	5.96E-03	4.02E-02
HDHD5	24.92	-1.34	0.49	-2.75	5.98E-03	4.03E-02
SNX4	17.34	-1.47	0.53	-2.74	6.11E-03	4.11E-02
RPP21	26.05	1.27	0.46	2.74	6.12E-03	4.11E-02
AL136454.1	32.2	1.17	0.43	2.74	6.19E-03	4.14E-02
ATOX1	538.99	0.65	0.24	2.74	6.24E-03	4.14E-02
EIF3K	490.26	0.64	0.23	2.74	6.21E-03	4.14E-02
ITSN1	52.18	-1.07	0.39	-2.74	6.21E-03	4.14E-02
NDUFB7	878.32	0.65	0.24	2.74	6.23E-03	4.14E-02
CD47	59.62	-1.07	0.39	-2.73	6.33E-03	4.19E-02
RBIS	38.61	1.12	0.41	2.73	6.42E-03	4.24E-02
FTH1P11	90.56	-0.92	0.34	-2.72	6.46E-03	4.26E-02
HNRNPU	238.94	-0.73	0.27	-2.72	6.48E-03	4.26E-02
MT-ATP6	64.68	1	0.37	2.72	6.46E-03	4.26E-02
RBX1	541.9	0.68	0.25	2.72	6.52E-03	4.28E-02
NINJ1	125.92	-0.87	0.32	-2.72	6.53E-03	4.28E-02
NHP2	1118.64	0.67	0.25	2.72	6.55E-03	4.28E-02
MED15	95.39	1.02	0.38	2.72	6.57E-03	4.28E-02
MRPS16	479.42	0.7	0.26	2.72	6.59E-03	4.29E-02
ACTN1	72.48	0.98	0.36	2.71	6.69E-03	4.35E-02
VAPA	86.94	-0.96	0.35	-2.71	6.78E-03	4.40E-02
UQCR11	506.36	0.69	0.26	2.7	6.91E-03	4.47E-02

Supplementary Table S8 (Continued). DE genes in Myogel+Fibrin+ PDMS vs.PDMS.

Genes	*baseMean*	*log2FoldChange*	*lfcSE*	*stat*	*pvalue*	*padj*
ALG3	95.31	-0.89	0.33	-2.7	6.94E-03	4.48E-02
ACVR2B	17.17	1.47	0.54	2.7	6.99E-03	4.49E-02
LSM5	169.39	0.78	0.29	2.7	6.96E-03	4.49E-02
TIMM8B	246.32	0.77	0.28	2.7	6.99E-03	4.49E-02
MVD	40.68	-1.15	0.43	-2.69	7.10E-03	4.55E-02
NSUN6	18.03	1.42	0.53	2.69	7.12E-03	4.55E-02
ADIPOR2	29.89	-1.36	0.5	-2.69	7.15E-03	4.55E-02
ATP5IF1	544.49	0.64	0.24	2.69	7.14E-03	4.55E-02
EIF2S2	310.36	0.75	0.28	2.69	7.22E-03	4.56E-02
NDUFA8	299.8	0.72	0.27	2.69	7.22E-03	4.56E-02
PFN1	571.94	0.68	0.25	2.69	7.21E-03	4.56E-02
SNRPC	239.25	0.74	0.28	2.69	7.21E-03	4.56E-02
RPS27L	275.54	0.67	0.25	2.69	7.25E-03	4.57E-02
MAGOHB	58.42	0.94	0.35	2.68	7.34E-03	4.60E-02
PFDN4	39.28	1.14	0.43	2.68	7.34E-03	4.60E-02
ZMAT5	84.71	0.9	0.33	2.68	7.32E-03	4.60E-02
DCTN2	34.1	1.17	0.44	2.68	7.39E-03	4.62E-02
FUCA2	51.4	-1.24	0.46	-2.68	7.44E-03	4.63E-02
NDUFV3	362.79	0.69	0.26	2.68	7.43E-03	4.63E-02
RNF111	18.71	-1.47	0.55	-2.68	7.44E-03	4.63E-02
AL356441.2	100.78	0.94	0.35	2.67	7.54E-03	4.67E-02
COMMD7	98.27	-0.97	0.36	-2.67	7.52E-03	4.67E-02
SDF2L1	984.04	0.65	0.24	2.67	7.53E-03	4.67E-02
KIF2A	58.34	-0.96	0.36	-2.67	7.62E-03	4.70E-02
PA2G4	346	0.71	0.27	2.67	7.60E-03	4.70E-02
USP48	70.63	-1	0.38	-2.67	7.63E-03	4.70E-02
CLTA	337.52	-0.74	0.28	-2.67	7.68E-03	4.71E-02
PSMC3IP	17.22	1.45	0.54	2.66	7.76E-03	4.76E-02
CNOT11	17.33	-1.4	0.53	-2.66	7.93E-03	4.82E-02
DDX49	47	1.08	0.41	2.66	7.90E-03	4.82E-02
DPY30	85.67	0.84	0.32	2.66	7.89E-03	4.82E-02
MRPL24	306.44	0.73	0.27	2.65	7.95E-03	4.82E-02
RAB8A	79.3	-0.96	0.36	-2.65	7.94E-03	4.82E-02

Supplementary Table S8 (Continued). DE genes in Myogel+Fibrin+ PDMS vs.PDMS.

Genes	*baseMean*	*log2FoldChange*	*lfcSE*	*stat*	*pvalue*	*padj*
TFG	29.09	-1.24	0.47	-2.66	7.91E-03	4.82E-02
LSM10	67.44	0.93	0.35	2.65	7.99E-03	4.83E-02
MRPS31	94.51	0.91	0.34	2.65	8.00E-03	4.83E-02
C12orf57	184.45	0.67	0.25	2.65	8.04E-03	4.85E-02
HNRNPA0	21.81	-1.36	0.51	-2.65	8.09E-03	4.87E-02
RBM19	49.01	0.98	0.37	2.65	8.11E-03	4.87E-02
IMP3	49.78	-1.11	0.42	-2.64	8.18E-03	4.91E-02
RBM22	74.06	-0.94	0.35	-2.64	8.24E-03	4.93E-02
SEC63	35.73	-1.12	0.43	-2.64	8.25E-03	4.93E-02
ATP5F1B	118.08	-0.77	0.29	-2.64	8.35E-03	4.98E-02

Supplementary Table S8 (Continued). DE genes in Myogel+Fibrin+ PDMS vs.PDMS.

Genes	*baseMean*	*log2FoldChange*	*lfcSE*	*stat*	*pvalue*	*padj*
SPATS2L	444.34	1.11	0.19	5.72	1.07E-08	2.05E-04
CTTN	78.07	1.9	0.34	5.56	2.64E-08	2.54E-04
SQLE	291.71	1.14	0.21	5.33	9.58E-08	6.14E-04
ATAD2	351.44	1.19	0.23	5.08	3.83E-07	1.47E-03
SOX4	1155.93	1.32	0.26	5.11	3.29E-07	1.47E-03
CTSV	90.45	-1.41	0.29	-4.83	1.35E-06	4.33E-03
DYNC1I2	95.3	1.29	0.28	4.62	3.76E-06	8.21E-03
HIST1H1B	9.86	-2.23	0.48	-4.66	3.13E-06	8.21E-03
ING2	577.29	1	0.22	4.62	3.84E-06	8.21E-03
MARCKSL1	75.83	-1.5	0.33	-4.56	5.18E-06	9.06E-03
PLEKHA3	195.44	1.1	0.24	4.56	5.07E-06	9.06E-03
TSPYL1	15.28	2.06	0.46	4.51	6.45E-06	9.98E-03
XPA	166.06	1.04	0.23	4.5	6.74E-06	9.98E-03
SUDS3	1097.66	1.1	0.25	4.44	8.81E-06	1.21E-02
GCM1	33.43	1.83	0.41	4.42	9.70E-06	1.21E-02
PGAP2	22.65	-1.84	0.42	-4.42	1.01E-05	1.21E-02
TNRC6A	82.3	1.53	0.35	4.38	1.20E-05	1.36E-02
EML4	824.34	1.03	0.24	4.36	1.31E-05	1.40E-02
FERMT2	257.51	1.02	0.24	4.34	1.42E-05	1.44E-02
NUCKS1	923.86	0.78	0.18	4.3	1.71E-05	1.65E-02
AC098934.1	1076.76	1.21	0.28	4.26	2.08E-05	1.82E-02
GMPS	70.96	-1.28	0.3	-4.26	2.02E-05	1.82E-02
TOB1	21.54	1.88	0.45	4.22	2.45E-05	2.05E-02
ELF2	304.4	0.92	0.22	4.2	2.72E-05	2.18E-02
VTRNA1-1	10.36	2.01	0.48	4.17	3.10E-05	2.39E-02
SRRM2	1508.96	1.06	0.26	4.14	3.44E-05	2.55E-02
RC3H1	85.1	1.58	0.38	4.13	3.66E-05	2.61E-02
ZNRD2	1050.51	0.95	0.23	4.11	3.92E-05	2.69E-02
SON	728.55	1.52	0.37	4.09	4.37E-05	2.90E-02
CTNNAL1	752.13	0.84	0.21	4.02	5.83E-05	3.26E-02
FAM133B	99.74	1.45	0.36	4.01	6.14E-05	3.26E-02
ITPKC	15.76	1.69	0.42	4	6.24E-05	3.26E-02
PRPF6	696.37	0.9	0.22	4.05	5.22E-05	3.26E-02
SLC25A5	95.9	-1.06	0.26	-4.04	5.41E-05	3.26E-02

Supplementary Table S9. DE genes in Myogel+PDMS vs.Control.

Genes	*baseMean*	*log2FoldChange*	*lfcSE*	*stat*	*pvalue*	*padj*
SPRY1	13.94	1.73	0.43	4	6.26E-05	3.26E-02
SRSF7	75.93	-1.18	0.29	-4.03	5.49E-05	3.26E-02
TDG	289.31	1.18	0.29	4.03	5.66E-05	3.26E-02
ITGB1	1074.98	1.13	0.29	3.95	7.71E-05	3.45E-02
ITPR2	54.21	1.28	0.32	3.97	7.21E-05	3.45E-02
RAB2A	609.56	0.87	0.22	3.97	7.19E-05	3.45E-02
RPL10	235.3	-1.13	0.29	-3.96	7.65E-05	3.45E-02
UPF2	494	1.27	0.32	3.96	7.62E-05	3.45E-02
VPS4B	199.6	1.37	0.35	3.96	7.58E-05	3.45E-02
NMT1	99.74	1.31	0.33	3.94	8.18E-05	3.50E-02
PSMC1	52.46	1.54	0.39	3.94	8.00E-05	3.50E-02
LDHB	290.04	-0.86	0.22	-3.92	8.85E-05	3.70E-02
KLHL23	272.3	0.86	0.22	3.91	9.06E-05	3.71E-02
SPTY2D1	62.91	1.33	0.34	3.91	9.39E-05	3.76E-02
AL139317.2	17.97	1.79	0.46	3.9	9.67E-05	3.80E-02
CNOT4	91.81	1.1	0.28	3.89	9.98E-05	3.84E-02
RFC3	263.04	1.34	0.35	3.86	1.11E-04	4.19E-02
SEC62	442.71	1.22	0.32	3.84	1.21E-04	4.47E-02
PTRH1	460.7	1	0.26	3.84	1.23E-04	4.48E-02

Supplementary Table S9 (Continued). DE genes in Myogel+PDMS vs.Control.

Day 1	***RNA Con.***	***Day 3***	***RNA Con.***
Sample ID	ng/ul	Sample ID	ng/ul
PDMS-1	32.43	PDMS-1	75.13
PDMS-2	37.55	PDMS-2	104.65
PDMS-3	34.19	PDMS-3	108.54
Control-1	51.27	Control-1	205.31
Control-2	50.65	Control-2	197.23
Control-3	59.3	Control-3	175.76
Myogel-1	53.93	Myogel-1	188.68
Myogel-2	61.84	Myogel-2	201.87
Myogel-3	59.79	Myogel-3	173.6
Myogel+fibrin-1	10.58	Myogel+Fibrin-1	123.89
Myogel+fibrin-2	11.73	Myogel+Fibrin-2	141.33
Myogel+fibrin-3	11.08	Myogel+Fibrin-3	177.41
Myogel+PDMS-1	72.28	Myogel+PDMS-1	214.85
Myogel+PDMS-2	65.6	Myogel+PDMS-2	218.7
Myogel+PDMS-3	76.95	Myogel+PDMS-3	214.43
Myogel+fibrin+PDMS-1	8.94	Myogel+Fibrin+PDMS-1	159.8
Myogel+fibrin+PDMS-2	5.57	Myogel+Fibrin+PDMS-2	145.9
Myogel+fibrin+PDMS-3	4.53	Myogel+Fibrin+PDMS-3	168.2

Supplementary Table S10. RNA Concentration in day 1 and day 3. Three replicates from each condition, (n=3).
